# Astrocyte Elevated Gene-1 as a Novel Clinicopathological and Prognostic Biomarker for Gastrointestinal Cancers: A Meta-Analysis with 2999 Patients

**DOI:** 10.1371/journal.pone.0145659

**Published:** 2015-12-28

**Authors:** Yihuan Luo, Xin Zhang, Zhong Tan, Peirong Wu, Xuelian Xiang, Yiwu Dang, Gang Chen

**Affiliations:** Department of Pathology, First Affiliated Hospital of Guangxi Medical University, Nanning, Guangxi Zhuang Autonomous Region, People’s Republic of China; H. Lee Moffitt Cancer Center & Research Institute, UNITED STATES

## Abstract

**Background:**

There have been numerous articles as to whether the staining index (SI) of astrocyte elevated gene-1 (AEG-1) adversely affects clinical progression and prognosis of gastrointestinal cancers. Nevertheless, controversy still exists in terms of correlations between AEG-1 SI and clinicopathological parameters including survival data. Consequently, we conducted a comprehensive meta-analysis to confirm the role of AEG-1 in clinical outcomes of gastrointestinal carcinoma patients.

**Methods:**

We performed a comprehensive search in PubMed, ISI Web of Science, Cochrane Central Register of Controlled Trials, EMBASE, Science Direct, Wiley Online Library, China National Knowledge Infrastructure (CNKI), WanFang and Chinese VIP databases. STATA 12.0 (STATA Corp., College, TX) was used to analyze the data extracted from suitable studies and Newcastle-Ottawa Scale was applied to assess the quality of included articles.

**Results:**

The current meta-analysis included 2999 patients and our results suggested that strong associations emerged between AEG-1 SI and histological differentiation (OR = 2.129, 95%CI: 1.377–3.290, P = 0.001), tumor (T) classification (OR = 2.272, 95%CI: 1.147–4.502, P = 0.019), lymph node (N) classification (OR = 2.696, 95%CI: 2.178–3.337, P<0.001) and metastasis (M) classification (OR = 3.731, 95%CI: 2.167–6.426, P<0.001). Furthermore, high AEG-1 SI was significantly associated with poor overall survival (OS) (HR = 2.369, 95%CI: 2.005–2.800, P<0.001) and deteriorated disease-free survival (DFS) (HR = 1.538, 95%CI: 1.171–2.020, P = 0.002). For disease-specific survival (DSS) and relapse-free survival (RFS), no statistically significant results were observed (HR = 1.573, 95%CI: 0.761–3.250, P = 0.222; HR = 1.432, 95%CI: 0.108–19.085, P = 0.786). Subgroup analysis demonstrated that high AEG-1 SI was significantly related to poor prognosis in esophageal squamous cell carcinoma (ESCC) (HR = 1.715, 95%CI: 1.211–2.410, P = 0.002), gastric carcinoma (GC) (HR = 2.255, 95%CI: 1.547–3.288, P<0.001), colorectal carcinoma (CRC) (HR = 2.922, 95%CI: 1.921–4.444, P<0.001), gallbladder carcinoma (GBC) (HR = 3.047, 95%CI: 1.685–5.509, P<0.001), hepatocellular carcinoma (HCC) (HR = 2.245, 95%CI: 1.620–3.113, P<0.001), pancreatic adenocarcinoma (PAC) (HR = 2.408, 95%CI: 1.625–3.568, P<0.001).

**Conclusions:**

The current meta-analysis indicated that high AEG-1 SI might be associated with tumor progression and poor survival status in patients with gastrointestinal cancer. AEG-1 might play a vital role in promoting tumor aggression and could serve as a potential target for molecular treatments. Further clinical trials are needed to validate whether AEG-1 SI provides valuable insights into improving treatment decisions.

## Introduction

Cancers in digestive system can be mainly divided into esophageal cancer (EC), gastric carcinoma (GC), colorectal carcinoma (CRC), gallbladder carcinoma (GBC), hepatocellular carcinoma (HCC) and pancreatic adenocarcinoma (PAC). According to global cancer statistics in 2012, HCC and GC are identified as the second and third most frequently diagnosed cancers among men in less developed countries. It is estimated that EC, with highest rates in East Asia, caused 400,200 deaths in 2012 worldwide, while there were 1.4 million cases of CRC patients and 693,900 deaths occurred due to CRC [1]. In spite of advanced techniques of diagnosis and treatments nowadays, approaches to distinguish tumor progression and prognosis of patients with gastrointestinal cancers still need to improve. Hence, it is urgently demanded to find better makers, which can reflect the clinicopathological alterations and predicate prognosis accurately in the early stages of tumors.

Astrocyte elevated gene-1 (AEG-1), also known as metadherin (MTDH) [2] or LYsine-RIch CEACAM1 coisolated (LYRIC) [3], was first identified in 2002 as a novel protein induced in primary human fetal astrocytes infected by human immunodeficiency virus 1 (HIV)-1 and tumor necrosis factor-α (TNF-α) [4]. The AEG-1 gene is an oncogene, which is located at chromosome 8q22 [5], and it is observed that elevated expression of AEG-1 promoted tumor proliferation, progression or metastasis in multiple carcinomas such as EC [6], HCC [7], neuroblastoma [8], breast cancer [9], prostate cancer [10] and malignant glioma [11]. In addition, AEG-1 could activate multiple molecular mechanisms to exert its functions, including nuclear factor κ-B (NF-κB) [12], phosphatidylinositol 3-kinase (PI3K)/Akt and c-Myc [13, 14], Wnt/b-catenin [15], extracellular signal-regulated kinase (ERK) [7], activator protein 1 (AP-1) [10] and non-thyroidal illness syndrome (NTIS) [16]. Also, it was reported that AEG-1 could increase the expression of angiopoietin-1, matrix metalloprotease-2 (MMP2), hypoxia-inducible factor 1-α (HIF-α) and Tie2, which are essential in angiogenesis [17].

The evidences above reveal that AEG-1 is involved in the process of tumor proliferation, infiltration and metastasis. Furthermore, a meta-analysis have been conducted to explore the relationships between AEG-1 staining index (SI) and clinicopathological features in squamous cell carcinoma (SCC) [18], which offered clear information regarding the influence of AEG-1 on SCC. Nonetheless, the association of AEG-1 with clinical prognosis has not been estimated and its limited sample size devalued the analysis to some extent. Meanwhile, no consistent conclusion was reached on the possible role that AEG-1 might play in the progression and prognosis of gastrointestinal cancers. Therefore, we reviewed the observational studies available quantitatively and performed the current meta-analysis in an attempt to investigate the clinicopathological and prognostic significance of AEG-1 in patients with gastrointestinal cancers.

## Materials and Methods

### Literature Reviewing and Selecting

Initially, we performed an electronic search to identify all literature related to AEG-1 SI in patients with cancers in following databases: PubMed, ISI Web of Science, Cochrane Central Register of Controlled Trials, EMBASE, Science Direct, Wiley Online Library, China National Knowledge Infrastructure (CNKI), WanFang and Chinese VIP. The search strategy consisted of the combinations of “AEG1”, “AEG-1”, “astrocyte elevated gene-1”, “MTDH”, “metadherin”, “LYRIC” and “tumor”, “cancer”, “neoplas*”, “malignan*”, “carcinoma”. Reviews and references related were also scrutinized. The closing date for our search was August 14, 2015, which denoted that no literature after the time point would be included.

Two independent investigators (YiHuan Luo, Zhong Tan) reviewed the literature quantitatively with the same multi-step process. Firstly, the abstracts were screened to exclude the ineligible studies which were irrelevant or duplicate. Then, full-text contents of the remaining studies were further reviewed by the investigators independently to decide whether to subsume in accordance with the inclusion criteria listed below: (1) The samples should be collected form patients with gastrointestinal cancers; (2) The studies should explore the correlation between AEG-1 SI and survival data, be published in whether English or Chinese, and detect the AEG-1 levels by immunohistochemistry (IHC); (3) The studies should offer available data to calculate hazard ratios (HRs) value and its 95% confidence interval (95% CI). In addition, trails using either animals or cell lines, reviews, case reports and letters were excluded. If survival analyses were displayed in the articles but proved insufficient to calculate the HR value, we would strive to contact the authors to obtain primary survival data whenever and wherever possible. To avoid duplications of data, only the study with the most complete data would be included when there existed different studies investigating the same or overlapping cohort of patients. Finally, controversies were resolved by a third reviewer (Gang Chen).

### Data Extraction

Data were extracted carefully from eligible studies by two investigators (YiHuan Luo and Zhong Tan) independently and consistency was reached in all items. The extracted characteristics included the first author’s name, published year, cancer type, country and number of the patients, antibody, cut-off value for AEG-1 positivity, blinding of AEG-1 measurements, follow-up times, analysis types and prognostic data. To evaluate the relationship between AEG-1 and tumor aggressivity, the following clinicopathological parameters were extracted: differentiation degree, tumor (T) classification, lymph node (N) classification, metastasis (M) classification. Disagreements would be discussed by two investigators till consensus was achieved.

### Quality Assessment

Although there was no universally recognized rating scale for meta-analysis to evaluate observational studies scientifically and quantitatively, two investigators (YiHuan Luo and Xin Zhang) independently assessed the methodological quality of included studies by reviewing and scoring each studies according to Newcastle–Ottawa Scale (NOS) [19]. The scale evaluates the selection of cohorts, the comparability of cohorts and the ascertainment of outcomes. Each study could be credited at most one star for every numbered item in the Selection and Outcomes section. Meanwhile, the Comparability section was entitled to a maximum of two stars. The final stars were calculated in the end, which could not exceed an overall of nine stars. The more stars a study collected, the better methodological quality it presented.

### Statistical Methods

The odds ratio (OR) value was adopted to estimate the associations of AEG-1 SI with clinicopathological features in patients with gastrointestinal cancers. When OR >1, it indicated that high AEG-1 SI was more likely to correlate with poorer degree of histological differentiation and more advanced stage of TNM classification.

To evaluate the impact of AEG-1 SI on patients’ survival, HR value and its 95% CI of each single study were extracted and later combined. The simplest method was to extract HR values and their 95% CIs directly from the studies. For the studies which only presented the Kaplan-Meier curves or primary data, we calculated the HR value by the methods that Jayne F Tierney had described [20], i.e. using either the software Engauge Digitizer version 4.1 (http://digitizer.sourceforge.net/) or SPSS20.0. High AEG-1 SI indicated poor prognosis if a pooled HR>1 was observed. For heterogeneity analysis, Cochrane Q test (Chi-squared test) was conducted to measure the potential heterogeneity among the included studies. If no statistically significant heterogeneity (P>0.05) existed, a fixed-effect model (Mantel-Haenszel method) was conducted to combine the HR values. Otherwise, a random-effect model (DerSimonian and Laird method) would be employed. Meanwhile, funnel plots were applied to examine the publication bias. All the statistical analyses above were performed on STATA12.0 (STATA Corp., College, Texas), and it was considered statistically significant when a two-sided *P* value was less than 0.05.

## Results

### Summarized Characteristics of Eligible Literature

**Fig 1:**
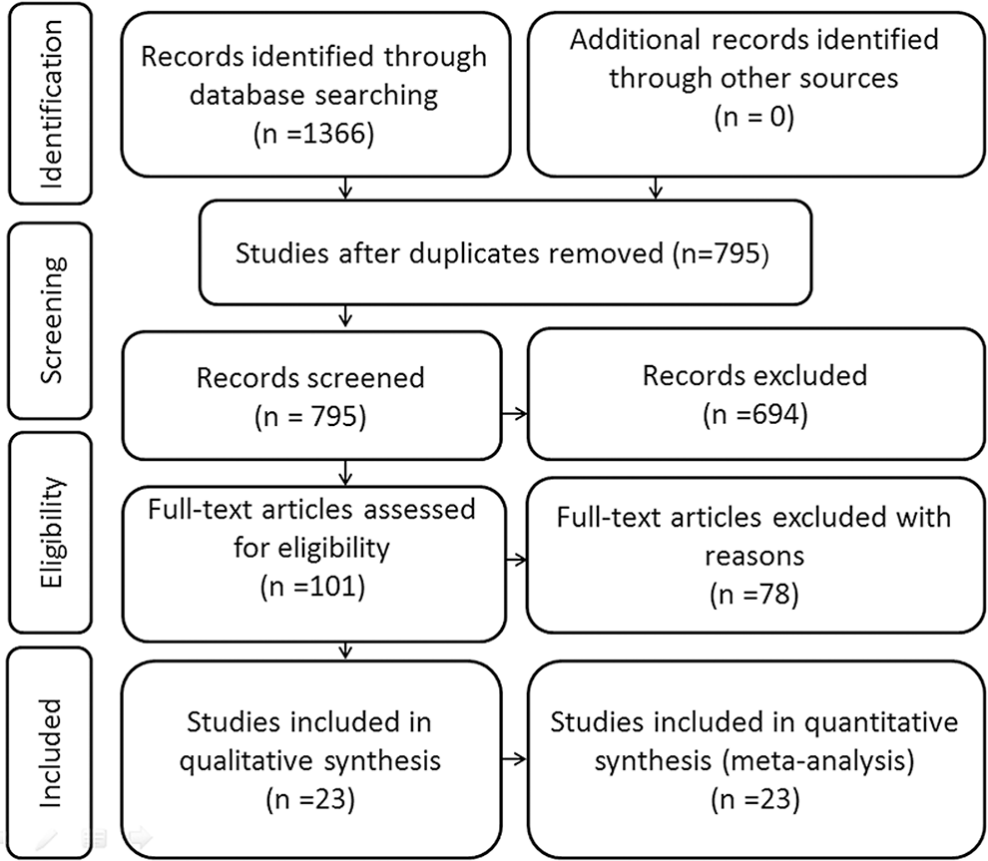
Flow diagram of literature reviewing and selection.

The primary search identified a total of 795 studies and we evaluated 100 of them in full text. Among full-text studies, 74 studies were excluded for the lack of survival data and 3 studies were excluded for failure to estimate HR (Fig 1). Finally, 23 independent studies (n = 2999 patients), published from 2009 to 2015, were included in our meta-analysis [6, 18, 21–41] except that partial data of one study were excluded due to failure to estimate reasonable HR value [29]. The main characteristics of the included studies were listed in Table 1. The number of the patients ranged from 41 to 520. In the current meta-analysis, 16 studies provided available information for histological differentiation, 13 studies for T classification, 15 studies for N classification and 11 studies for M classification. Meanwhile, this meta-analysis included 19 studies evaluable for overall survival (OS), 3 studies for disease-free survival (DFS), 2 studies for disease-specific survival (DSS) and 2 studies for relapse-free survival (RFS). Fifteen studies assessed the IHC results with blind reading, while 8 studies were not reported. The follow-up times varied from 18 to 193 months. Among all 23 studies, 18 provided available survival data of multivariate analyses [18, 21–28, 30, 34–41]and 5 contained only survival curves [6, 29, 31–33].

**Table 1:** Main characteristics of the included studies.

First author	Year	Country	Cancer types	N (M/F)	Staining for high AEG-1 SI	Blinded reading	Follow-up (Months)	Analysis types for survival	HR (95%CI)	Quality score
Yu CP [6]	2009	China	ESCC	168 (129/39)	Staining index score ≥ 6	Yes	80*	OS	1.655 (1.095–2.495)	6
Song HT [21]	2010	China	CRC	146 (90/56)	Staining index score ≥ 4	Yes	70*	OS	3.174 (1.698–5.931)	8
Liu DC [23]	2011	China	GBAC	67 (19/48)	Positive cells ≥ 25% and staining intensity ≥ 2	NR	18*	OS	3.047 (1.420–6.560)	6
Sun W [24]	2011	China	GBC	41 (16/25)	Staining index score ≥ 4	NR	60	OS	3.046 (1.195–7.761)	8
Xu JB [22]	2011	China	GC	101 (61/44)	Staining index score ≥ 3	Yes	60	OS	2.110 (1.640–2.780)	8
Wang N [26]	2012	China	CC	196 (94/102)	positive cells ≥ 25% and staining intensity ≥ 2	Yes	103	OS	2.890 (1.060–6.690)	7
Jiang T [25]	2012	China	CRC	520 (291/229)	Positive cells ≥ 1%	NR	70*	DSS	2.351 (1.422–4.537)	7
Gong ZB [27]	2012	China	HCC	73 (62/11)	Staining index score ≥ 6	Yes	60*	OS	7.314 (1.848–28.398)	7
Ahn S [28]	2013	Korea	HCC	288 (237/51)	Staining index score ≥ 7	Yes	126	DFS	1.451 (1.082–1.944)	7
								DSS	1.118 (0.746–1.673)	
Chen XJ [38]	2013	China	HCC	107 (73/34)	Staining index score ≥ 4	NR	72*	DFS	3.431 (1.254–7.318)	7
Shi S [37]	2013	China	PAC	89 (52/37)	Staining index score ≥ 7	Yes	33	OS	2.638 (1.537–4.528)	7
Casimiro S [30]	2014	Portugal	CRC	85 (52/33)	Positive cells > 10% and	Yes	60*	OS	4.570 (1.390–14.990)	7
					staining intensity ≥ 2			RFS	5.070 (1.970–13.060)	
Li SH [31]	2014	China	GC	216 (80/136)	Staining index score ≥ 2	Yes	80*	OS	3.345 (3.165–5.170)	6
Dong LP [32]	2014	China	GC	119 (67/52)	Staining index score ≥ 2	Yes	60	OS	1.580 (0.860–2.900)	6
Li GH [33]	2014	China	GC	93 (64/29)	Staining index score ≥ 3	NR	80*	OS	1.520 (0.585–3.970)	6
Li Q [39]	2014	China	HCC	87 (44/43)	Staining index score ≥ 2	Yes	60	OS	2.190 (1.240–3.950)	7
Huang Y [34]	2014	China	PDAC	105 (63/42)	Staining index score ≥ 4	Yes	35*	OS	2.173 (1.288–4.055)	7
Gnosa S [29]	2014	Sweden	RC	74 (NR)	Staining intensity score ≥ 2	Yes	193	RFS	0.360 (0.085–1.525)	6
								DFS	0.790 (0.200–3.110)	
Zhang W [41]	2015	China	CAC	60 (32/28)	Staining index score ≥ 3	NR	70	OS	5.473 (1.068–28.053)	7
Wang B [35]	2015	China	CRC	50 (29/21)	NR	NR	50*	OS	1.228 (0.419–3.594)	7
Yang CC [18]	2015	China	ESCC	77 (NR)	Staining index score ≥ 6	Yes	80*	OS	1.852 (1.013–3.387)	7
Jung HL [36]	2015	Korea	HCC	85 (69/16)	Staining index score ≥ 3	Yes	130*	OS	4.756 (1.697–13.329)	7
Li JM [40]	2015	China	HCC	152 (132/20)	Staining index score ≥ 7	NR	80*	OS	1.736 (1.106–2.726)	7

N (M/F): number (male/female); HR: hazard ratio; CI: confidence interval; ESCC: esophageal squamous cell carcinoma; C(R)C: colon (rectal) carcinoma; GB(A)C: gallbladder (adeno) carcinoma; GC: gastric carcinoma; P(D)AC: pancreatic (ductal) adenocarcinoma; HCC: hepatocellular carcinoma; OS: overall survival; DSS: disease-specific survival; DFS: disease-free survival; RFS: relapse-free survival; NR: not report

*: approximate times extracted form survival curve.

### Quality Assessment

The information of scoring was summarized in Table 1. For quality assessment, the highest score was 9 and 5 or higher score would be regarded as high methodological quality. In the current meta-analysis, each study included in our meta-analysis was with a score ≥6, which ensured its eligibility in terms of methodological quality.

### Associations of AEG-1 SI with clinicopathological features

**Fig 2:**
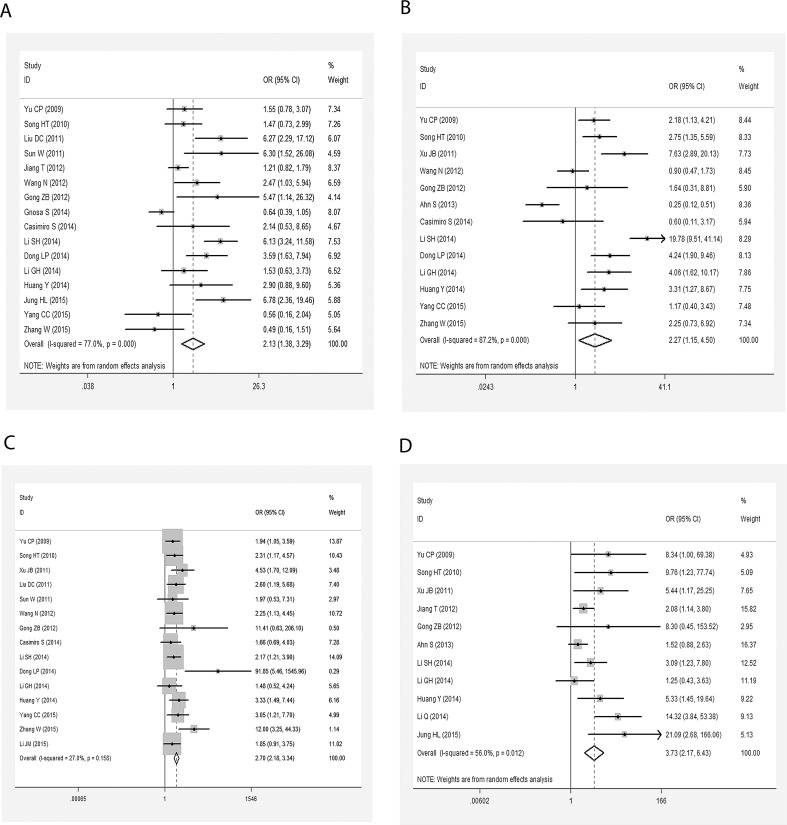
Meta-analysis evaluating the relationships between AEG-1 SI and clinicopathological parameters in patients with gastrointestinal cancer. A, histological differentiation (random effect model); B, T classification (random effect model); C, N classification (fixed effect model); D, M classification (random effect model).

Listed in Table 2 were the main statistical results evaluating the effects of AEG-1 SI on clinicopathological features of patients with gastrointestinal cancer. Overall, 16 studies (n = 2398 patients) estimated the relationship between AEG-1 SI and histological differentiation. The pooled OR was 2.129 (95%CI: 1.377–3.290, P = 0.001) (Fig 2A), suggesting that high AEG-1 SI closely correlated with poor degree of histological differentiation. In addition, our meta-analysis unveiled the significant associations between AEG-1 SI and T classification (n = 1637 patients), N classification (n = 1751 patients) and M classification (n = 1886 patients). The combined OR were 2.272 (95%CI: 1.147–4.502, P = 0.019) (Fig 2B), 2.696 (95%CI: 2.178–3.337, P<0.001) (Fig 2C) and 3.731 (95%CI: 2.167–6.426, P<0.001) (Fig 2D), respectively (Table 3). The results above indicated that high AEG-1 SI correlated with worsened situations of tumor invasion, lymph node metastasis and distant metastasis.

**Table 2:** Combinations of data evaluating the relationships between AEG-1 SI and clinicopathological parameters.

Clinicopathological parameters	Studies (n)	Test group		Control group		Meta-analysis model	OR (95%CI)	*P*
		Events	Total	Events	Total			
Histological differentiation (Poorly/Well-Moderately)	16 (2398)	530	816	779	1582	Random	2.129 (1.377–3.290)	0.001
T classification (T3-T4/T1-T2)	13 (1637)	632	930	395	707	Random	2.272 (1.147–4.502)	0.019
N classification (N1-N3/N0)	15 (1751)	605	846	461	905	Fixed	2.696 (2.178–3.337)	<0.001
M classification (M1/M0)	11 (1886)	214	287	782	1599	Random	3.731 (2.167–6.426)	<0.001

OR: odds ratio; CI: confidence interval.

**Table 3:** Meta-analysis of included studies and subgroup analysis assessing the association between AEG-1 SI and clinical prognosis.

Groups		Studies (n)	Pooled HR with fixed model (95%CI)	Test for heterogeneity		Pooled HR with random model (95%CI)
				Q	P	
Overall survival (OS)		19 (2010)	2.412 (2.136–2.723)	26.18	0.096	2.369 (2.005–2.800)
Cancer types						
	ESCC	2 (245)	1.715 (1.211–2.410)	0.09	0.763	1.715 (1.211–2.410)
	CRC	5 (537)	2.922 (1.921–4.444)	3.68	0.451	2.922 (1.921–4.444)
	GBC	2 (108)	3.047 (1.685–5.509)	0	1.000	3.047 (1.685–5.509)
	GC	4 (529)	2.545 (2.148–3.015)	10.18	0.017	2.255 (1.547–3.288)
	HCC	4 (397)	2.245 (1.620–3.113)	6.17	0.104	2.656 (1.554–4.540)
	PAC	2 (194)	2.408 (1.625–3.568)	0.23	0.630	2.408 (1.625–3.568)
Disease-free survival (DFS)		3 (469)	1.538 (1.171–2.020)	4.24	0.120	1.683 (0.878–3.227)
Disease-specific survival (DSS)		2 (808)	1.425 (1.023–1.985)	4.25	0.039	1.573 (0.761–3.250)
Relapse-free survival (RFS)		2 (159)	2.291 (1.039–5.053)	9.02	0.003	1.432 (0.108–19.085)

ESCC: esophageal squamous cell carcinoma; CRC: colorectal carcinoma; GBC: gallbladder carcinoma; GC: gastric carcinoma; HCC: hepatocellular carcinoma; PAC: pancreatic adenocarcinoma.

### Impact of AEG-1 SI on Survival

**Fig 3:**
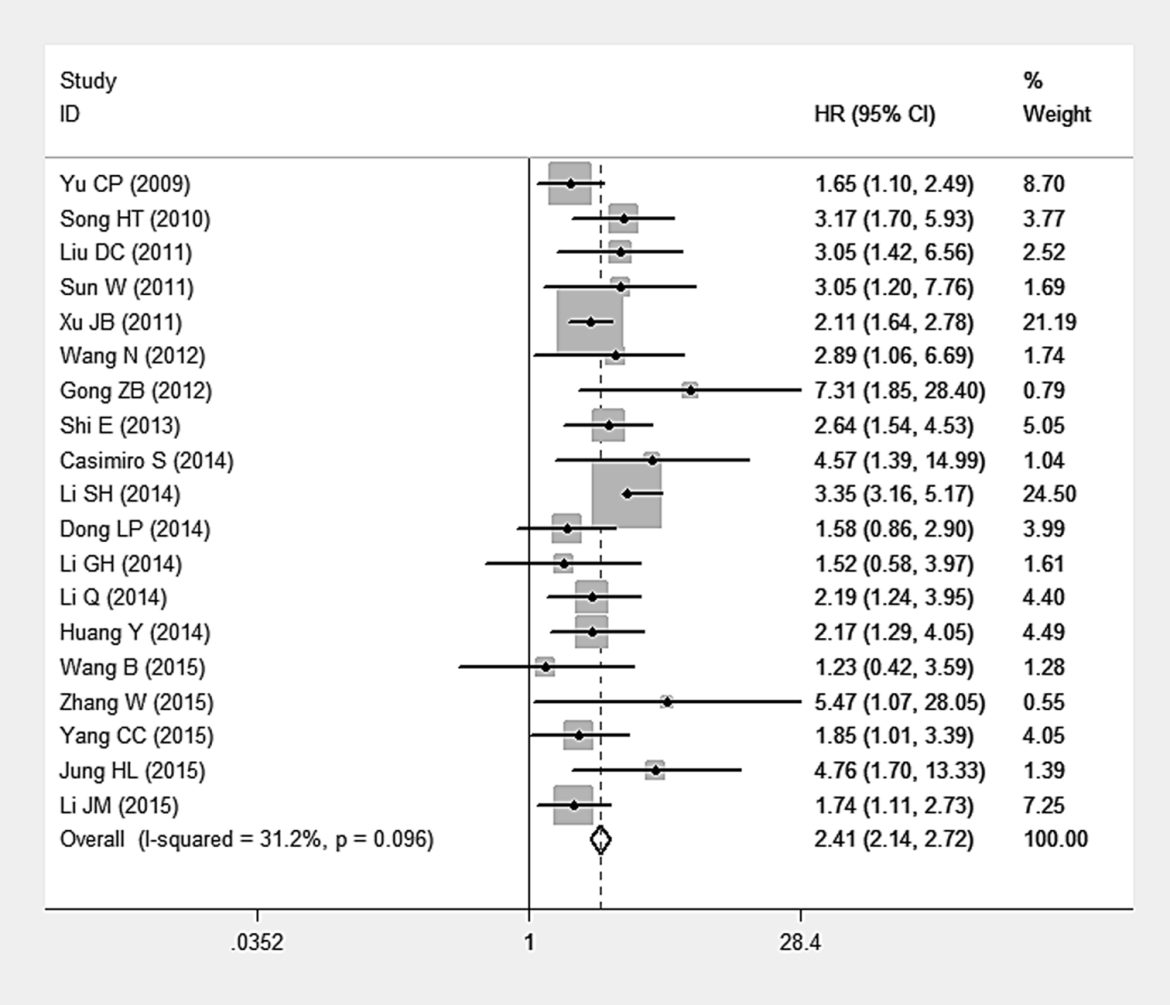
Meta-analysis of included studies evaluating the association between AEG-1 SI and overall survival (OS) (fixed effect model).

**Fig 4:**
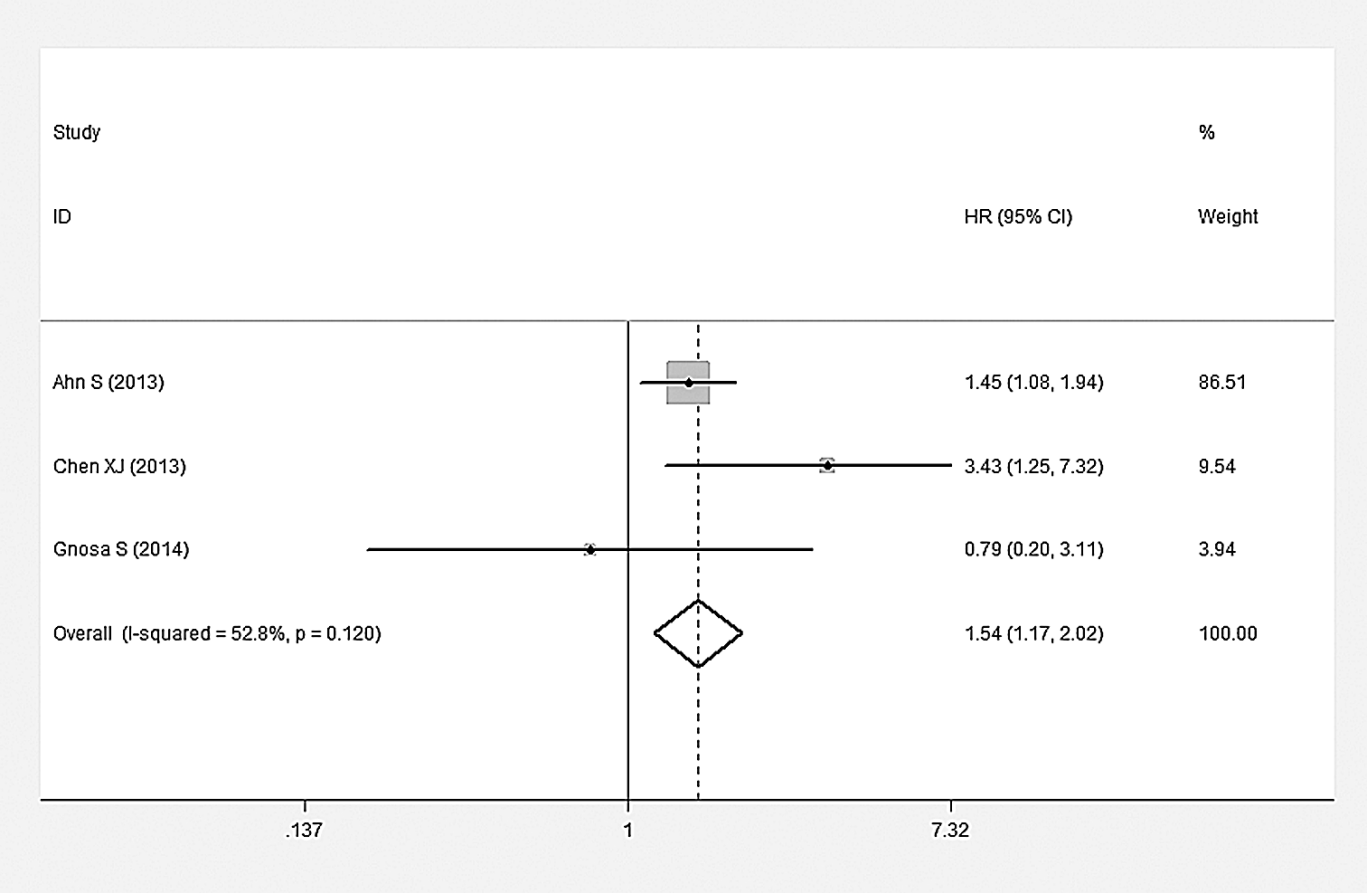
Meta-analysis of included studies evaluating the association between AEG-1 SI and disease-free survival (DFS) (fixed effect model).

**Fig 5:**
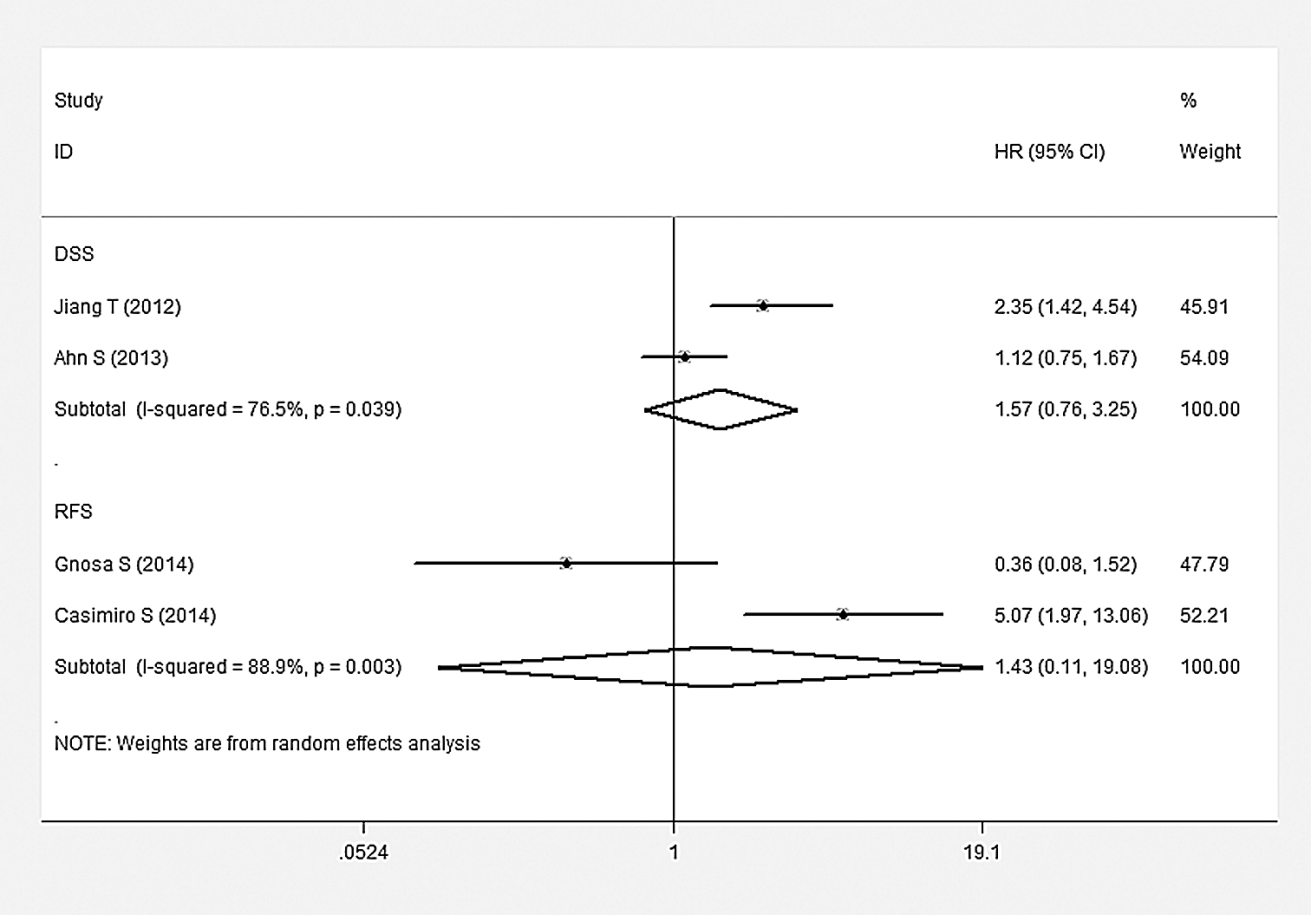
Meta-analysis of included studies evaluating the associations of AEG-1 SI with disease-specific survival (DSS) (random effect model) and relapse survival (RFS) (random effect model).

The main combined results of the effects of AEG-1 SI on survival were outlined in Table 3. According to our meta-analysis, there were statistically significant associations of AEG-1 SI with OS and DFS. The pooled HRs for OS and DFS were 2.412 (95%CI: 2.136–2.723, P<0.001) (Fig 3) and 1.538 (95%CI: 1.171–2.020, P = 0.002) (Fig 4), respectively. However, no statistically significant association was observed between AEG-1 SI and DSS. HR was 1.573 (95%CI: 0.761–3.250, P = 0.222) (Fig 5) when combined with random effect model. Moreover, similar results were found in the case of RFS (HR = 1.432, 95%CI: 0.108–19.085, P = 0.786) (Fig 5). Subgroup analysis demonstrated that high AEG-1 SI was significantly related to poor prognosis in ESCC (HR = 1.715, 95%CI: 1.211–2.410, P = 0.002), GC (HR = 2.255, 95%CI: 1.547–3.288, P<0.001), CRC (HR = 2.922, 95%CI: 1.921–4.444, P<0.001), GBC (HR = 3.047, 95%CI: 1.685–5.509, P<0.001), HCC (HR = 2.245, 95%CI: 1.620–3.113, P<0.001), PAC (HR = 2.408, 95%CI: 1.625–3.568, P<0.001). The results of heterogeneity test were listed in Table 3. Heterogeneity was detected in studies evaluating DSS (Q = 4.25, P = 0.039), RFS (Q = 4.25, P = 0.003) as well as the subgroup of GC (Q = 10.18, P = 0.017). No heterogeneity existed among other groups of studies (P>0.05).

### Publication Bias

**Fig 6:**
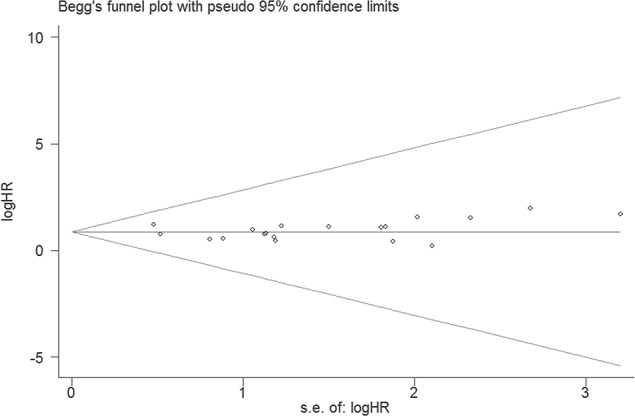
A funnel plot was used to estimate potential publication bias. (Begg’s method was employed.)

**Fig 7:**
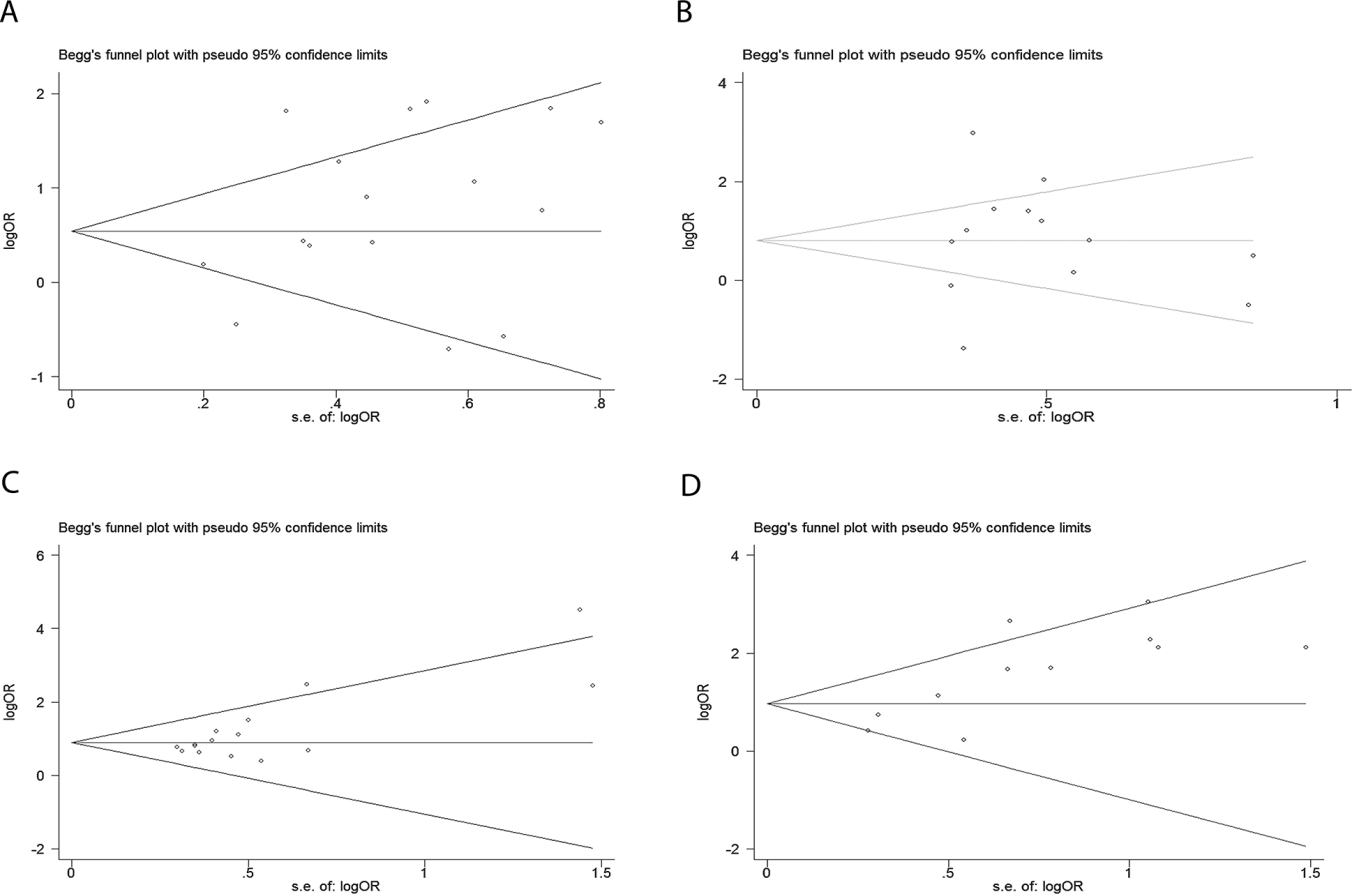
Funnel plots were applied to estimate potential publication bias. A, histological differentiation. B, T classification. C, N classification. D, M classification. (Begg’s method was employed.)

We did not perform Begg’s test for DFS, DSS or RFS because of the limited scale of studies included. For OS, no publication bias appeared among 19 studies included in our meta-analysis according to funnel plots (Fig 6, P = 0.054). For the studies evaluating the associations of AEG-1 SI with clinicopathological features, no publication bias was observed in the group of histological differentiation (Fig 7A, P = 0.322), T classification (Fig 7B, P = 1.000), N classification (Fig 7C, P = 0.067) or M classification (Fig 7D, P = 0.102).

## Discussion

AEG-1 has attracted attention of numerous researchers since 2002 for its potential vital connection with tumor aggression and prognosis. Recently, several studies demonstrated that overexpression of AEG-1 was associated with clinical prognosis in different cancers [6, 21, 22, 42–44]. In addition, reviews have comprehensively summarized the crucial role that AEG-1 might play in the prognosis of cancers [45, 46]. In gastrointestinal tumors, lots of research explored the relationship between AEG-1 SI and clinical parameters including follow-up records. Nevertheless, the results were not consistent. We conducted the current quantitative meta-analysis by merging the results of published literature in order to draw a clearer conclusion on the relationships between AEG-1 SI and clinicopathological features as well as prognosis in patients with gastrointestinal tumors.

Recently, a meta-analysis [18], with 10 studies enrolled, demonstrated that values of AEG-1 SI were significantly different between SCC tissues and corresponding normal adjacent tissues, and later discovered that AEG-1 SI was associated with lymph node metastasis, clinical stage and T classification. Indeed, the published meta-analysis has provided readers with the unprecedentedly constructive knowledge over the crucial role that AEG-1 plays in tumor aggression. However, in the previous meta-analysis [18], the prognostic significance of AEG-1 SI was not well established and the scarcity of patients involved added to its deficiencies. In our meta-analysis, we combined the data extracted from 23 studies, involving 2999 patients in total, concerning the associations of AEG-1 SI with clinicopathological features and prognosis. AEG-1 was detected by IHC in all included studies. Meanwhile, all the included studies harbored high methodological qualities (≥5 stars), which were evaluated and verified by NOS. For clinicopathological features, the results of meta-analysis indicated that high AEG-1 SI significantly correlated with deteriorated situations in general, including histological differentiation (OR = 2.129, 95%CI: 1.377–3.290), depth of tumor invasion (OR = 2.272, 95%CI: 1.147–4.502), lymph node metastasis (OR = 2.696, 95%CI: 2.178–3.337) and distant metastasis (OR = 3.731, 95%CI: 2.167–6.426). For the prognosis of patients with gastrointestinal cancers, our meta-analysis suggested that high AEG-1 SI was significantly associated with poor OS (HR = 2.412, 95%CI: 2.136–2.723) and DFS (HR = 1.538, 95%CI: 1.171–2.020), but no statistically significant results were observed in terms of DSS (HR = 1.573, 95%CI: 0.761–3.250) and RFS (HR = 1.432, 95%CI: 0.108–19.085). Meantime, subgroup analyses implied that AEG-1 SI significantly correlated with all the gastrointestinal cancers, including ESCC, CRC, GBC, GC, HCC and PAC. According to our meta-analysis, AEG-1 seems to be a novel biomarker reflecting the status of aggression, which was partly identified by the previous meta-analysis, and predicting the state of prognosis, which underlined the novelty of the current study. Nonetheless, the results mainly presented the circumstances in Asia since the population included in the current meta-analysis mainly consisted of Asians. Whether the novel biomarker would be also suitable for the patients from other regions should be further tested and verified in the oncoming clinical trials in different countries.

In the light of the current meta-analysis, high AEG-1 SI effectively indicated aggravated tumor progression and poor prognosis in gastrointestinal cancers. Moreover, AEG-1 mediates drug resistance via multiple mechanisms [47]. Consequently, down-regulation of AEG-1 mRNA or suppression of relative signal pathways by drugs or siRNA might be a feasible strategy to treat gastrointestinal cancers. Recently, literature have demonstrated that down-regulated AEG-1 expression by siRNA effectively inhibited cell proliferation, invasion and metastasis, induced cell apoptosis and altered cell cycle in gastrointestinal cancers [7, 37, 48–51]. According to Devaraja Rajasekaran et al., combination of nanoparticle-delivered siRNA for AEG-1 and all-trans retinoic acid (ATRA) was an effective strategy to combat HCC [52]. Furthermore, it was reported that perifosine might be a targeted therapy drug which suppressed AEG-1 gene expression by inhibiting Akt/GSK3b/C-MYC signaling pathway in GC [53]. In summary, AEG-1 is a potential target to cure gastrointestinal cancers and more clinic trails for AEG-1 targeted drugs are required to explore therapeutic value of the novel biomarker AEG-1.

Heterogeneity is among the delicate issues that should be dealt with carefully since there exist potential risks for it to adversely affect the combinations of values in meta-analysis [54]. In our meta-analysis, heterogeneity was noted in the studies with DSS and RFS (P<0.05). When combining the OR values for clinicopathological features, heterogeneity also emerged in the groups of histological differentiation, T classification, and M classification (P<0.05). To tackle and minimize the effects of heterogeneity, random effect model was employed for combinations of related data. Publication bias should be considered in meta-analysis given the fact that papers with positive results often share greater chances to get published. According to Begg’s test and funnel plots in our meta-analysis, no publication bias was observed in either studies with OS data or groups of histological differentiation, T classification or N classification (P>0.05).

In spite of heterogeneity and publication bias, there were still some unavoidable limitations in the current meta-analysis. Firstly, only studies reported in either English or Chinese were included, which might result in omitting some qualified papers due to language criteria. Secondly, since some eligible reports did not present the results of multivariate analysis directly, related data needed to be extracted from Kaplan-Meier curves, which might lead to a less accurate HR. Meanwhile, different cut off values for AEG-1 SI in studies were also a factor to produce bias. Moreover, there were different subtypes and locations of various gastrointestinal cancers, which might generate unavoidable clinical biases. Finally, follow-up periods and durations varied considerably and there were censored cases in different studies, which might cause biased HR values to a certain degree.

To conclude, in light of the comprehensive meta-analysis, AEG-1 is actively involved with the process of tumor invasion, lymph node metastasis and distant metastasis. Additionally, elevated AEG-1 SI in clinical tumor samples was significantly related to a shorter OS and DFS time in patients with gastrointestinal cancers. Indeed, AEG-1 plays a vital role in the process of aggression and seems to be an effective biomarker to mirror the prognostic status of gastrointestinal cancer sufferers. Still, further clinical trials with larger scales should be conducted to explore the precise and accurate functions of the novel biomarker AEG-1.

## Supporting Information

S1 File Process of search, data extraction and combination. (PPTX)

S2 File Studies excluded for reasons. (DOCX)

S1 Table PRISMA Checklist. (DOC)
