# Supplementary material for: Astrocyte Elevated Gene-1 as a Novel Clinicopathological and Prognostic Biomarker for Gastrointestinal Cancers: A Meta-Analysis with 2999 Patients
Source: PLoS One. 2015 Dec 28;10(12):e0145659. doi: 10.1371/journal.pone.0145659 (PMC4692396; doi:10.1371/journal.pone.0145659)
Supplement: S1 File — (PPTX) [file pone.0145659.s001.pptx]

## Slide 1
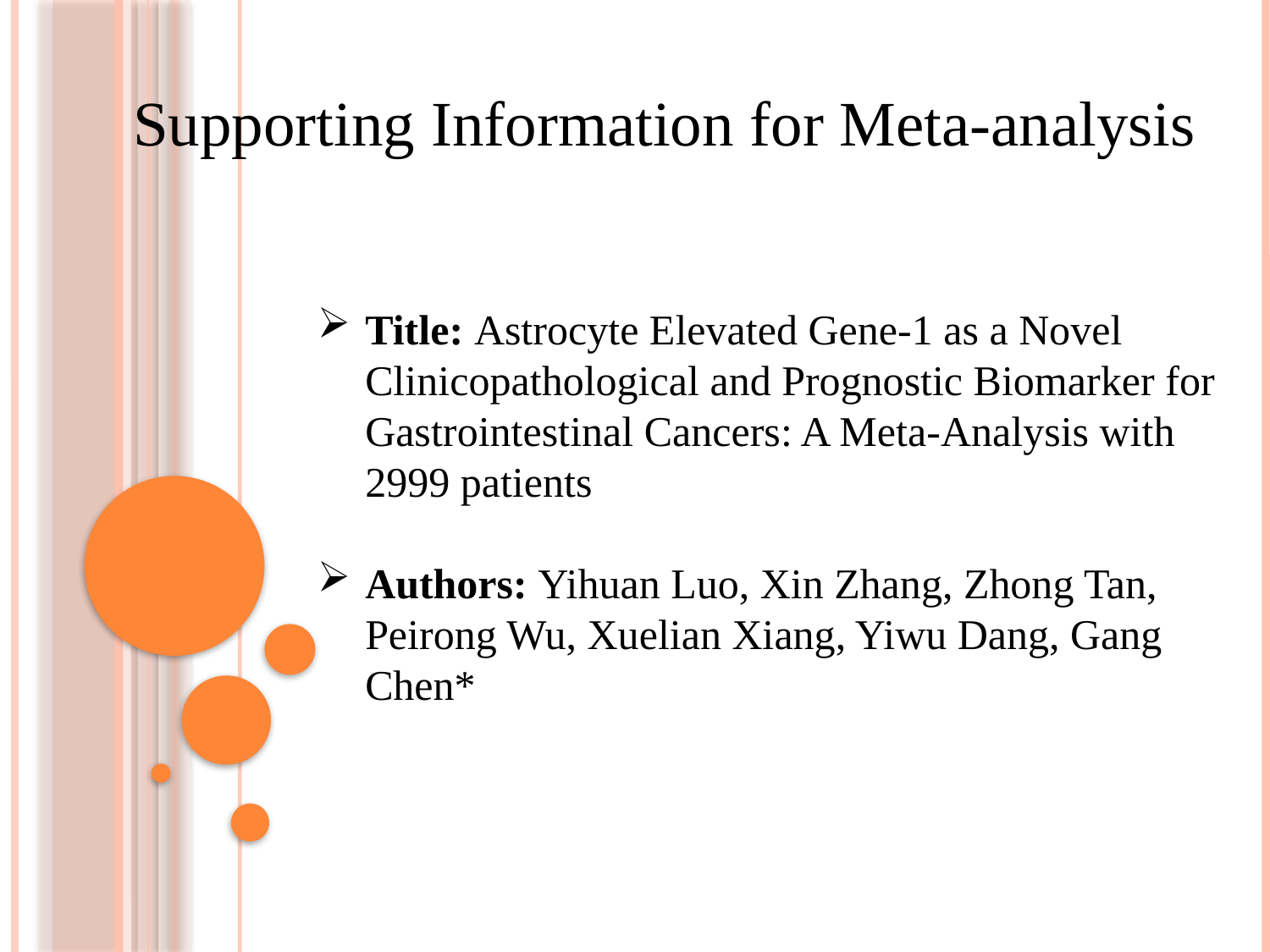

Supporting Information for Meta-analysis
Title: Astrocyte Elevated Gene-1 as a Novel Clinicopathological and Prognostic Biomarker for Gastrointestinal Cancers: A Meta-Analysis with 2999 patients
Authors: Yihuan Luo, Xin Zhang, Zhong Tan, Peirong Wu, Xuelian Xiang, Yiwu Dang, Gang Chen*

## Slide 2
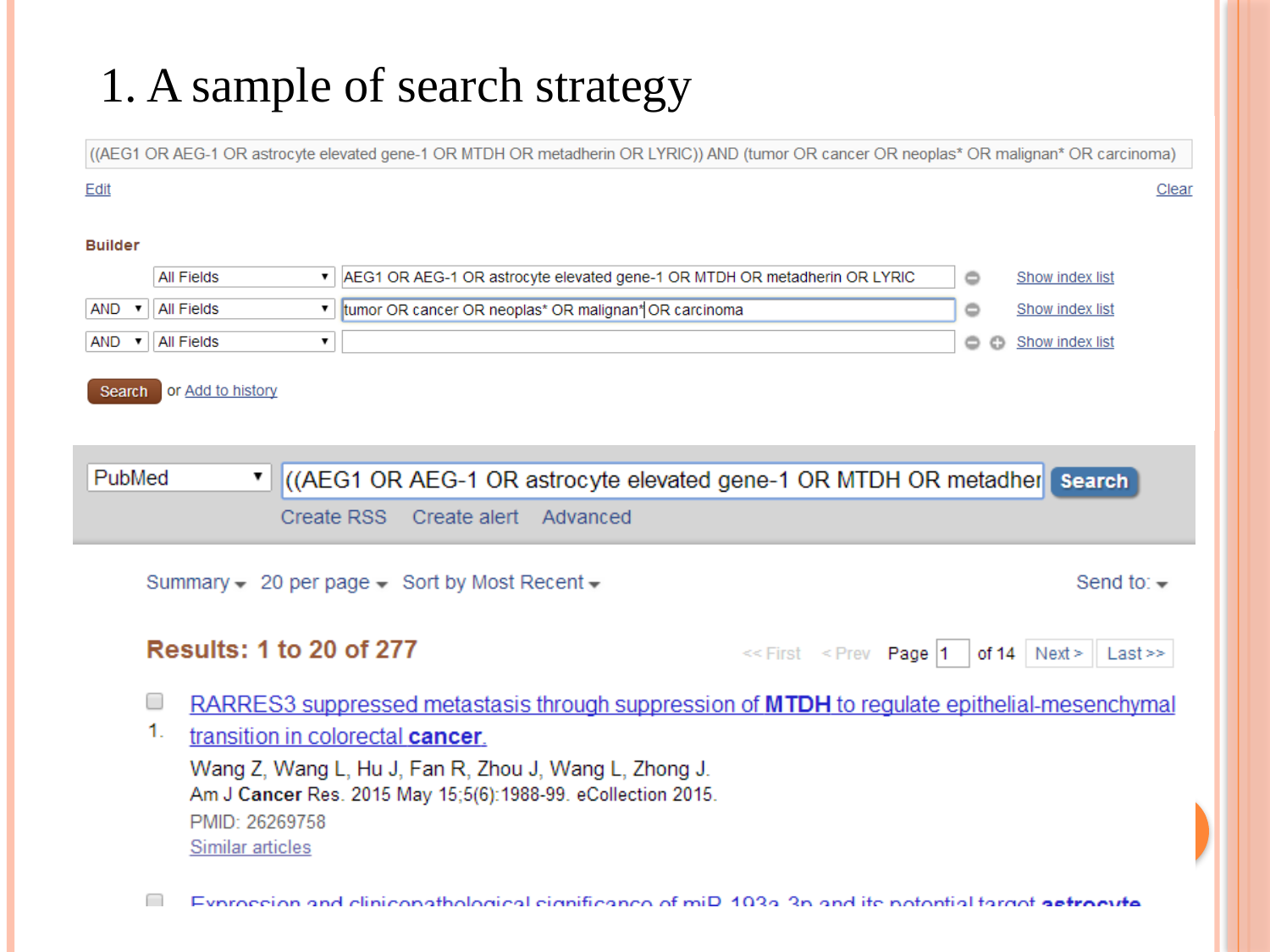

1. A sample of search strategy

## Slide 3
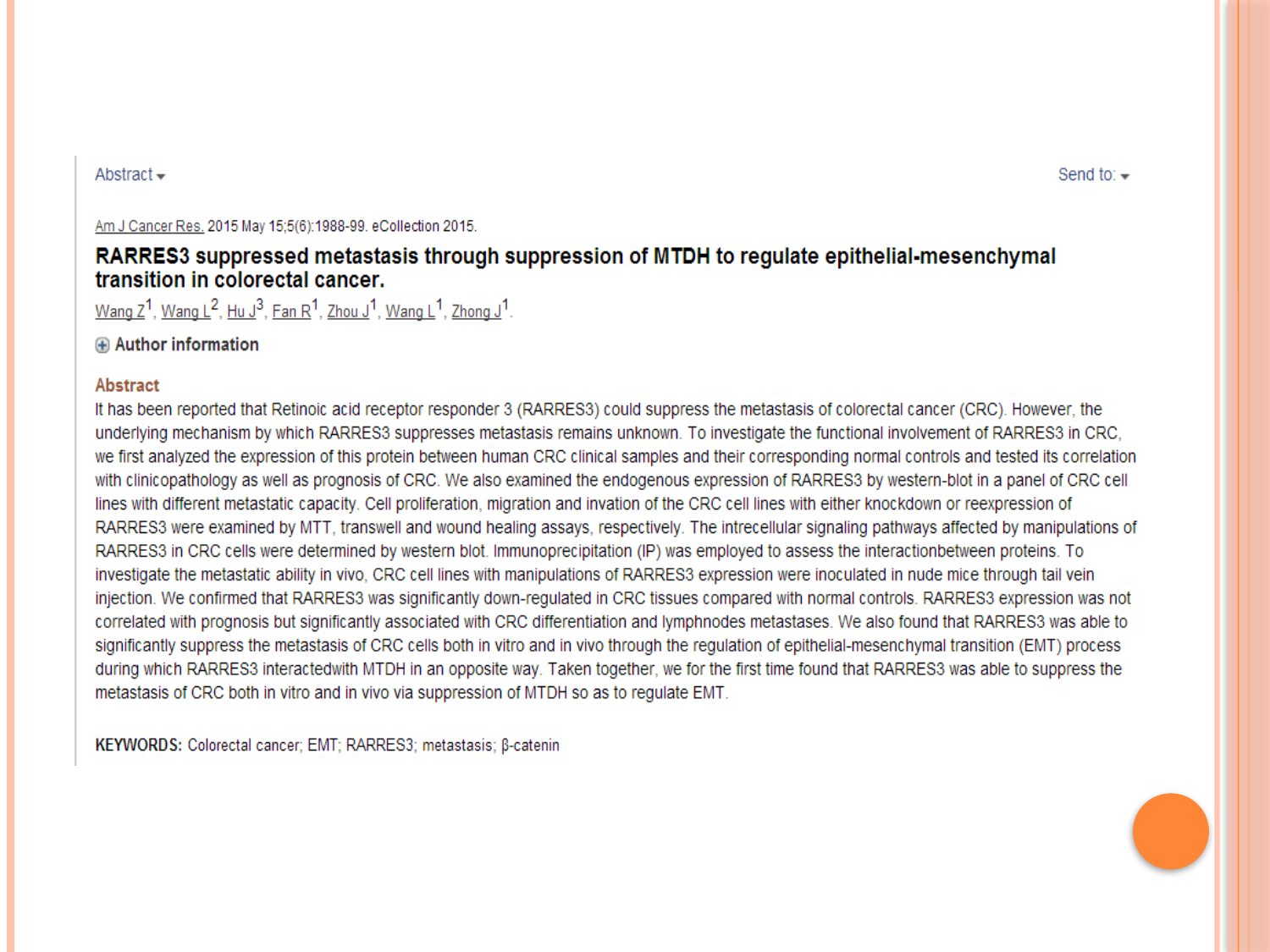

## Slide 4
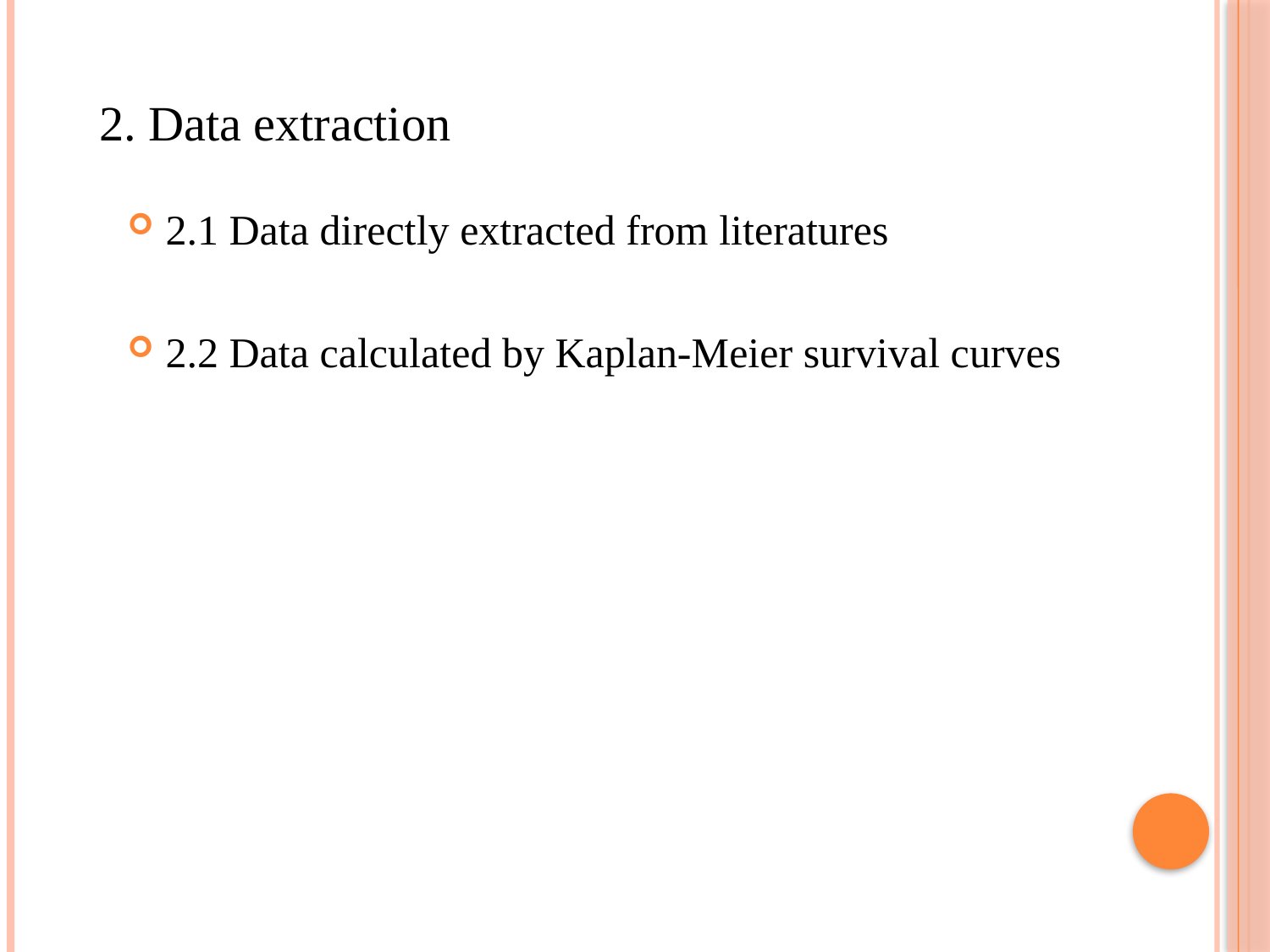

2. Data extraction
2.1 Data directly extracted from literatures
2.2 Data calculated by Kaplan-Meier survival curves

## Slide 5
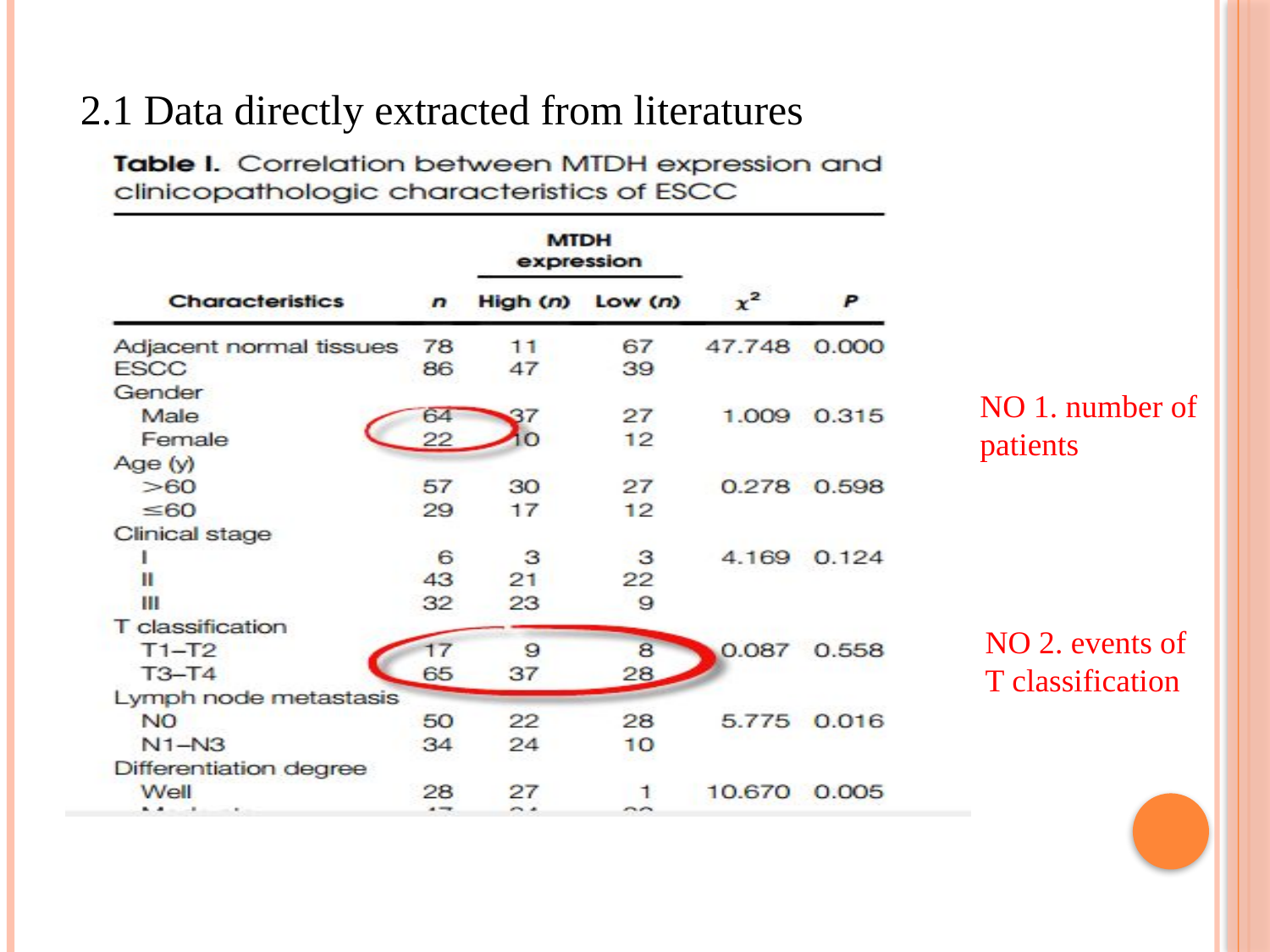

2.1 Data directly extracted from literatures
NO 1. number of
patients
NO 2. events of
T classification

## Slide 6
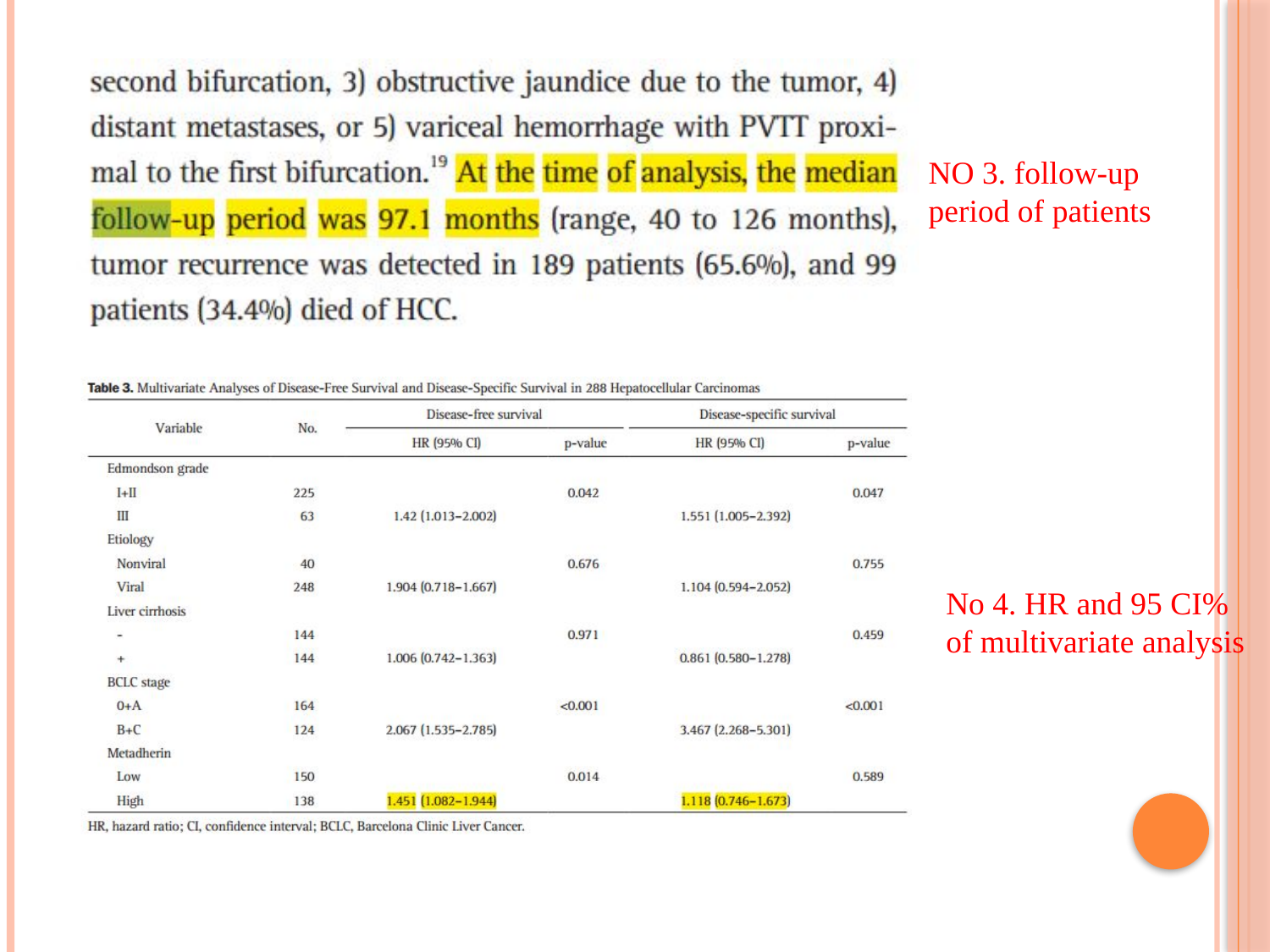

NO 3. follow-up
period of patients
No 4. HR and 95 CI%
of multivariate analysis

## Slide 7
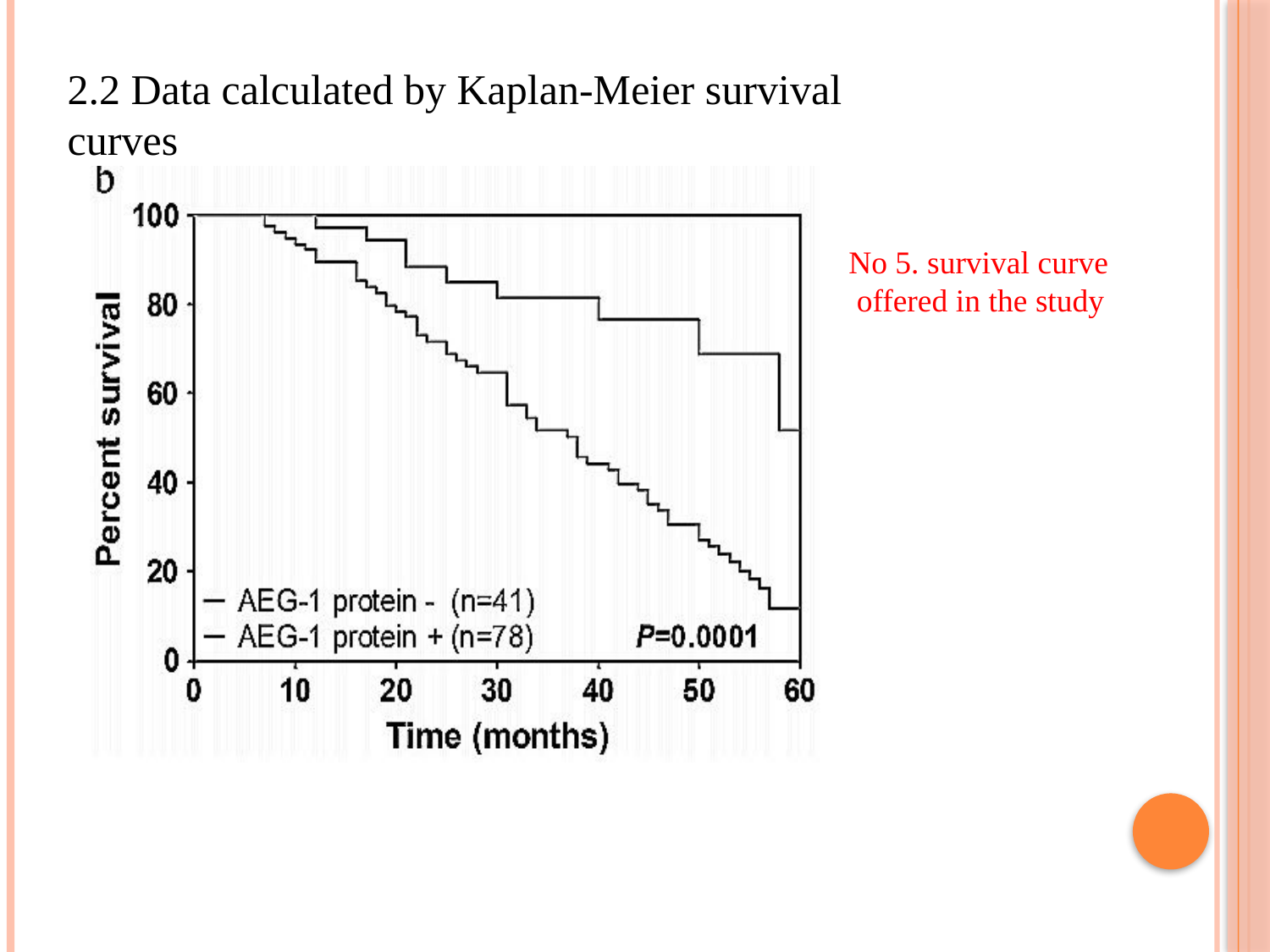

2.2 Data calculated by Kaplan-Meier survival curves
No 5. survival curve
 offered in the study

## Slide 8
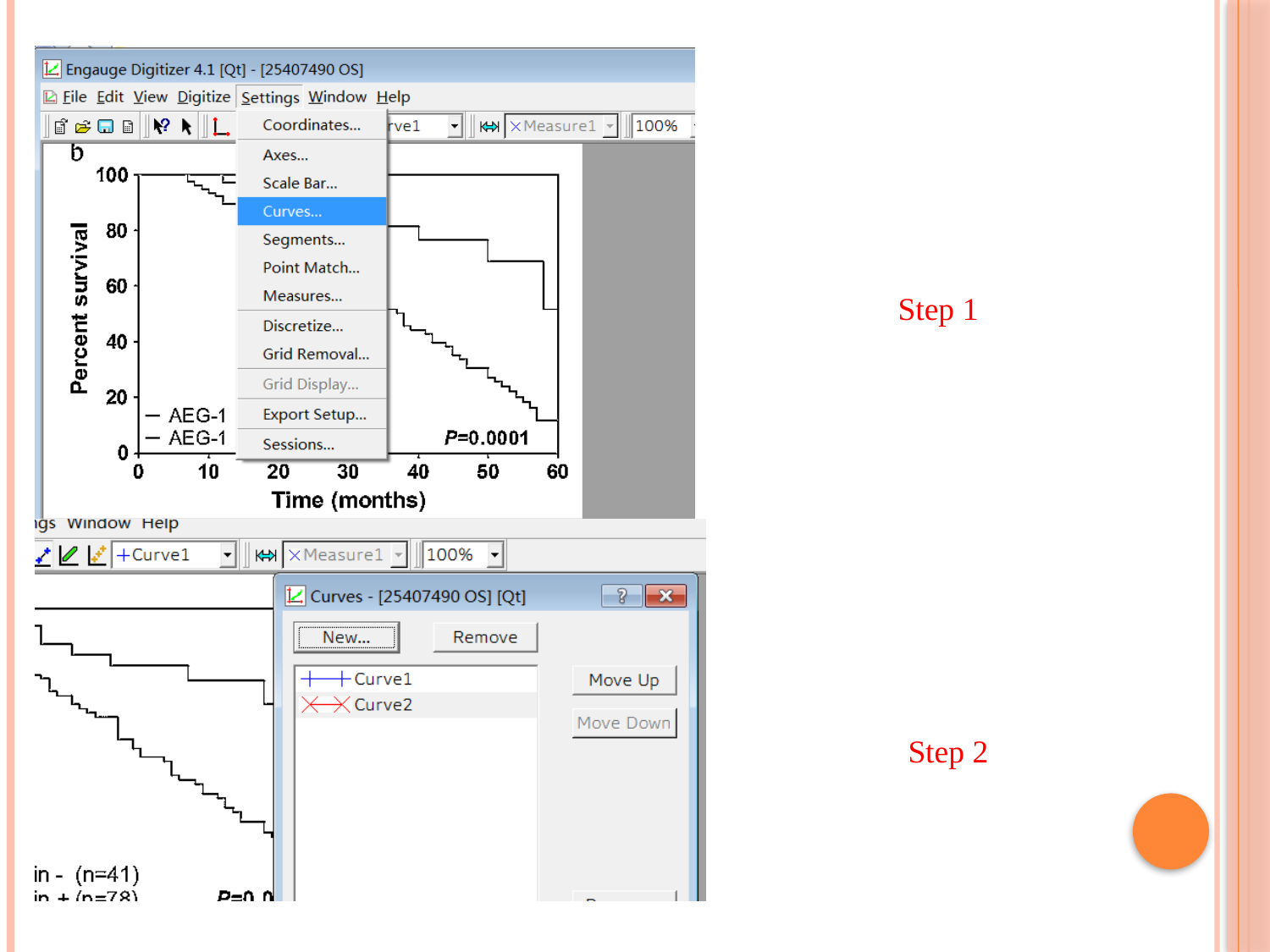

Step 1
Step 2

## Slide 9
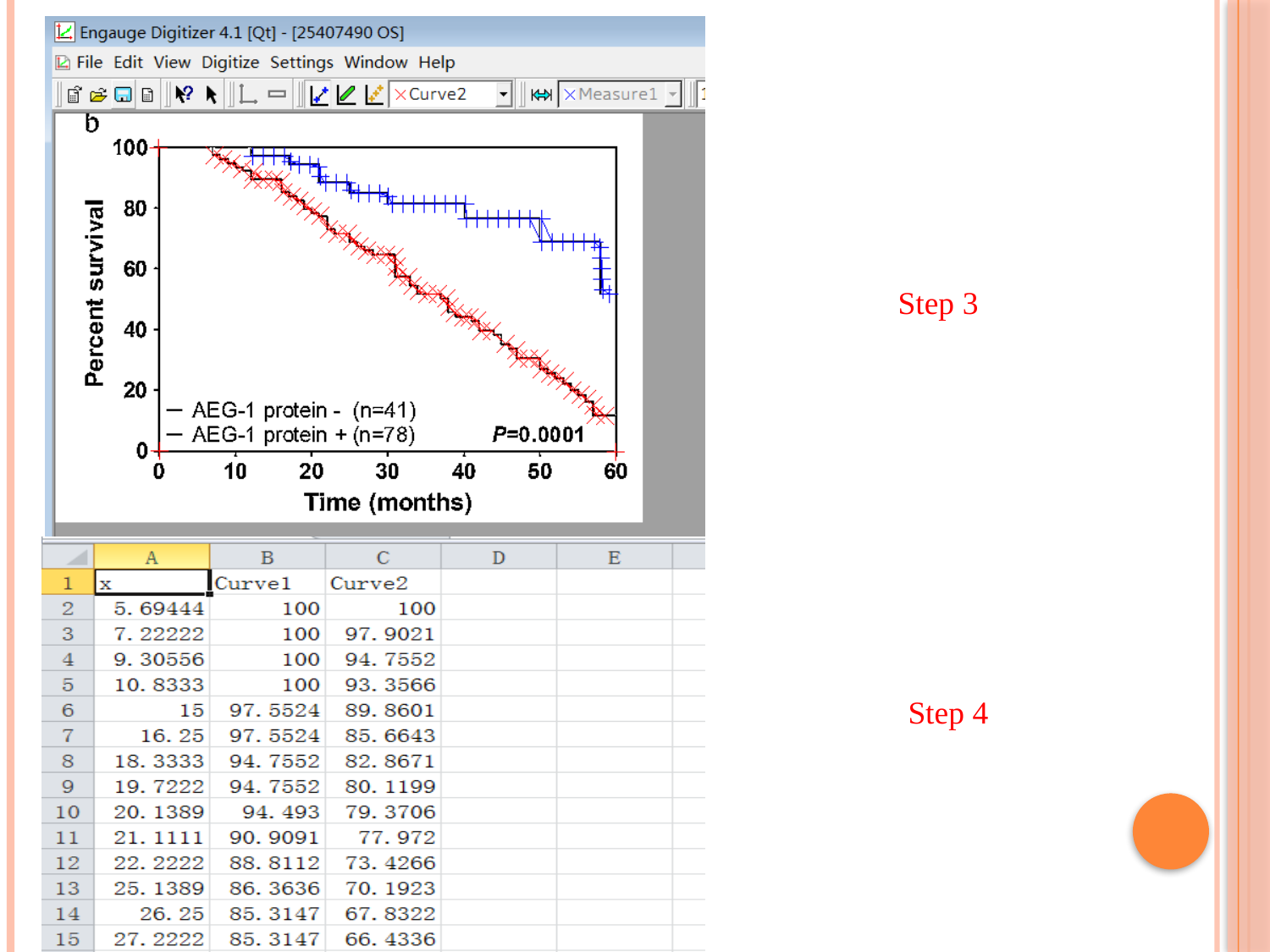

Step 3
Step 4

## Slide 10
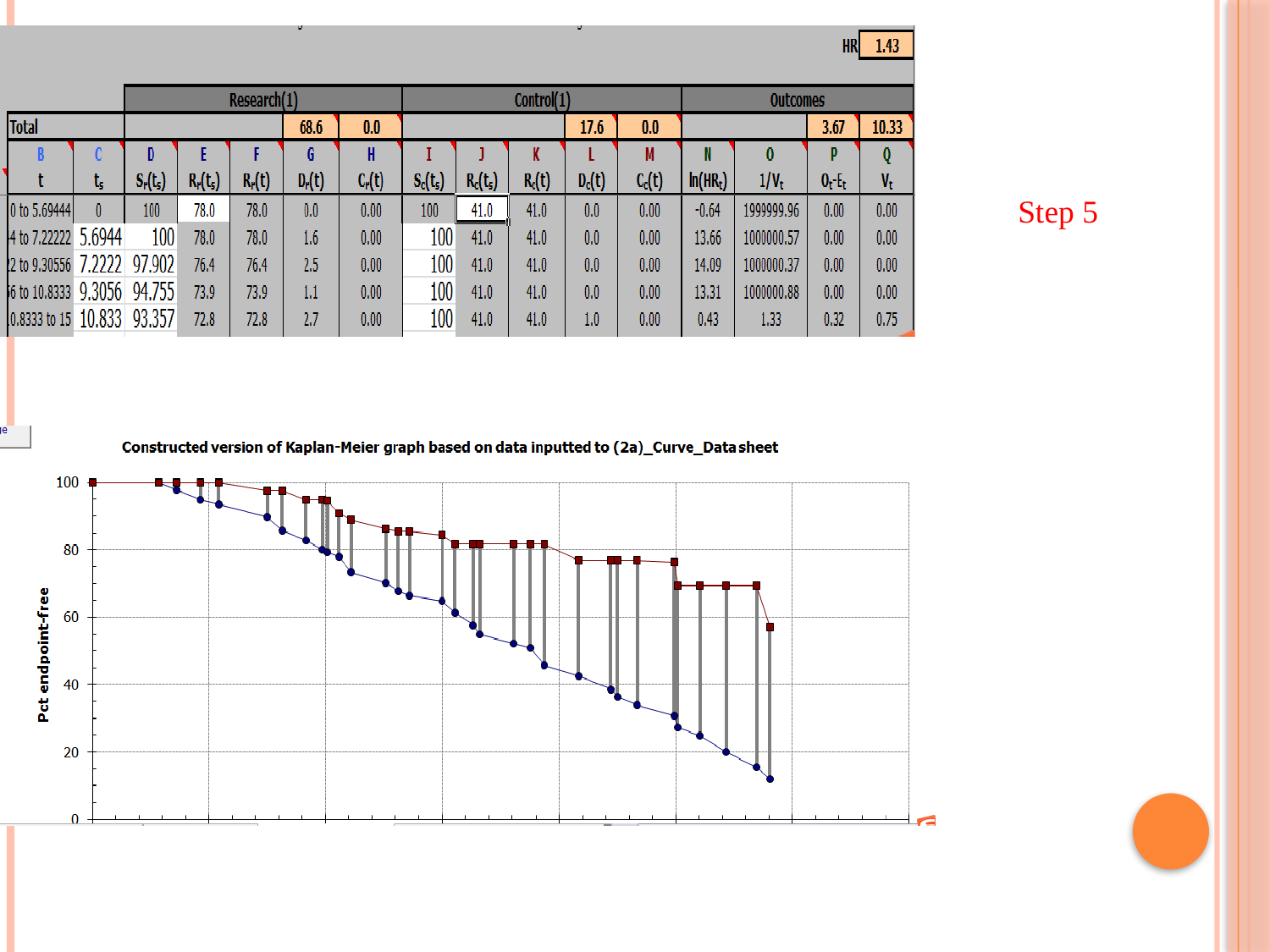

Step 5

## Slide 11
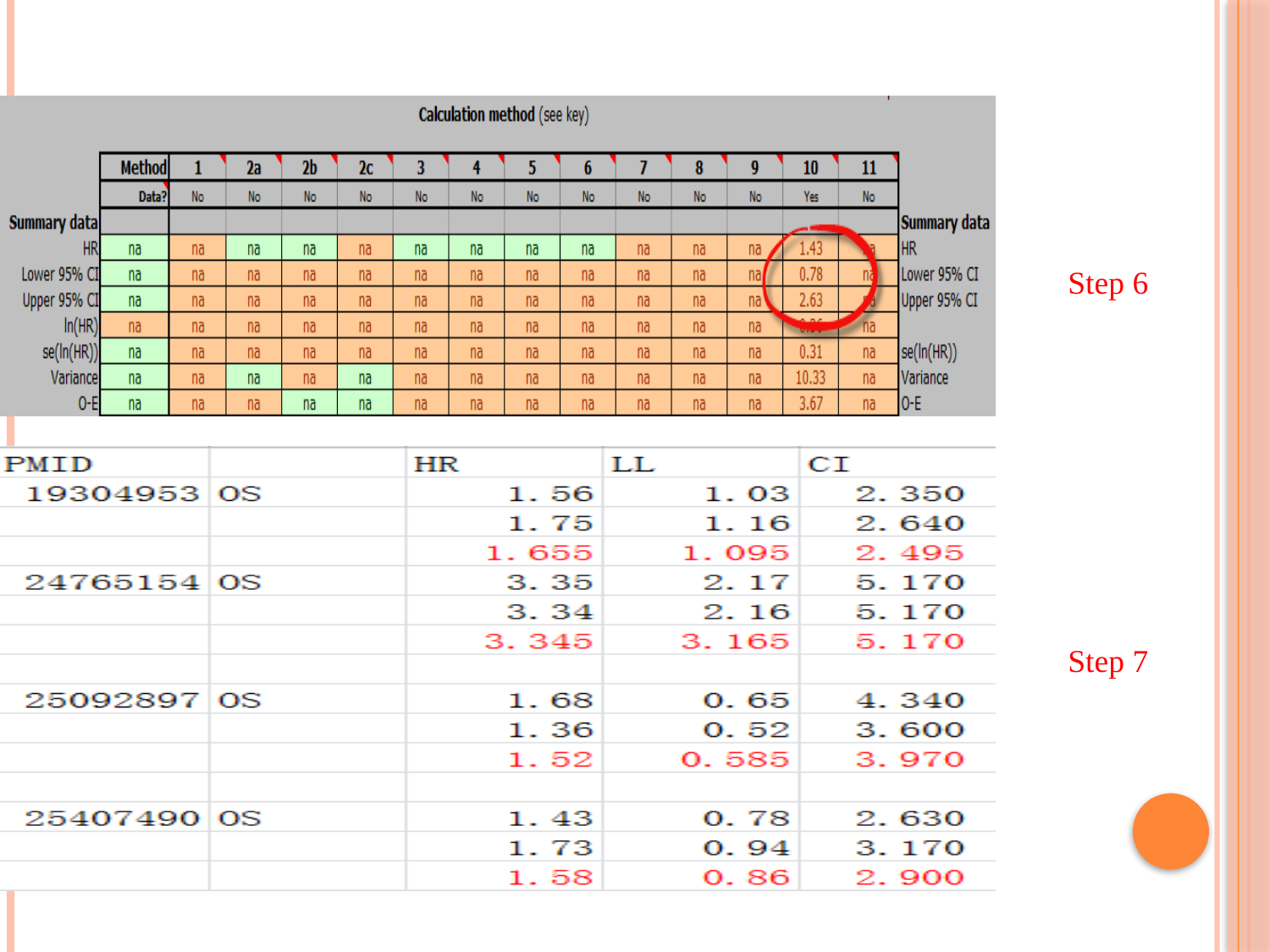

Step 6
Step 7

## Slide 12
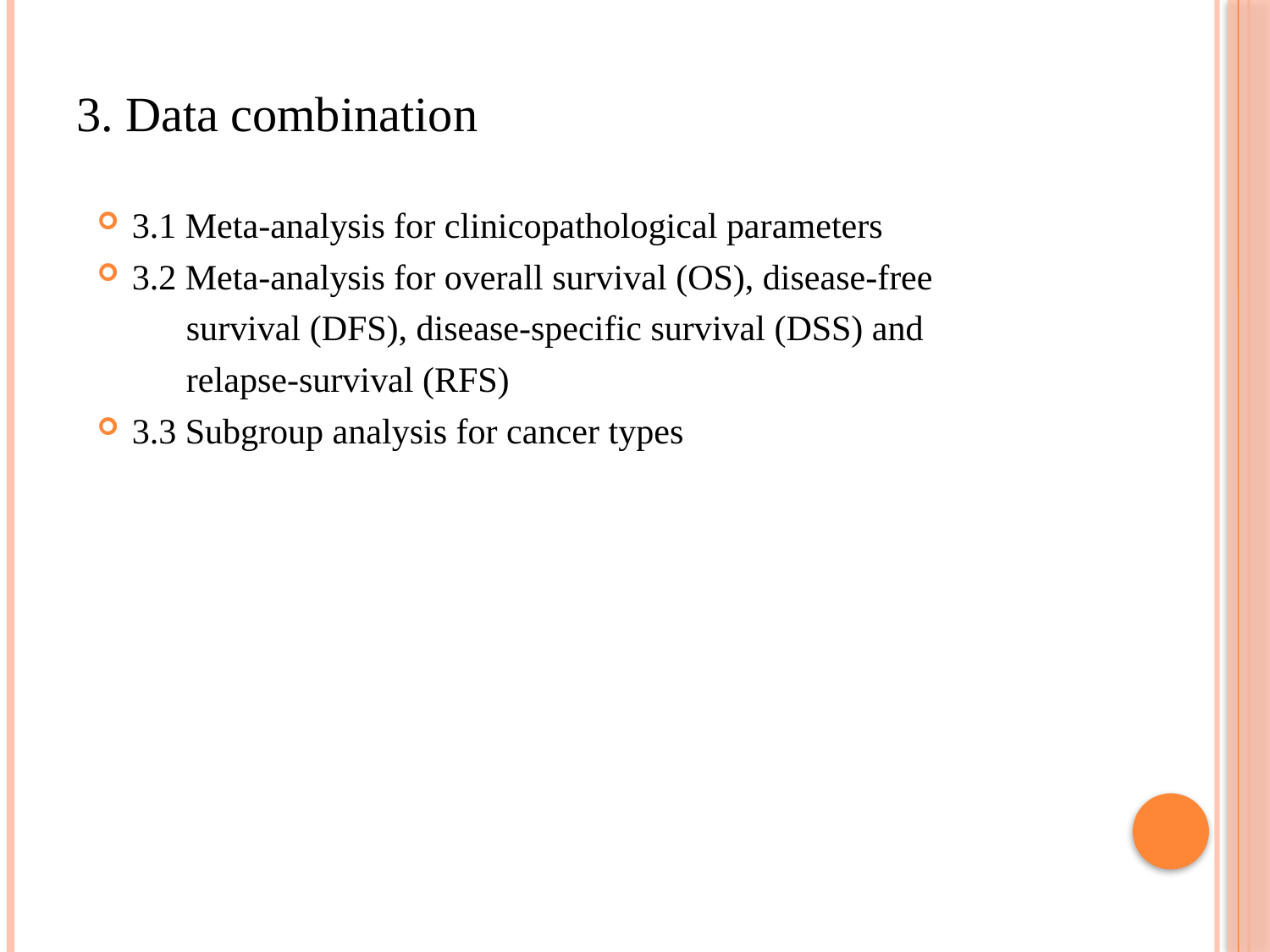

3. Data combination
3.1 Meta-analysis for clinicopathological parameters
3.2 Meta-analysis for overall survival (OS), disease-free
 survival (DFS), disease-specific survival (DSS) and
 relapse-survival (RFS)
3.3 Subgroup analysis for cancer types

## Slide 13
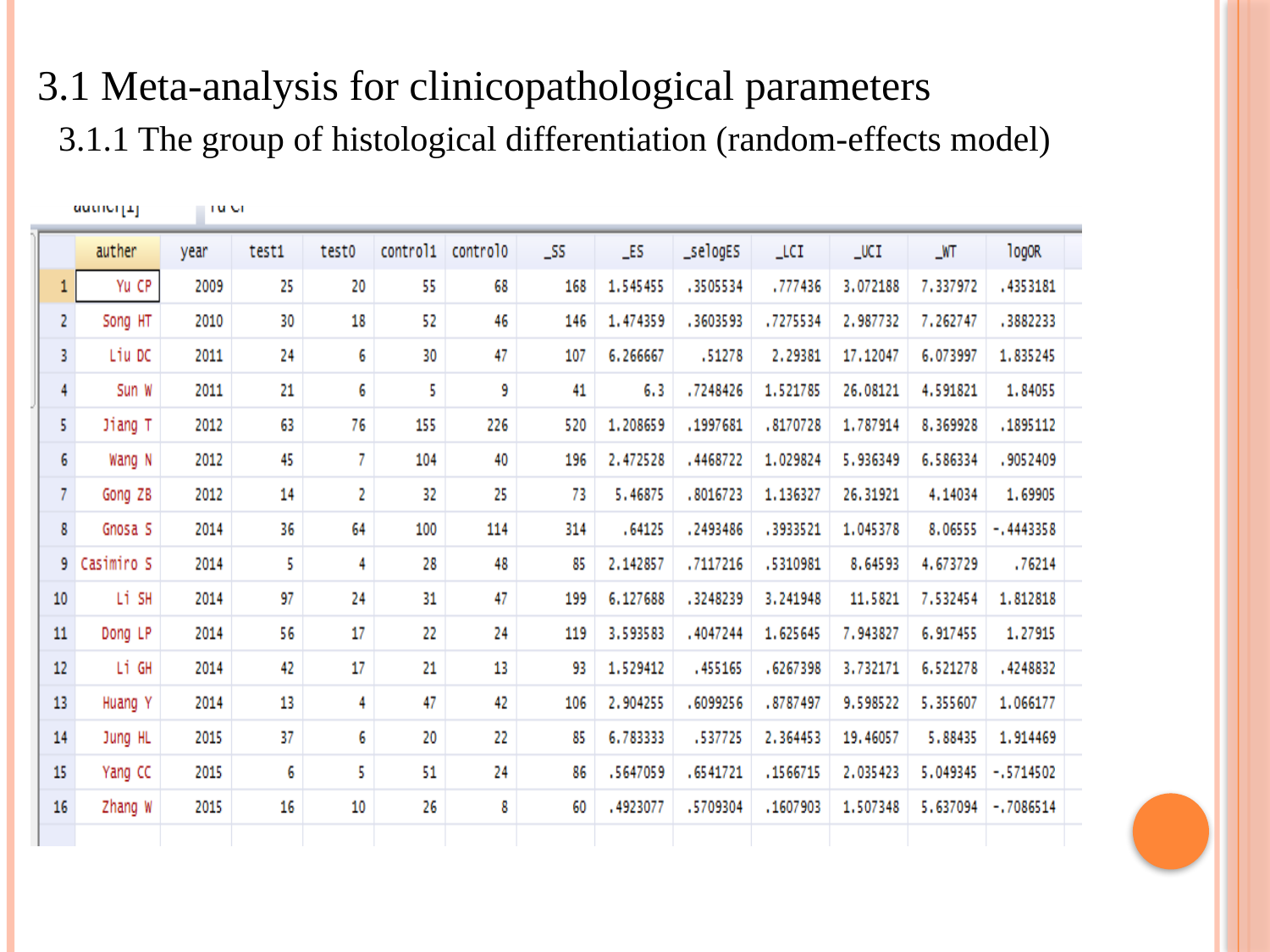

3.1 Meta-analysis for clinicopathological parameters
 3.1.1 The group of histological differentiation (random-effects model)

## Slide 14
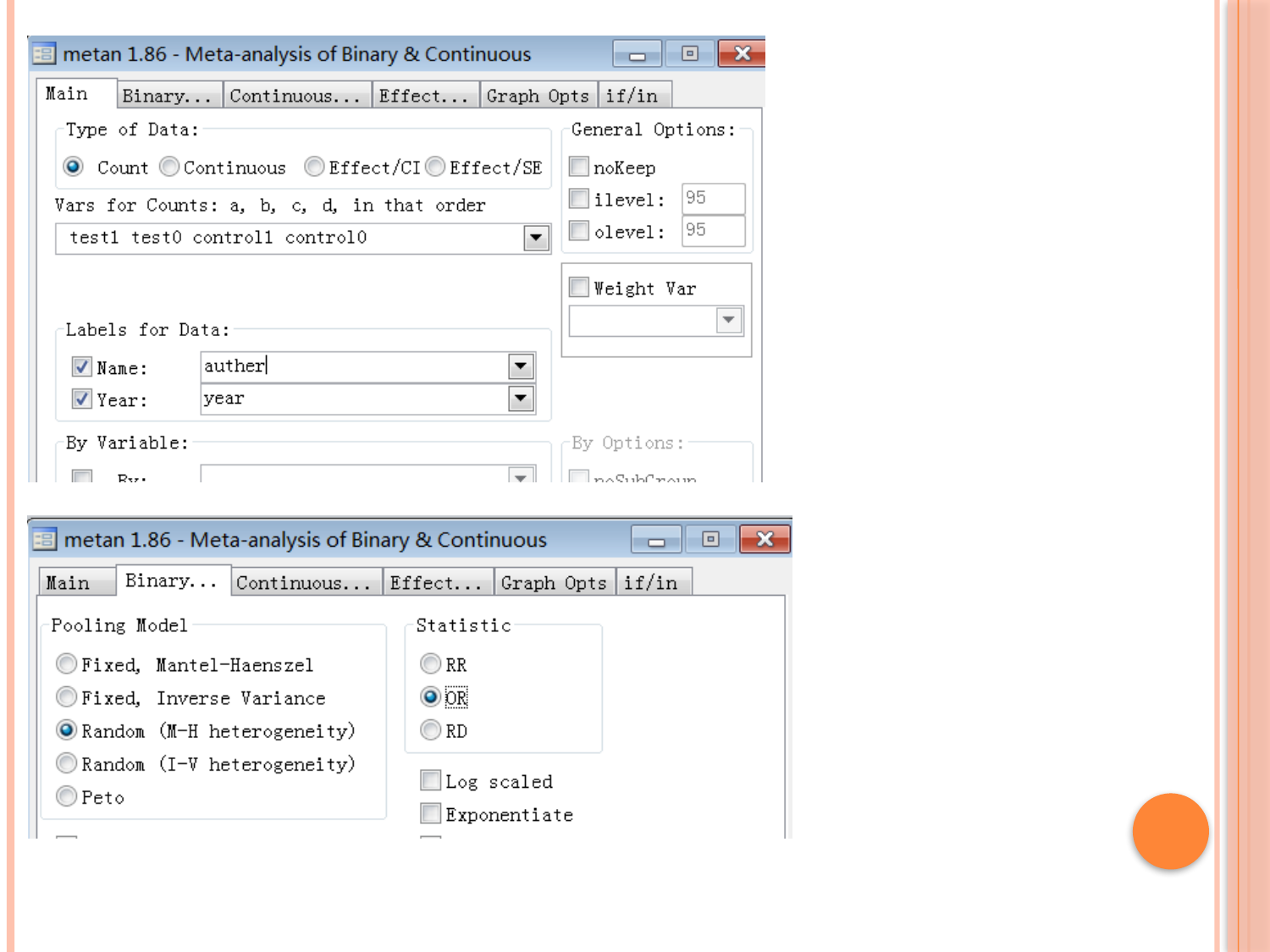

## Slide 15
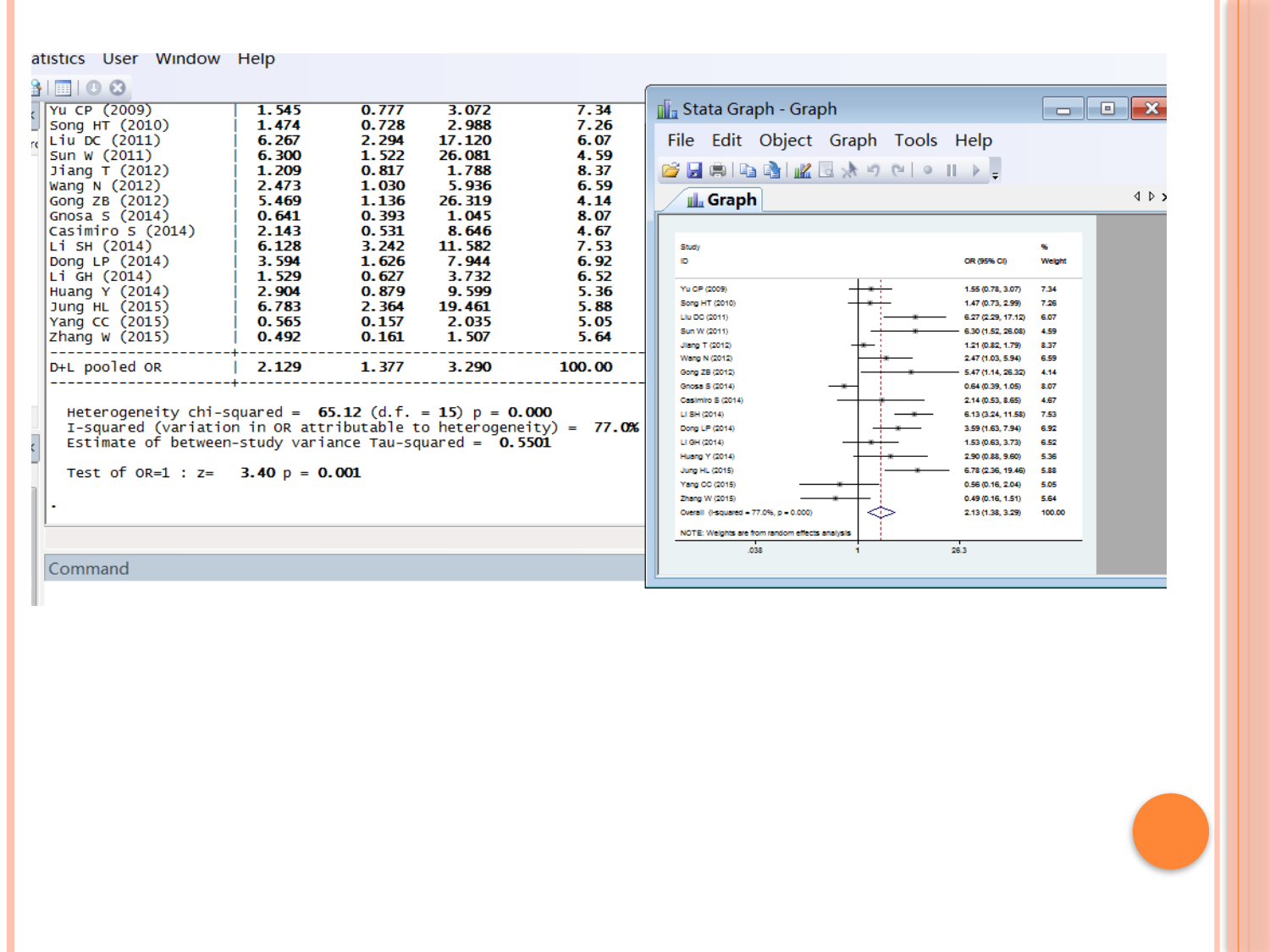

## Slide 16
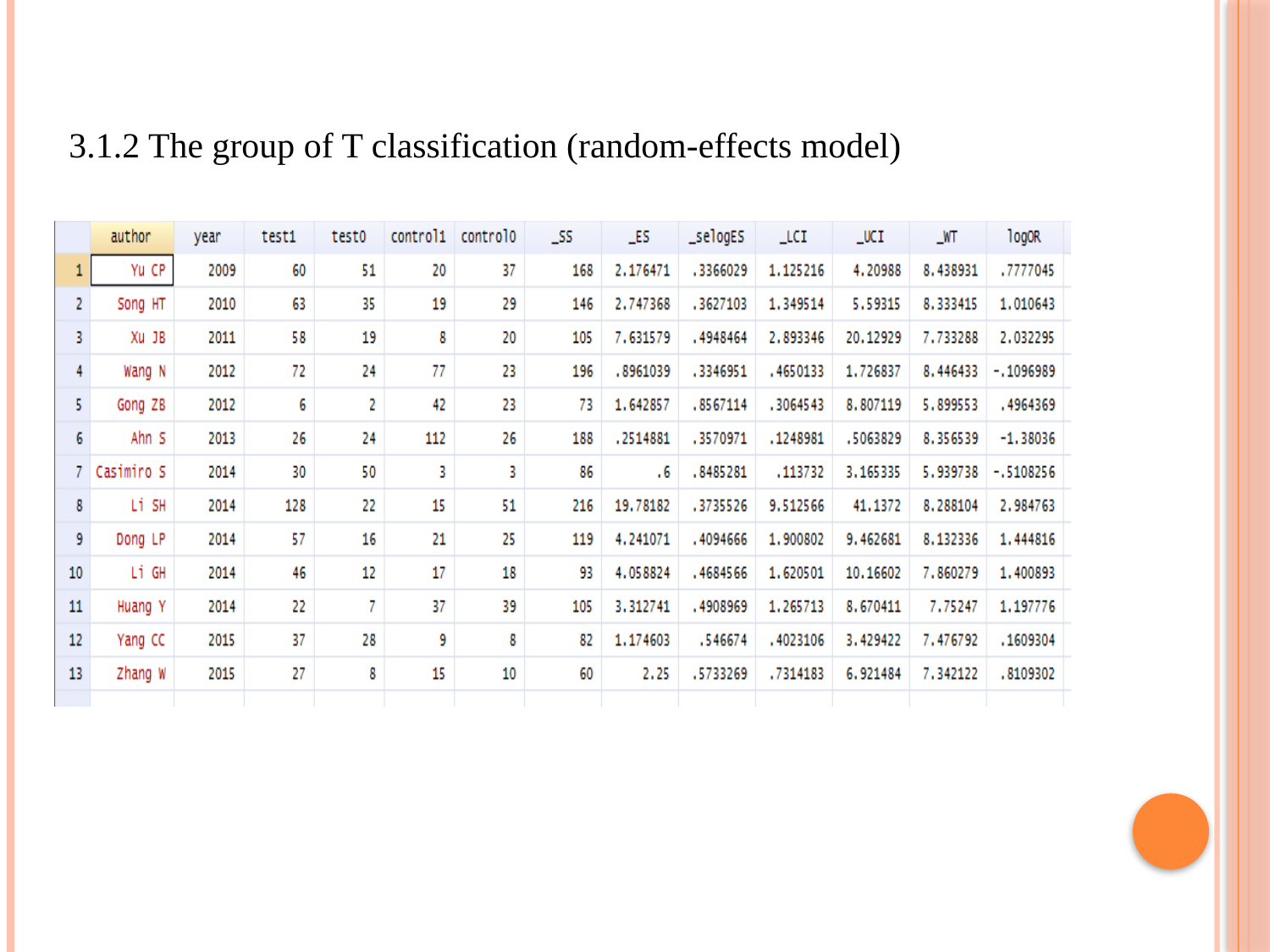

3.1.2 The group of T classification (random-effects model)

## Slide 17
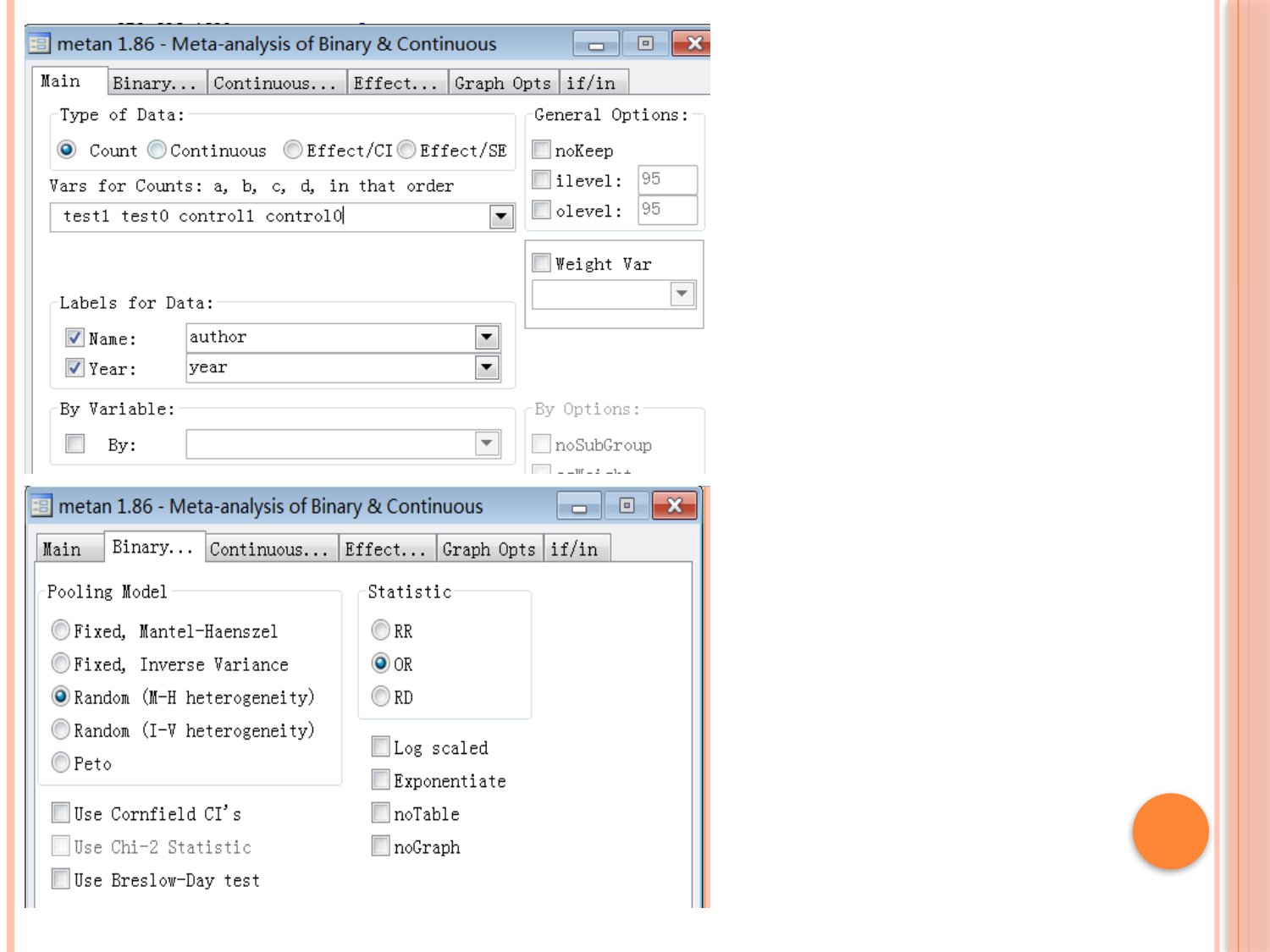

## Slide 18
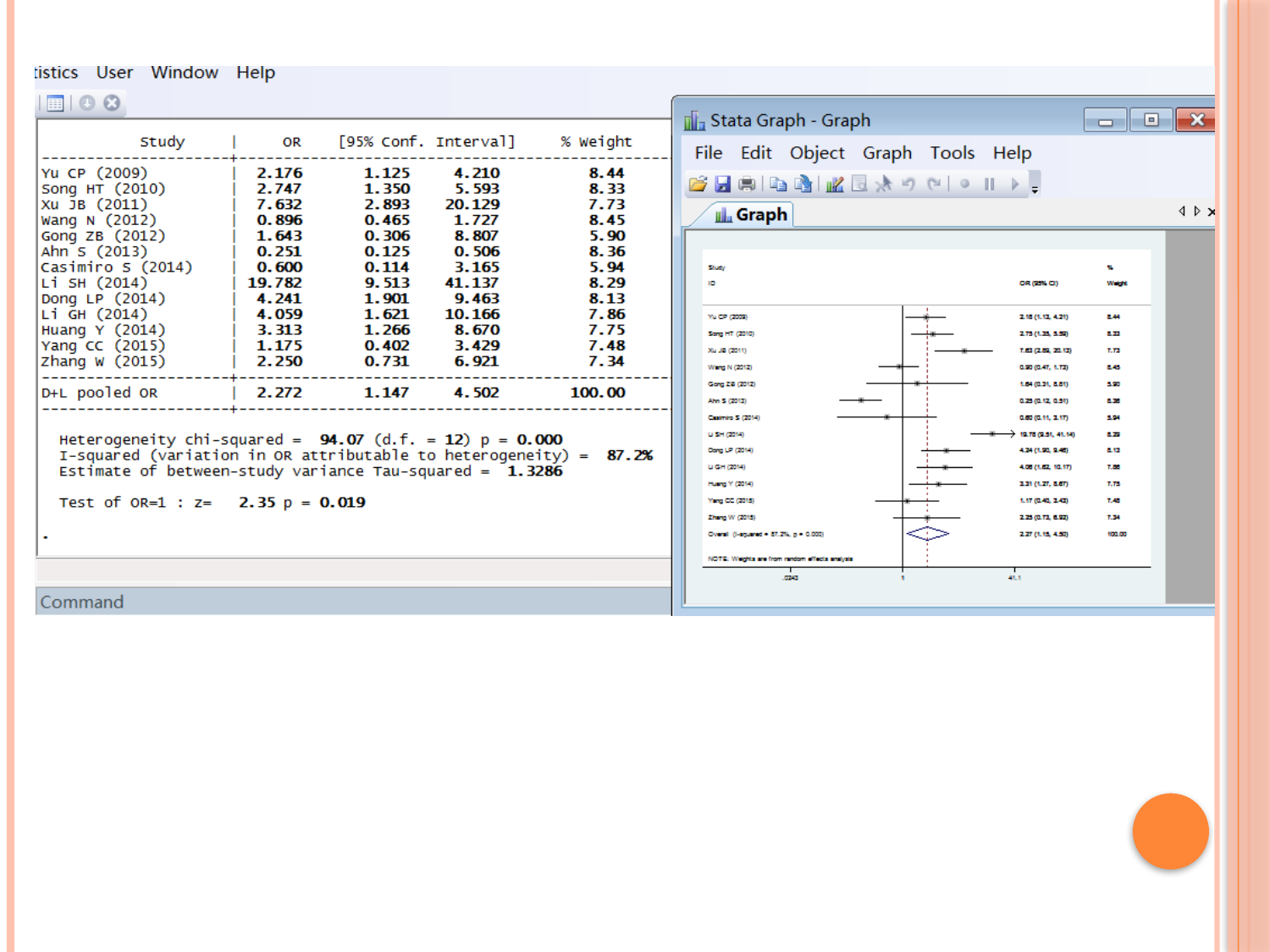

## Slide 19
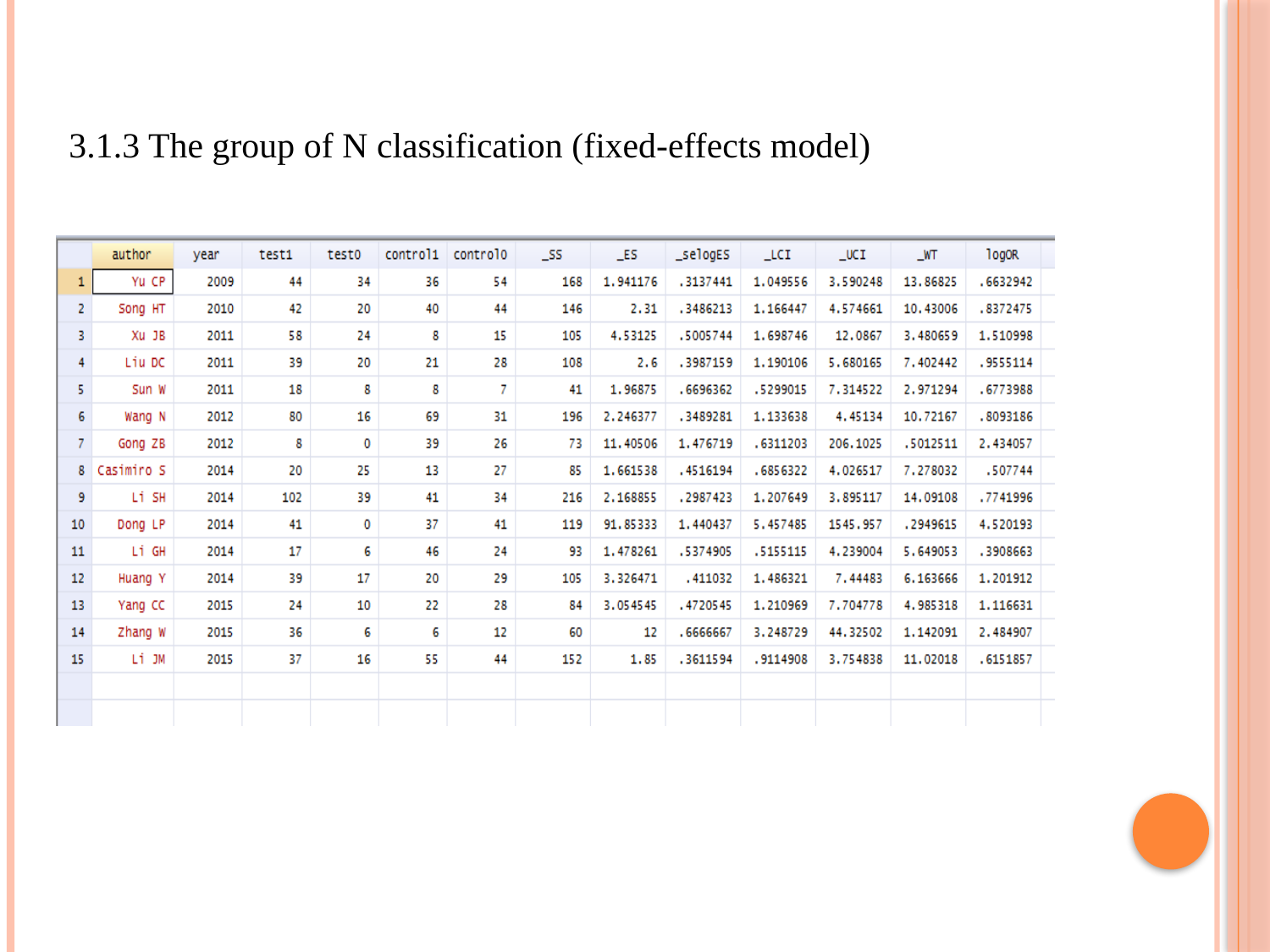

3.1.3 The group of N classification (fixed-effects model)

## Slide 20
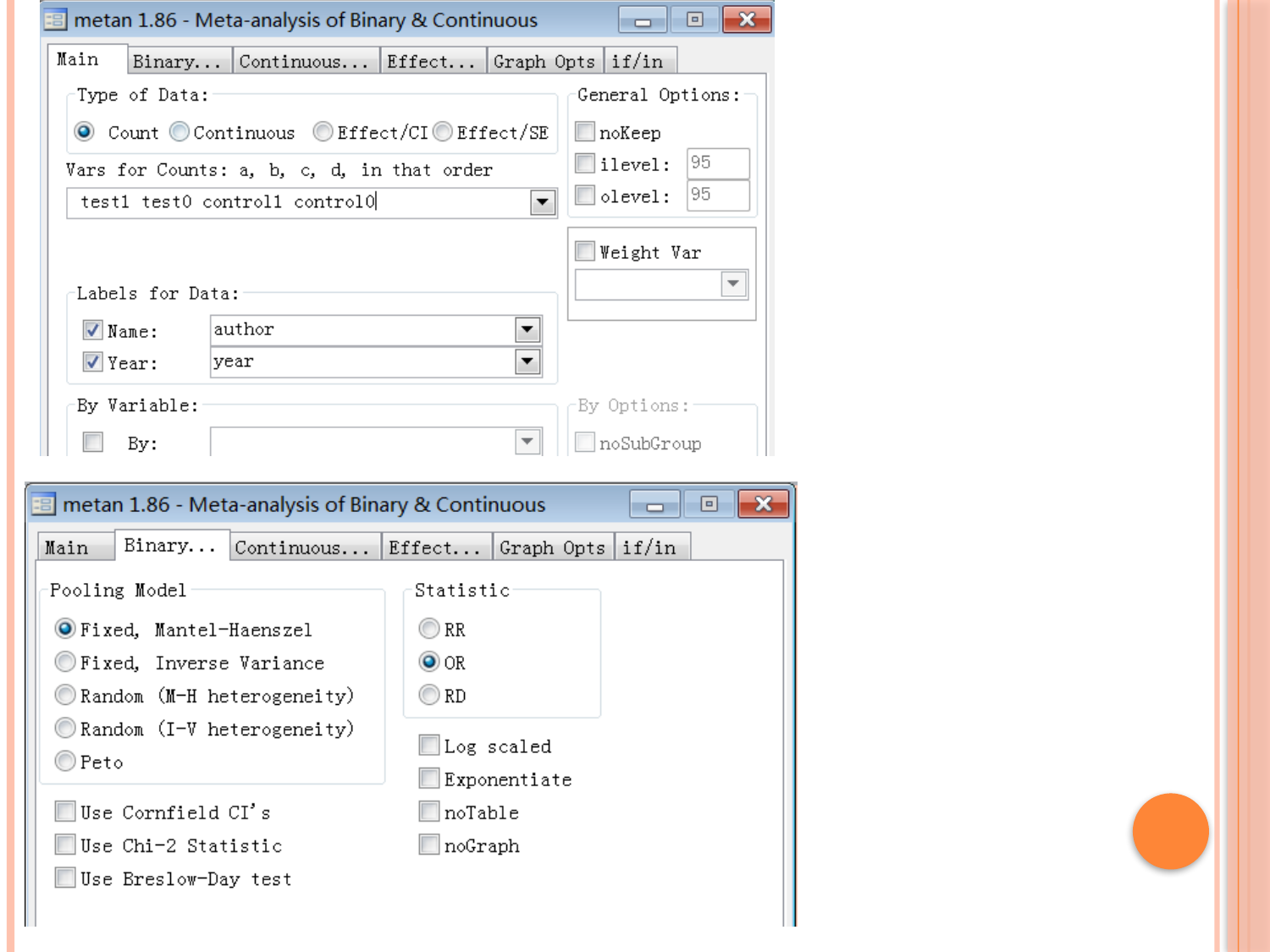

## Slide 21
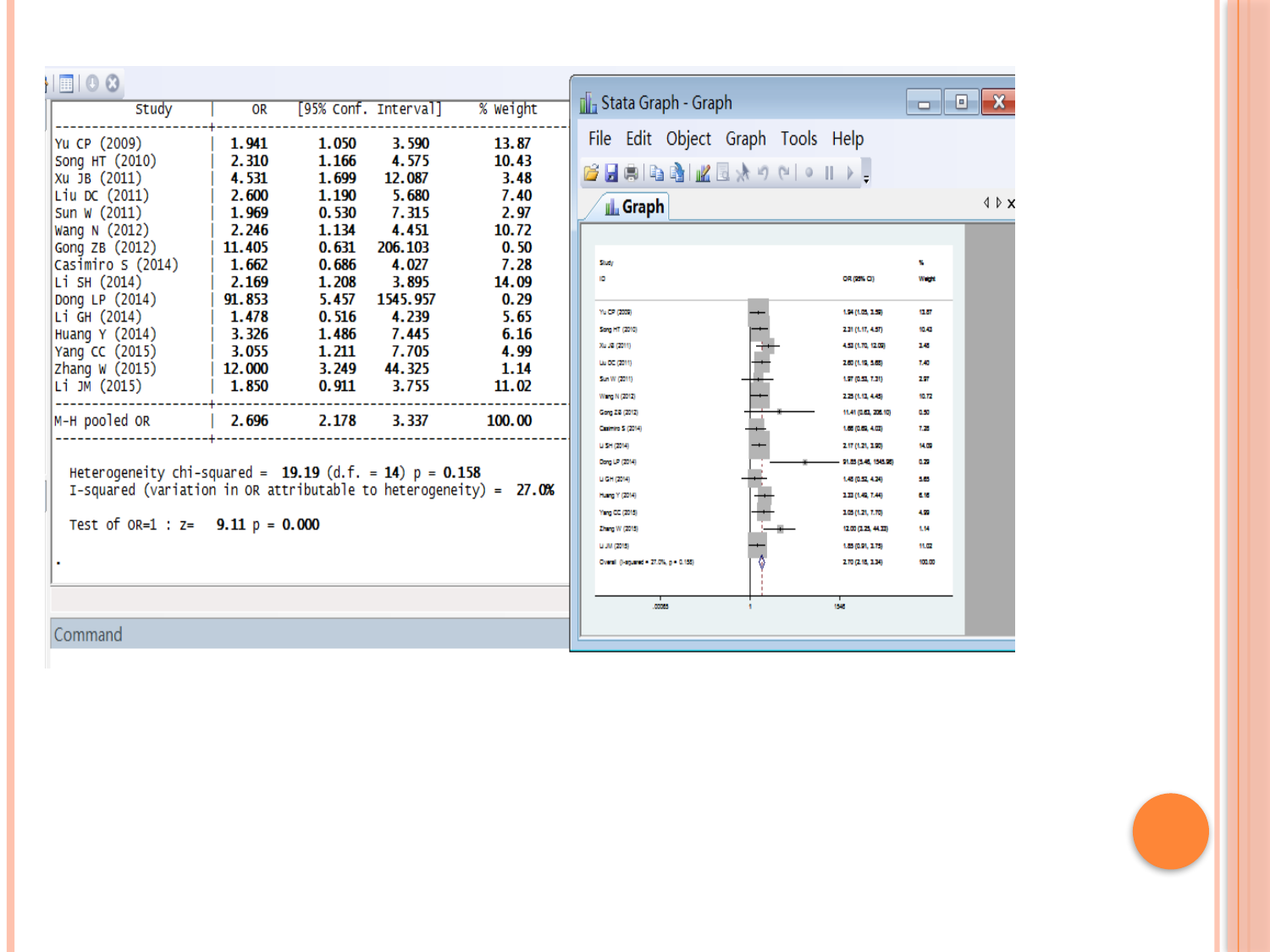

## Slide 22
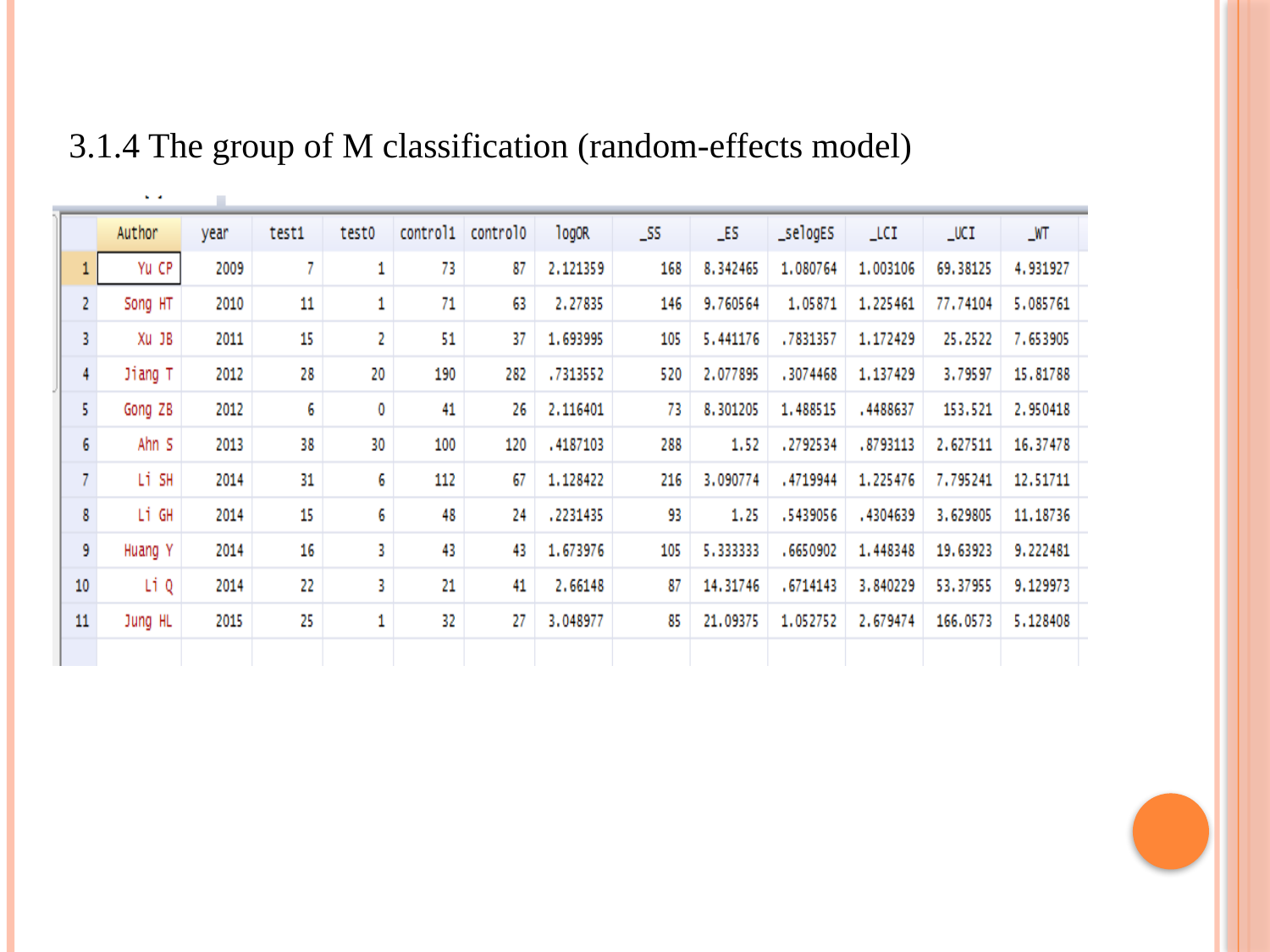

3.1.4 The group of M classification (random-effects model)

## Slide 23
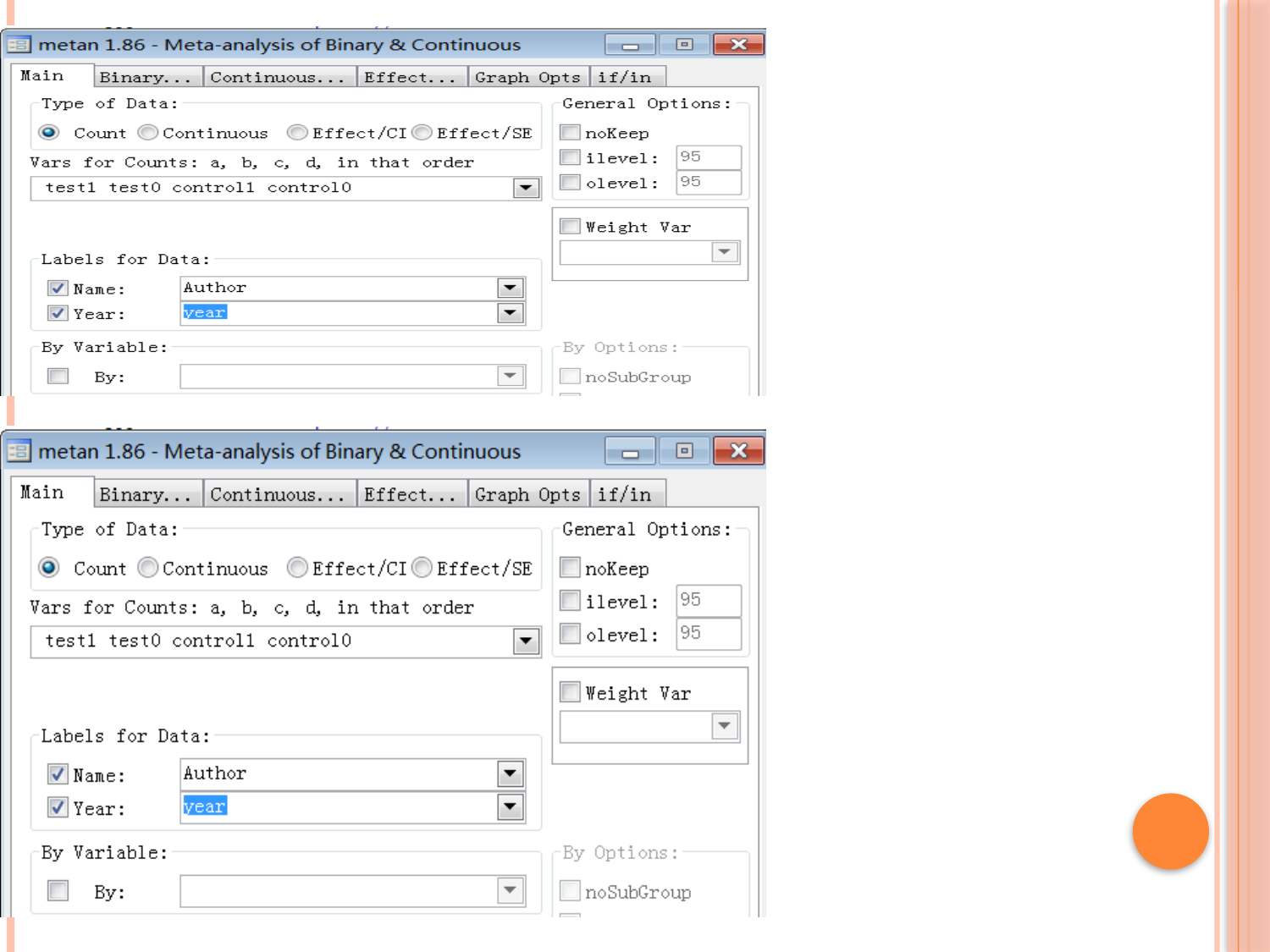

## Slide 24
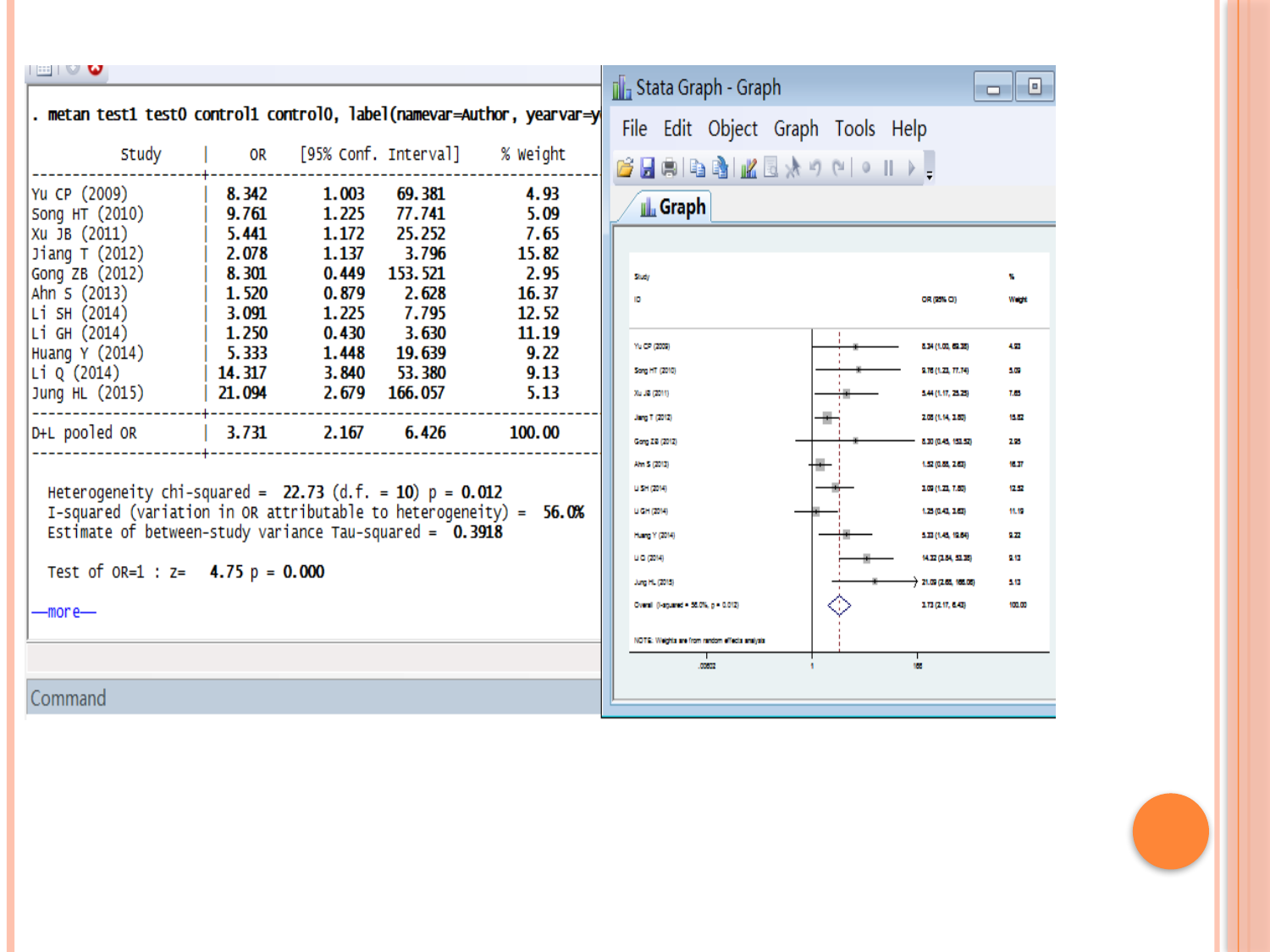

## Slide 25
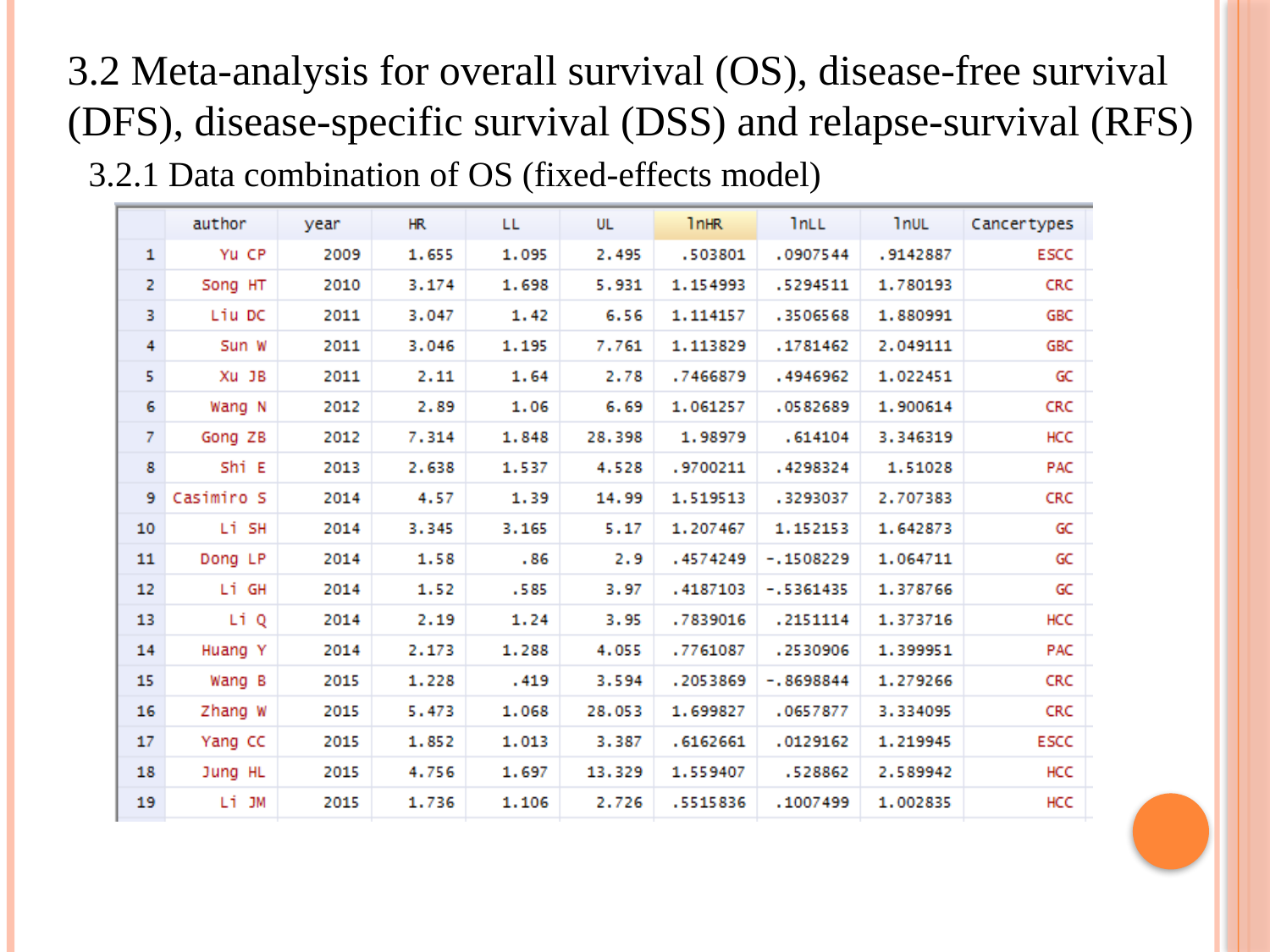

3.2 Meta-analysis for overall survival (OS), disease-free survival (DFS), disease-specific survival (DSS) and relapse-survival (RFS)
 3.2.1 Data combination of OS (fixed-effects model)

## Slide 26
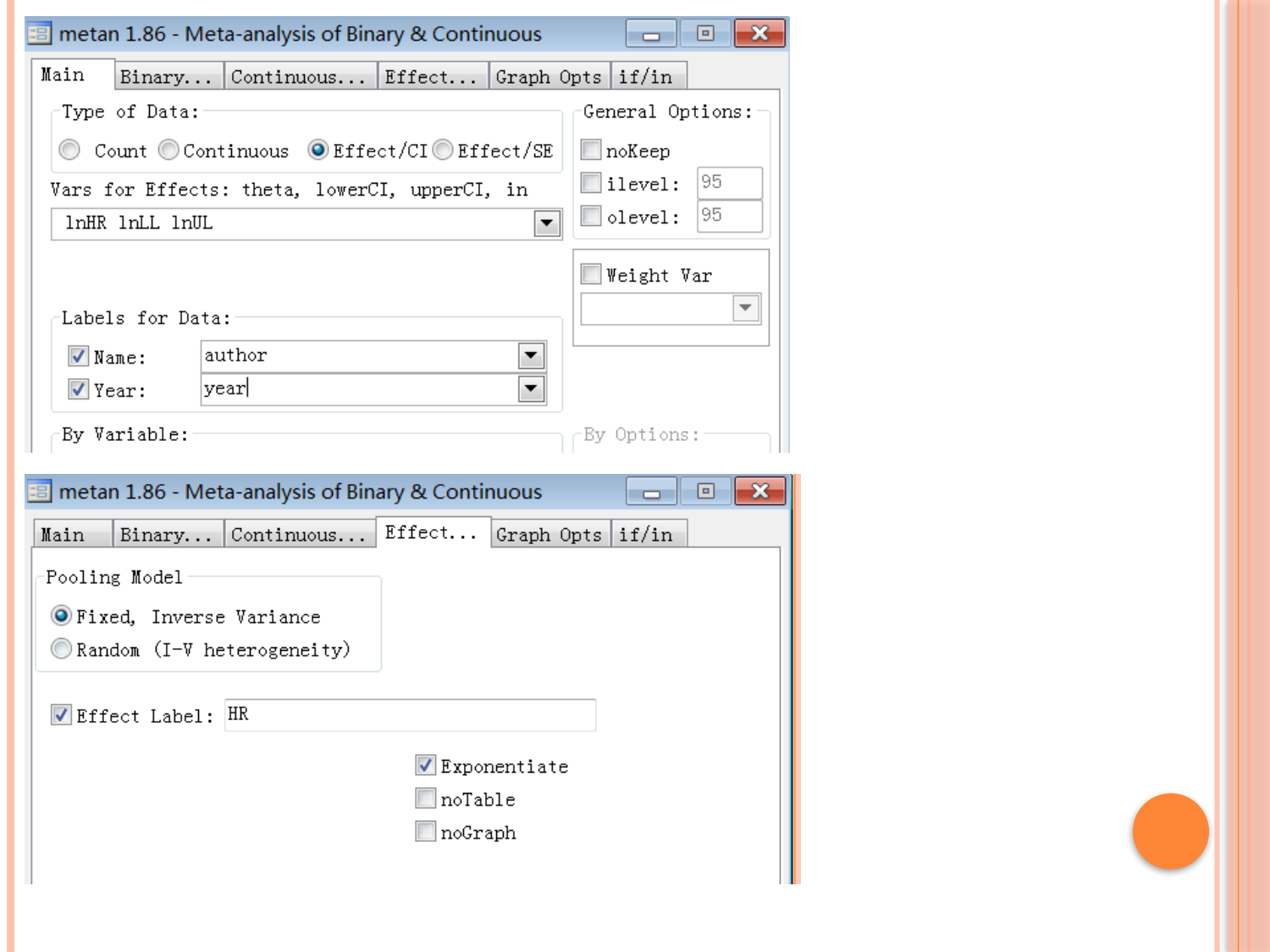

## Slide 27
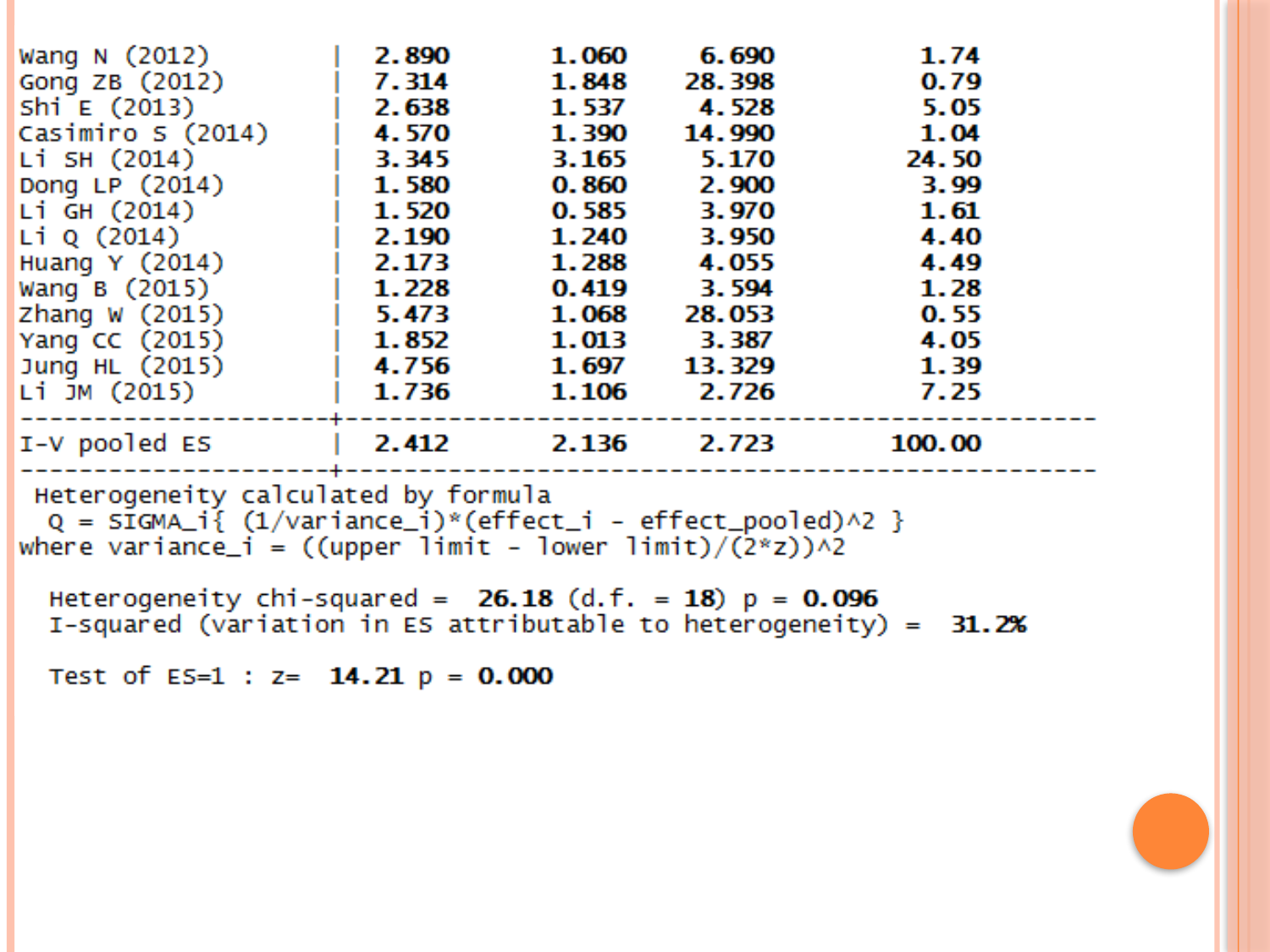

## Slide 28
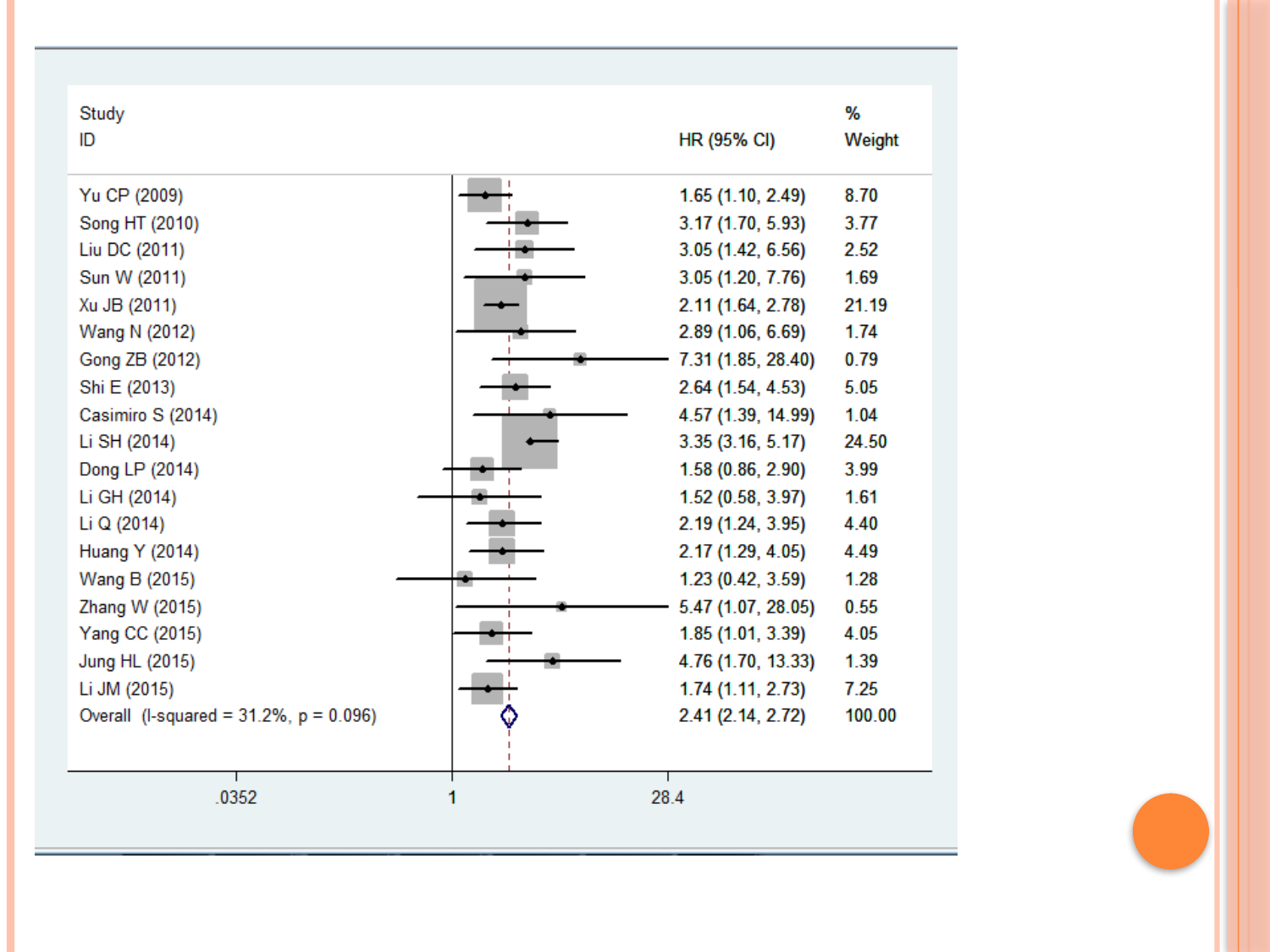

## Slide 29
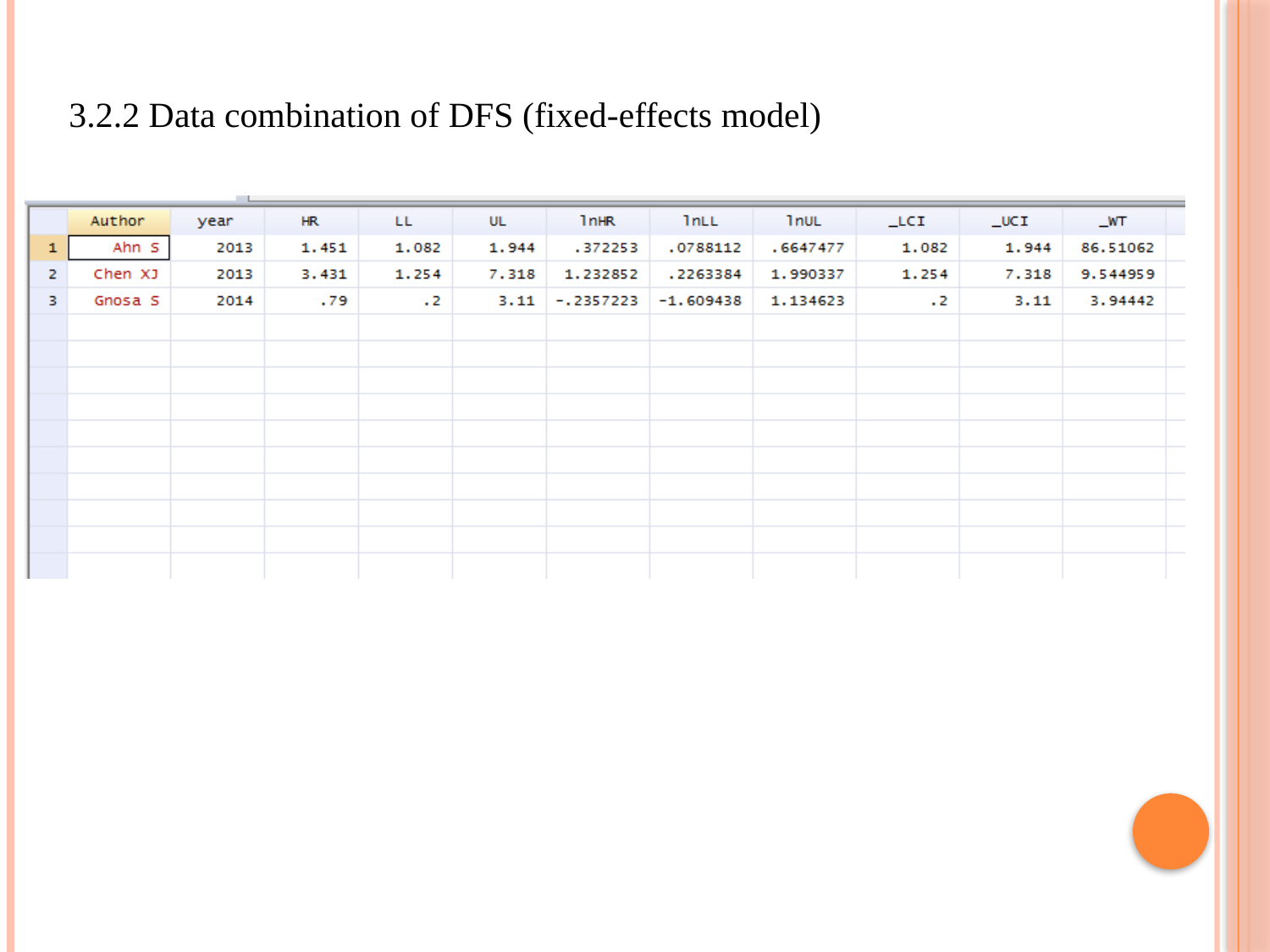

3.2.2 Data combination of DFS (fixed-effects model)

## Slide 30
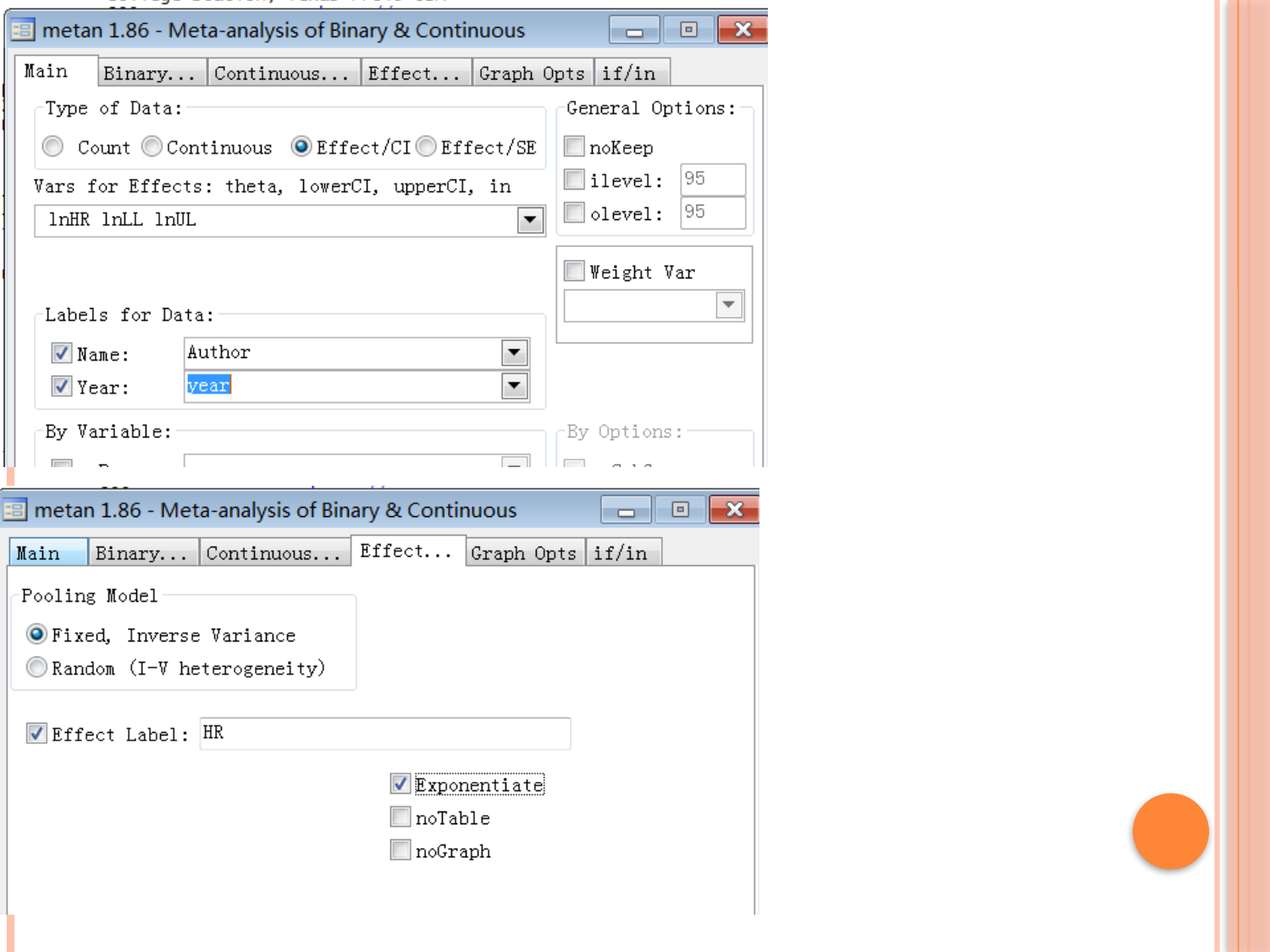

## Slide 31
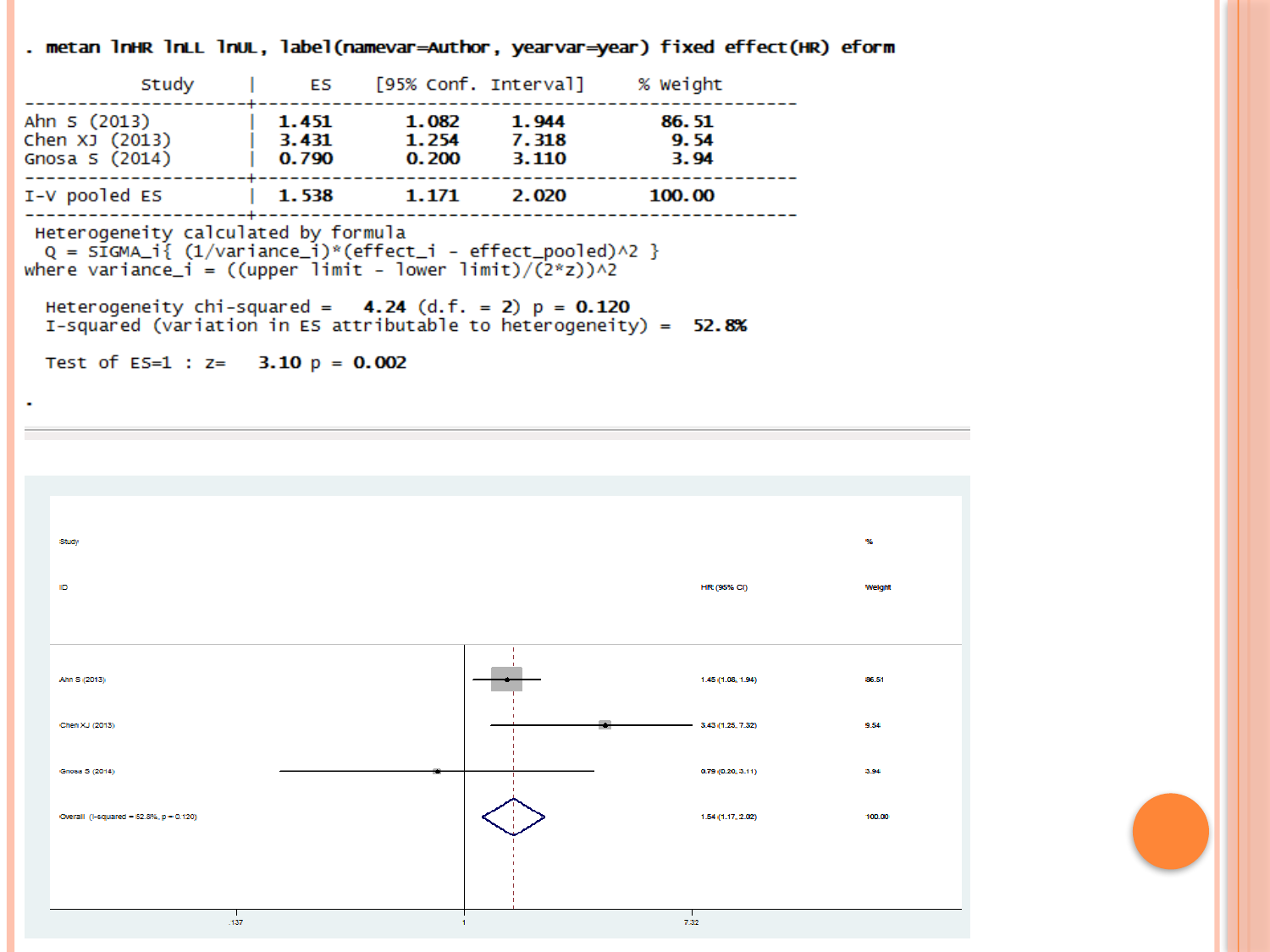

## Slide 32
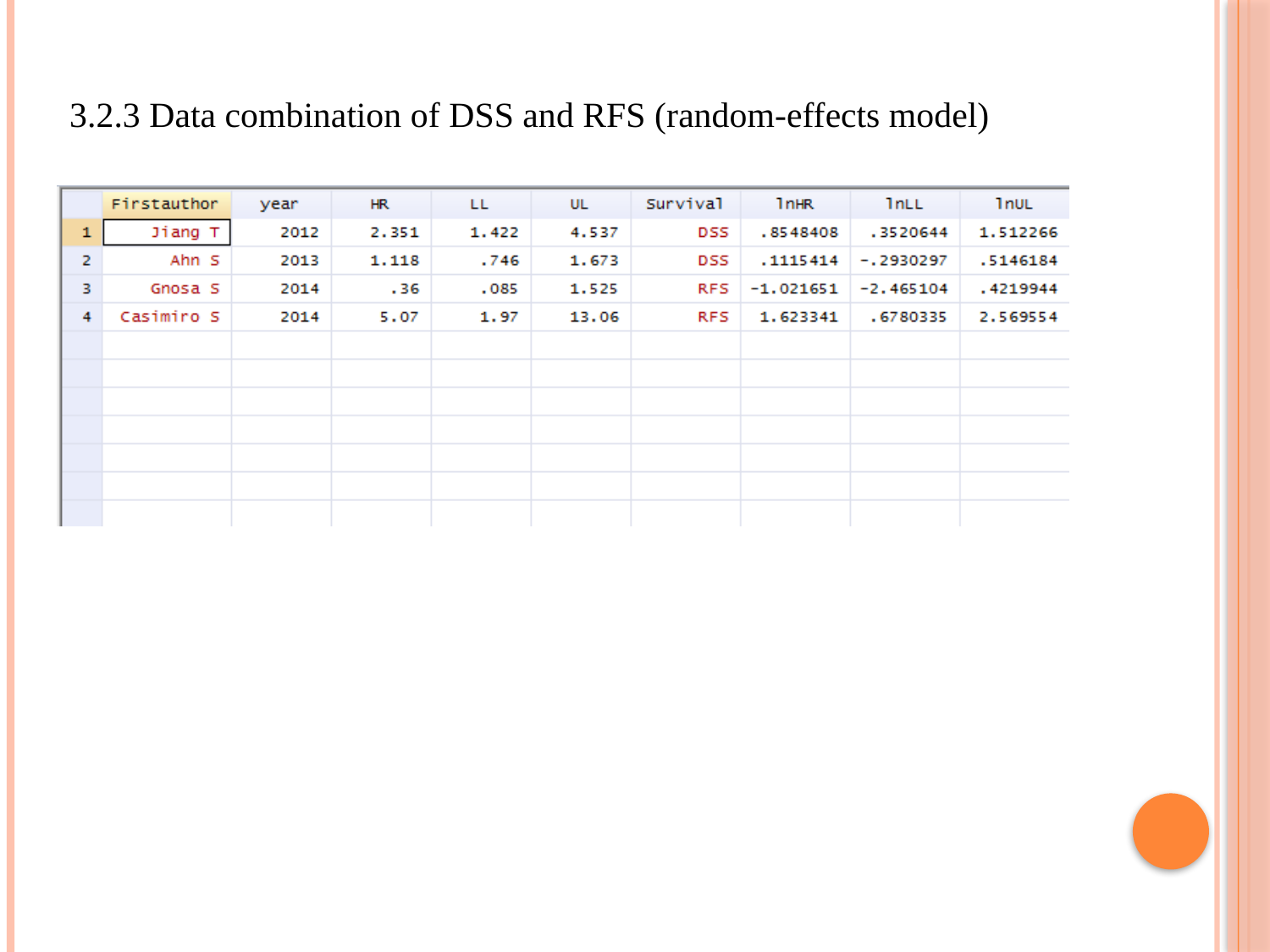

3.2.3 Data combination of DSS and RFS (random-effects model)

## Slide 33
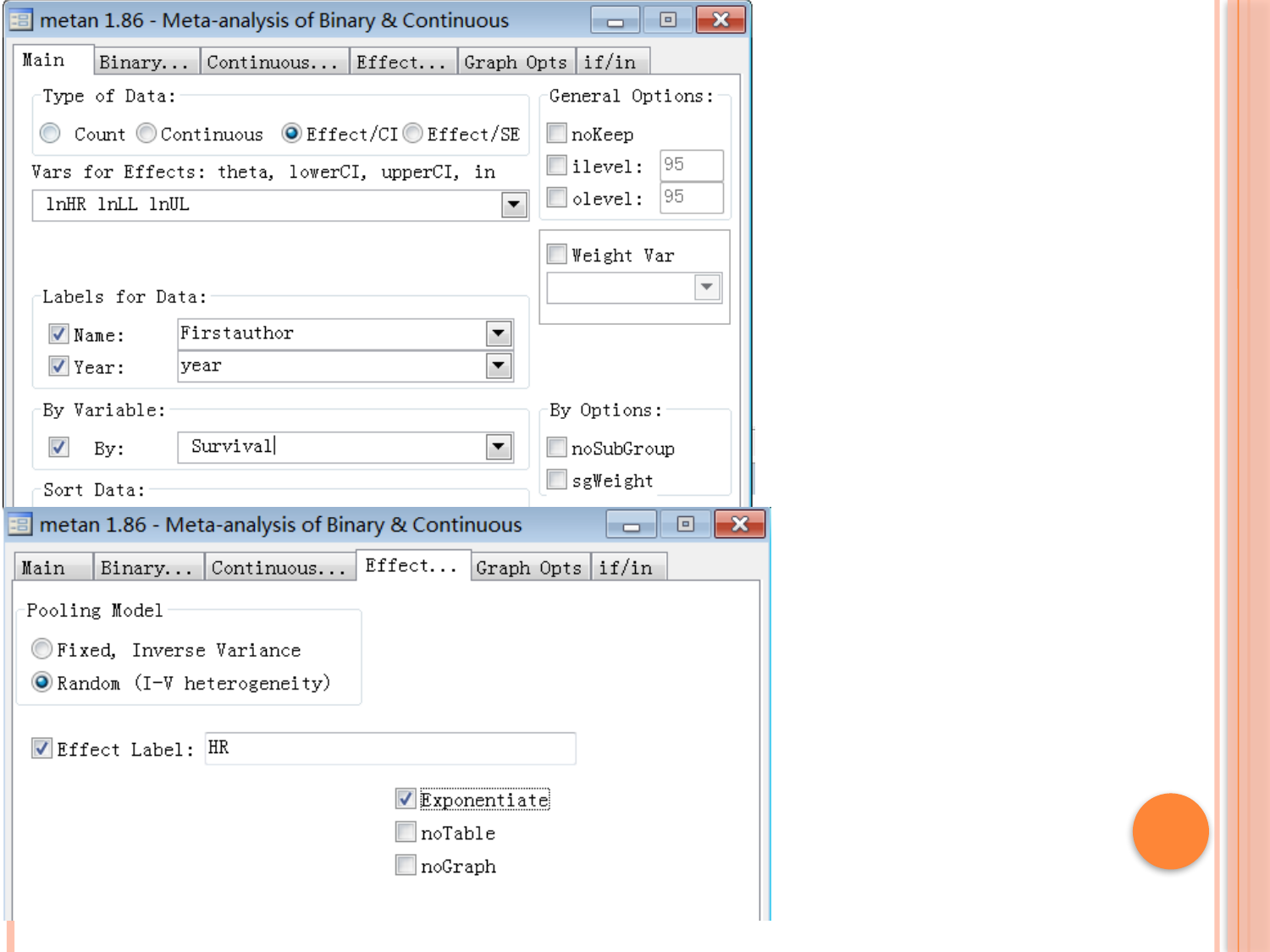

## Slide 34
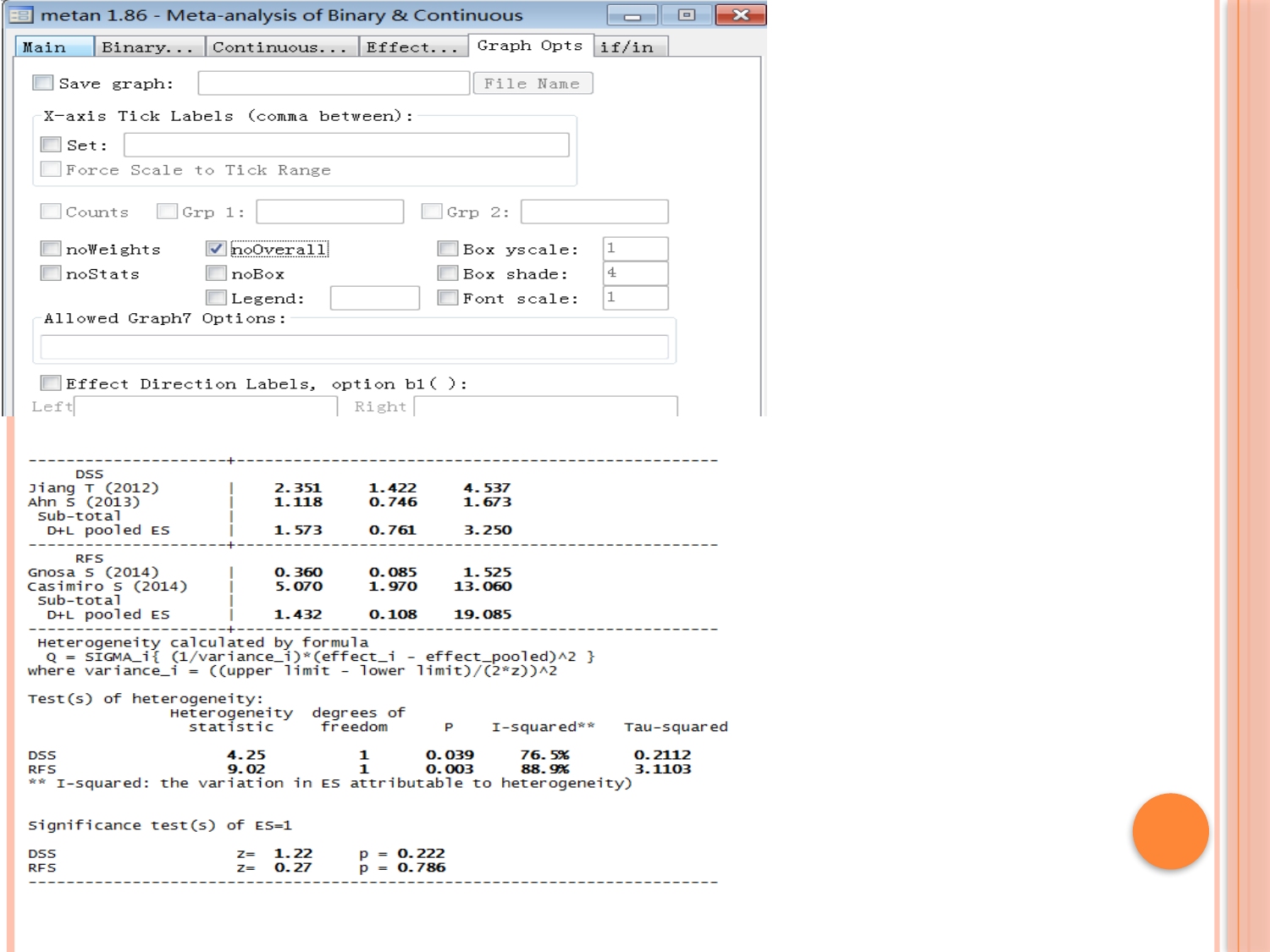

## Slide 35
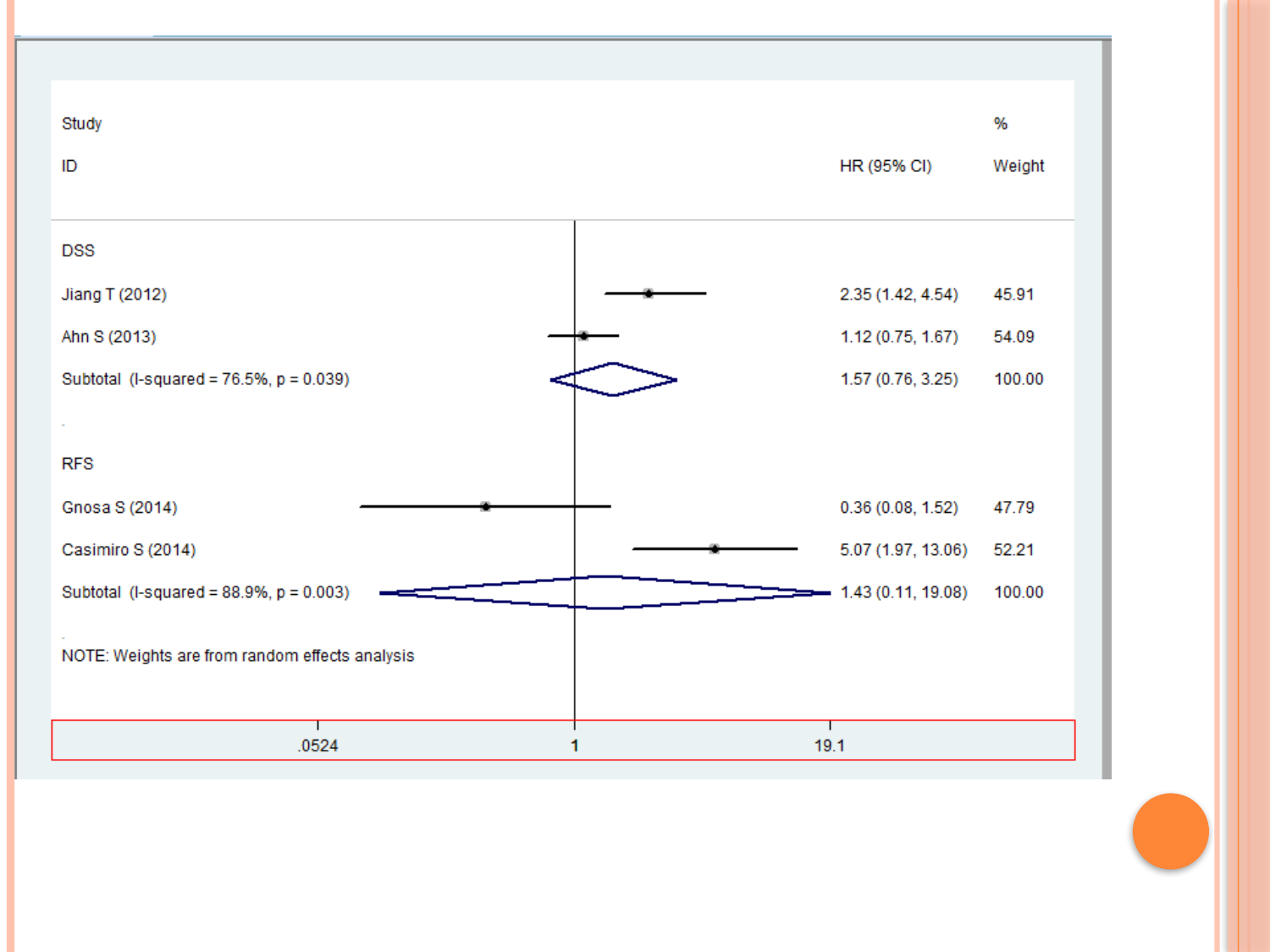

## Slide 36
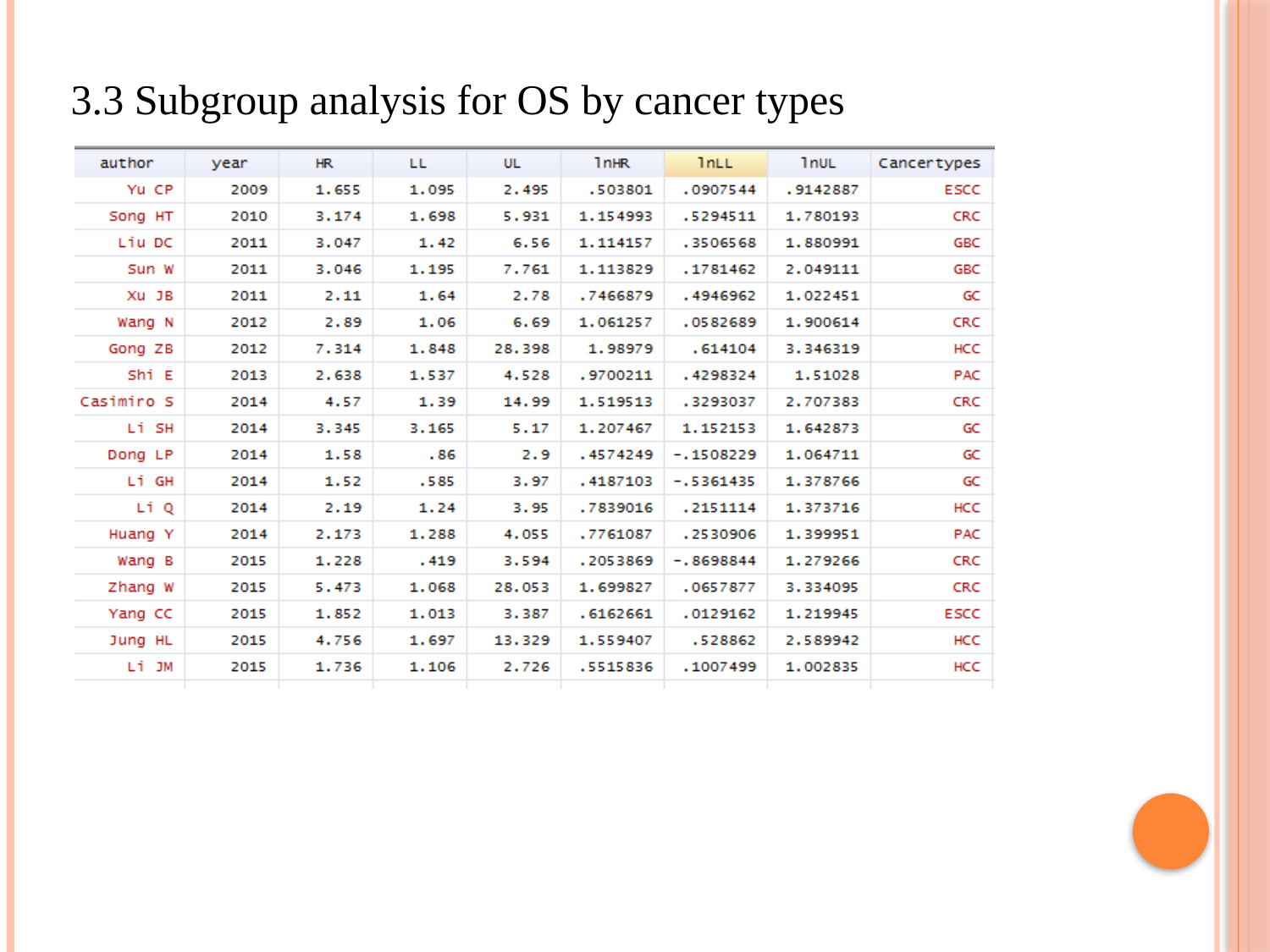

3.3 Subgroup analysis for OS by cancer types

## Slide 37
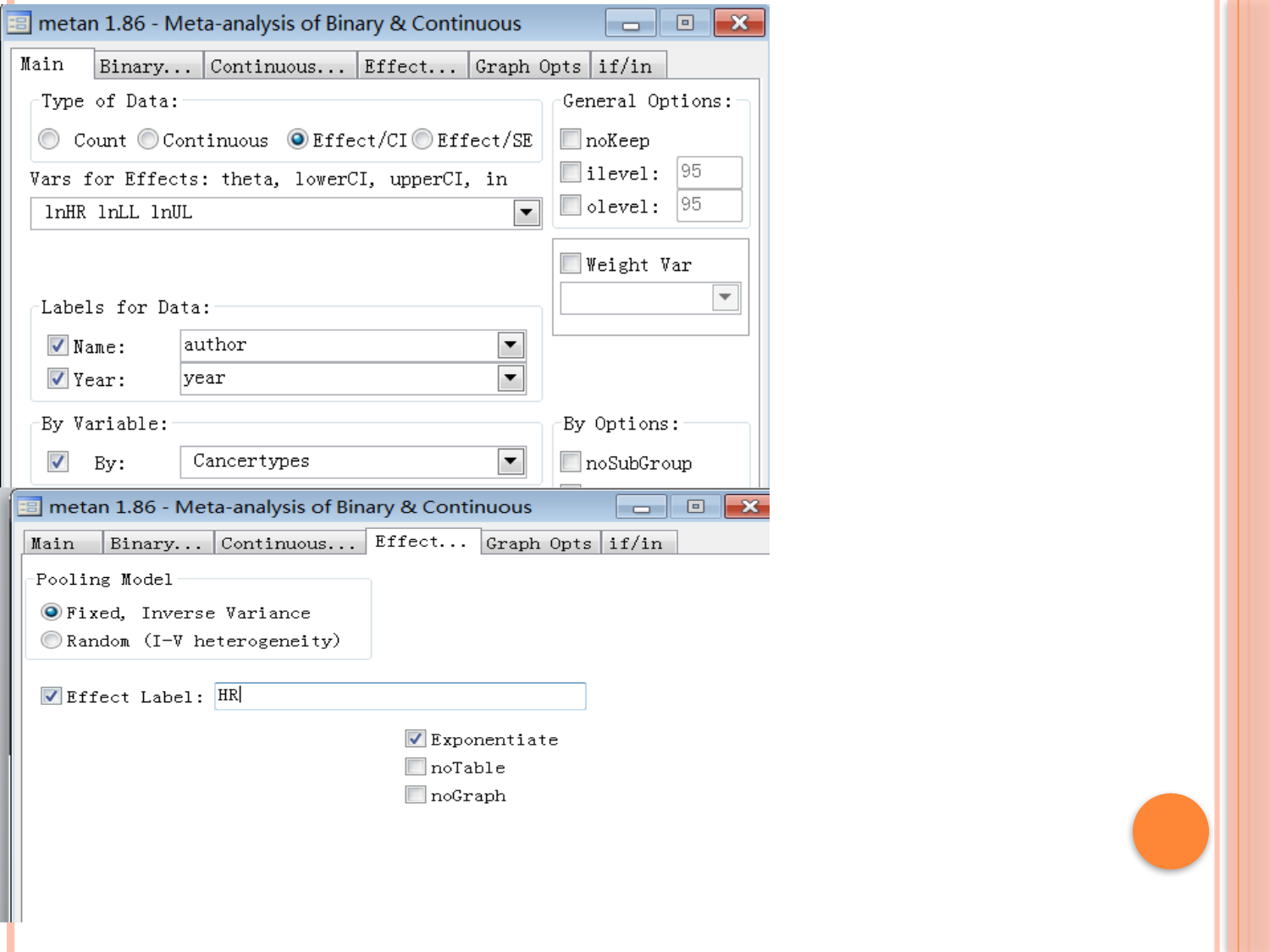

## Slide 38
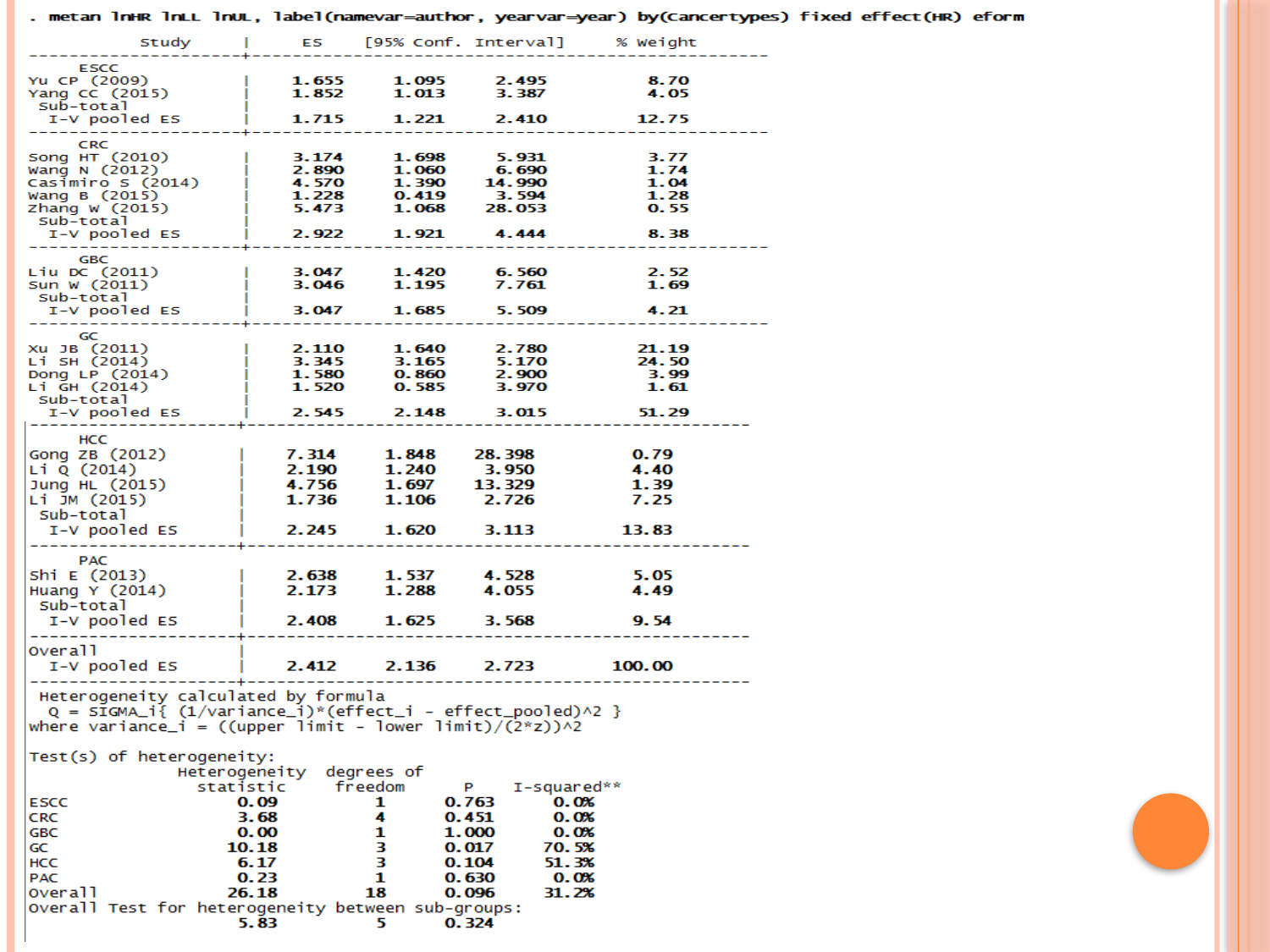

## Slide 39
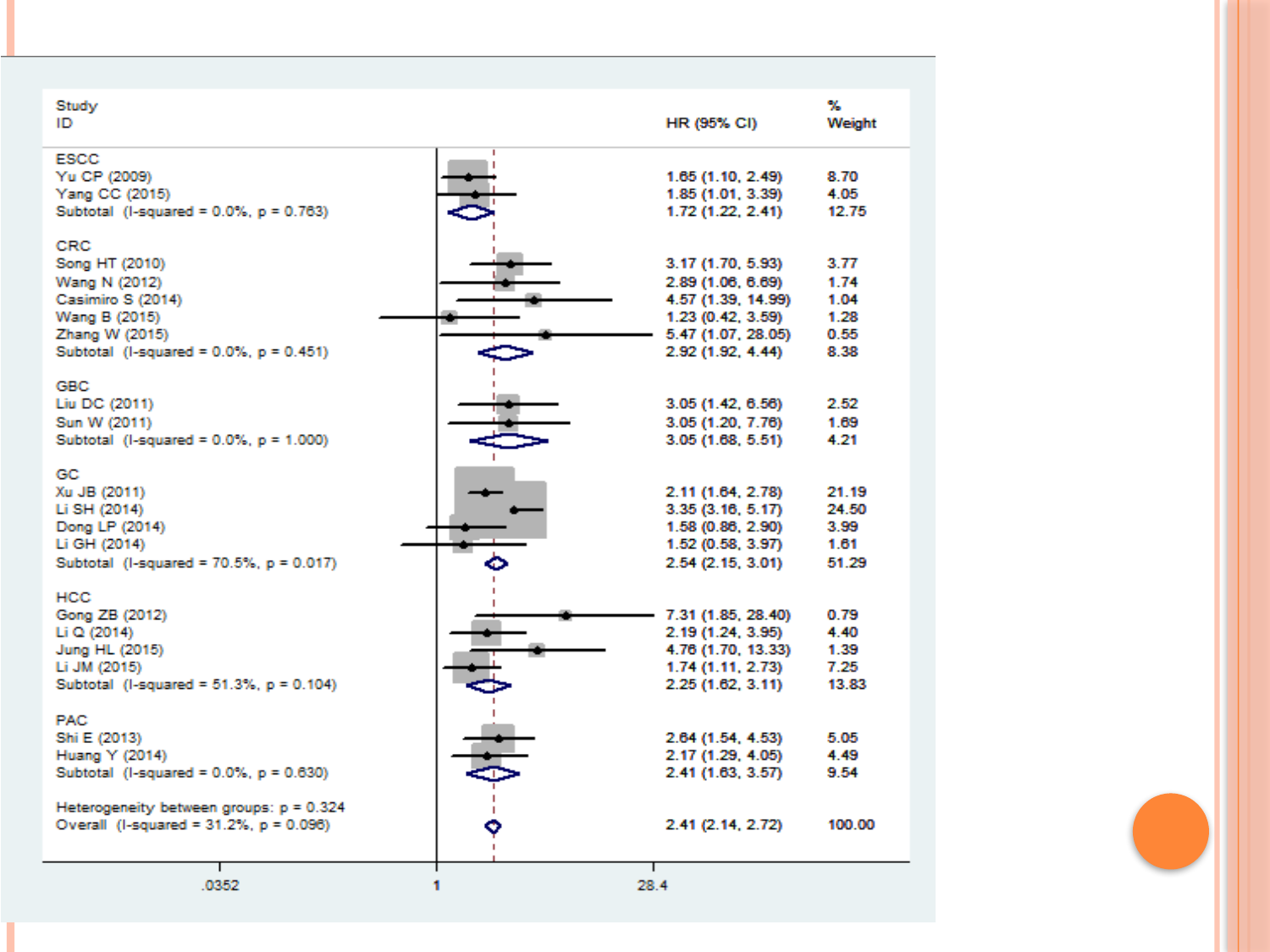

## Slide 40
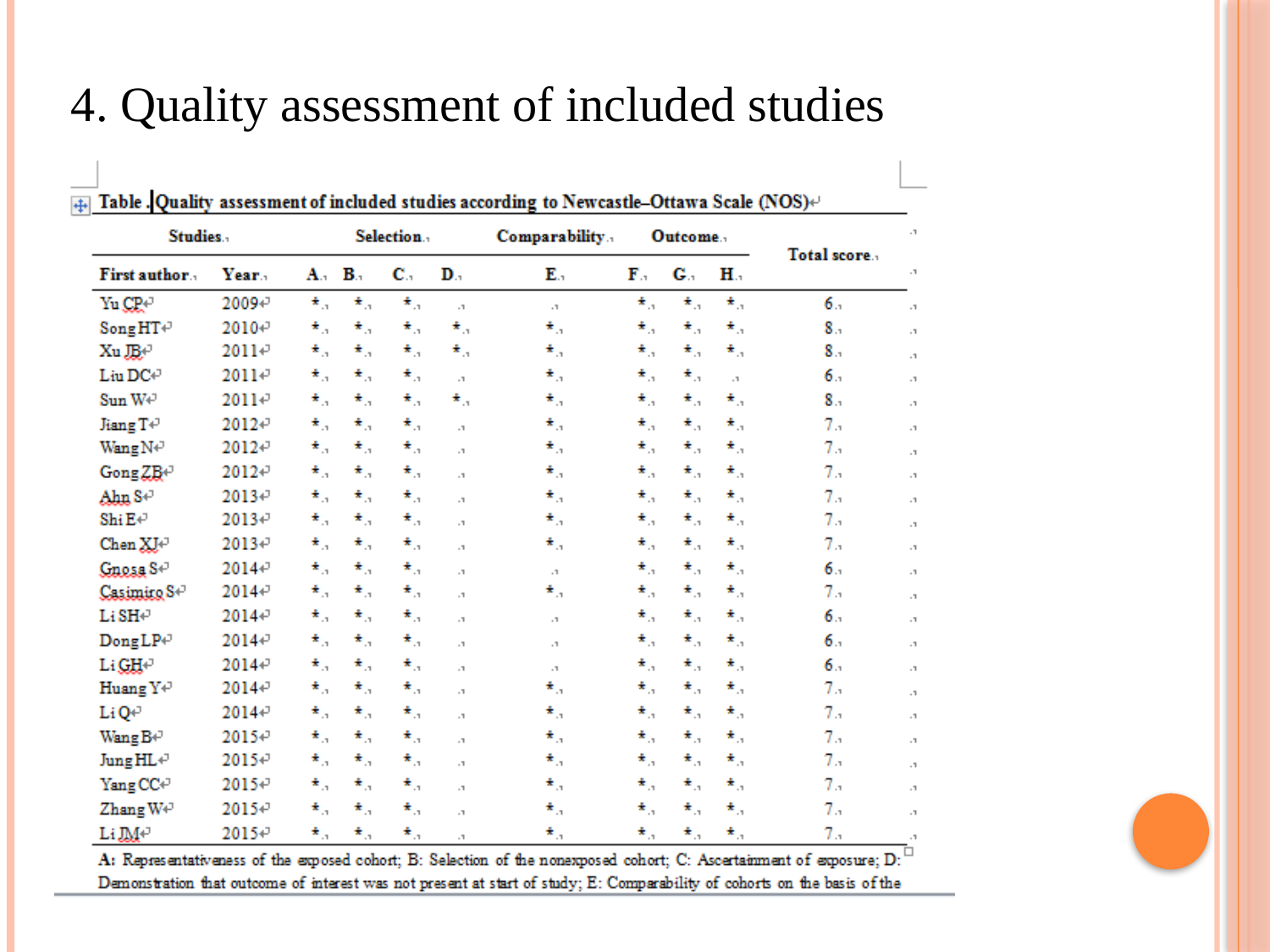

4. Quality assessment of included studies

## Slide 41
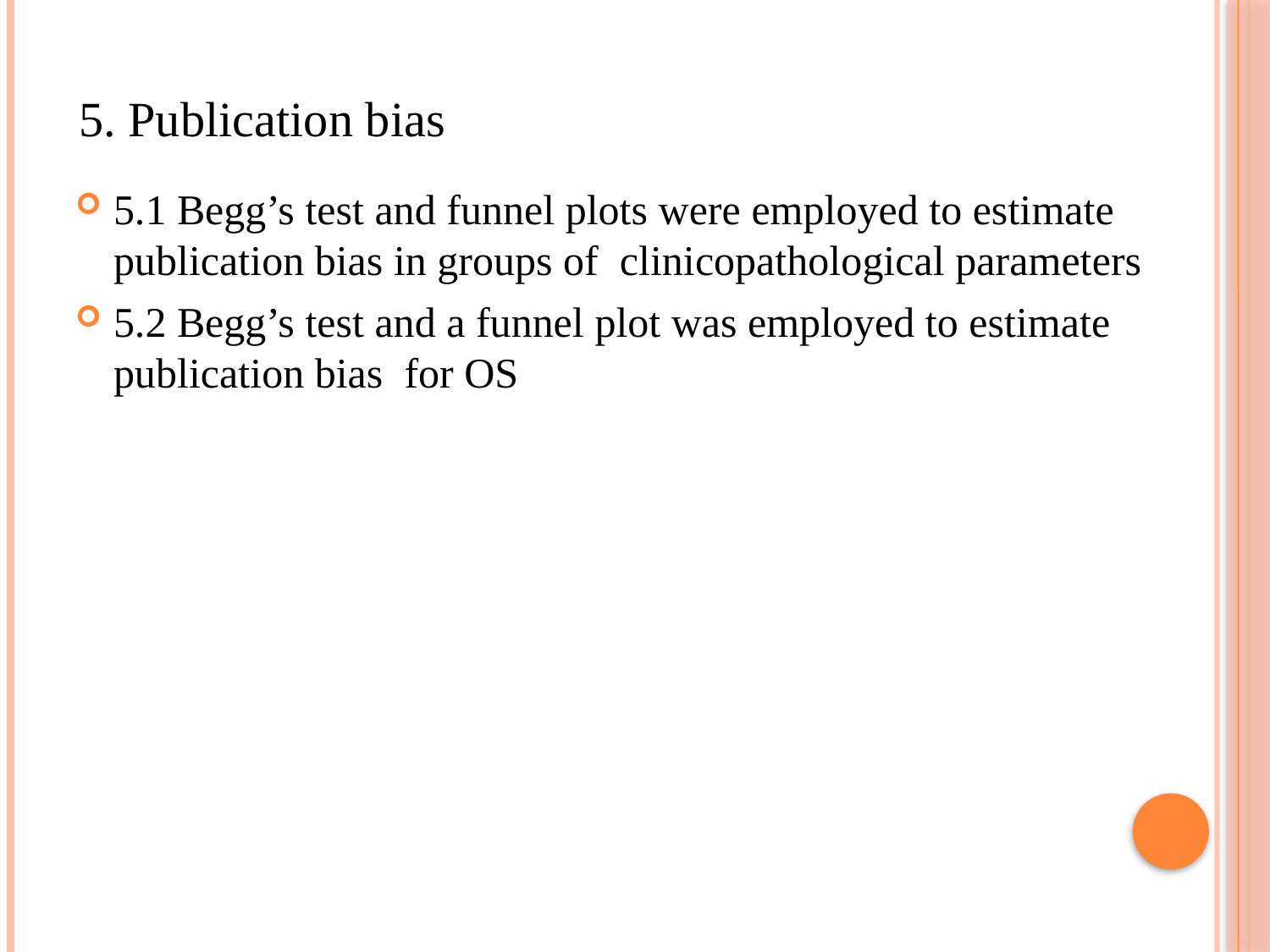

5. Publication bias
5.1 Begg’s test and funnel plots were employed to estimate publication bias in groups of clinicopathological parameters
5.2 Begg’s test and a funnel plot was employed to estimate publication bias for OS

## Slide 42
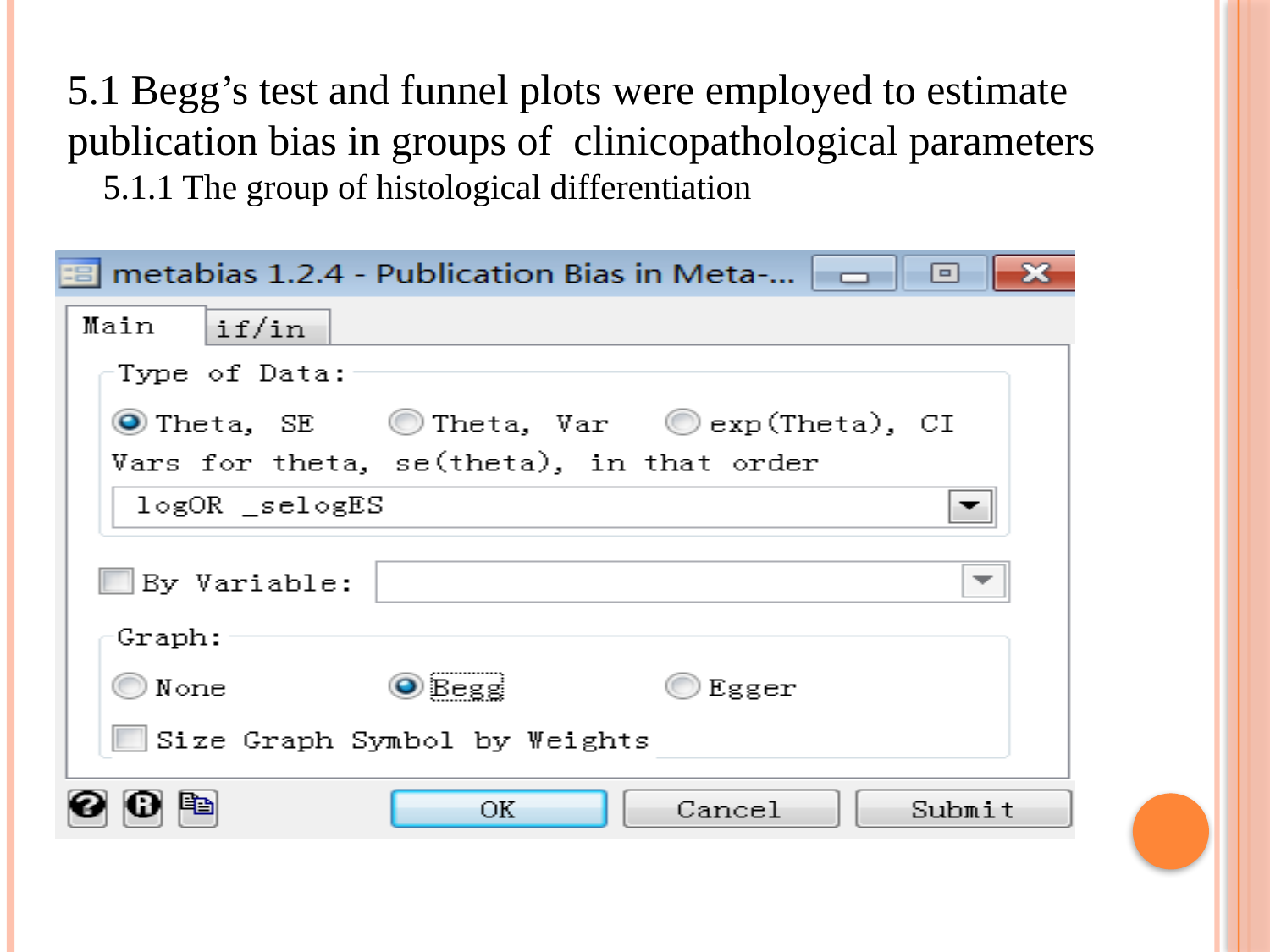

5.1 Begg’s test and funnel plots were employed to estimate publication bias in groups of clinicopathological parameters
 5.1.1 The group of histological differentiation

## Slide 43
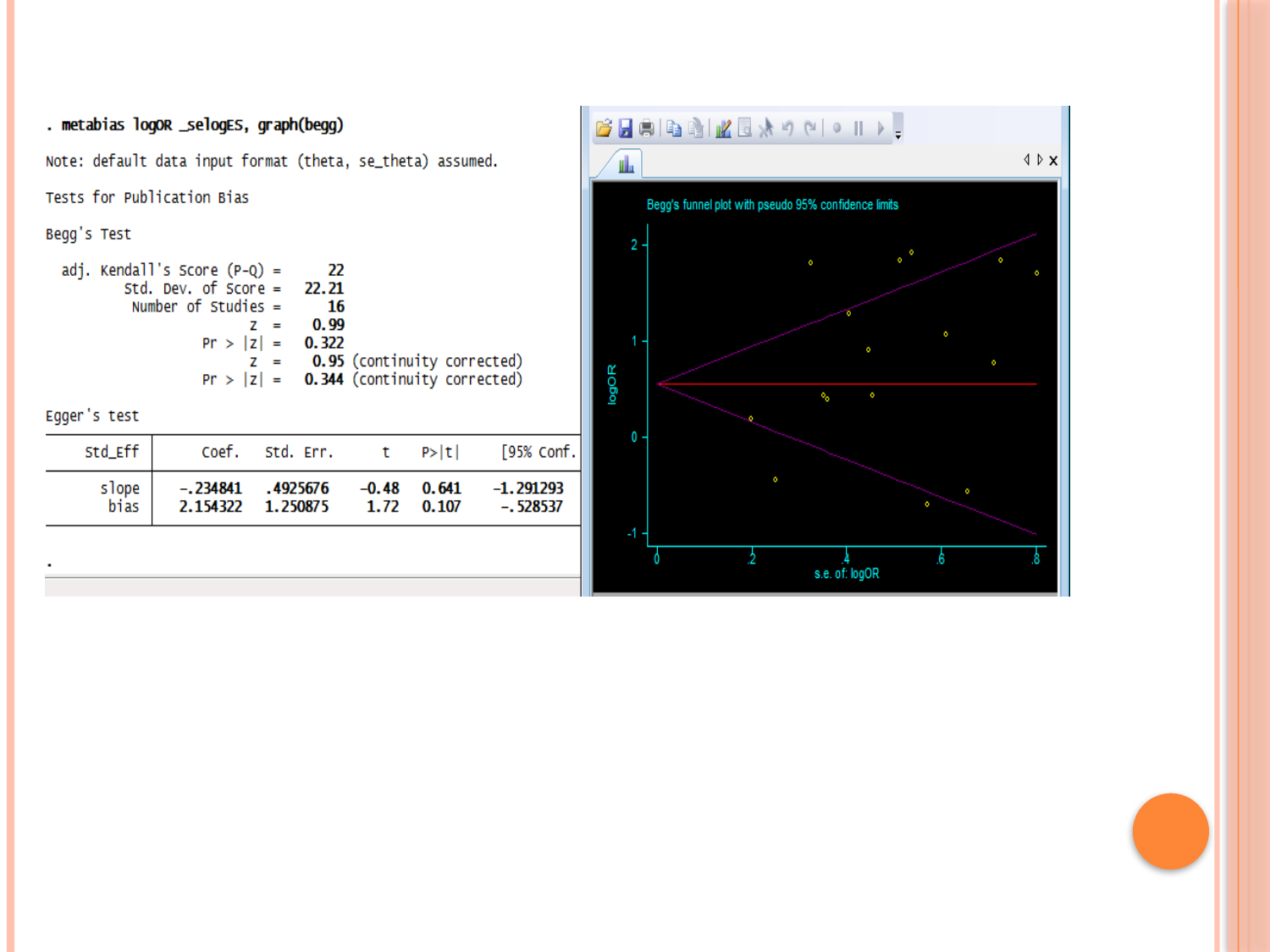

## Slide 44
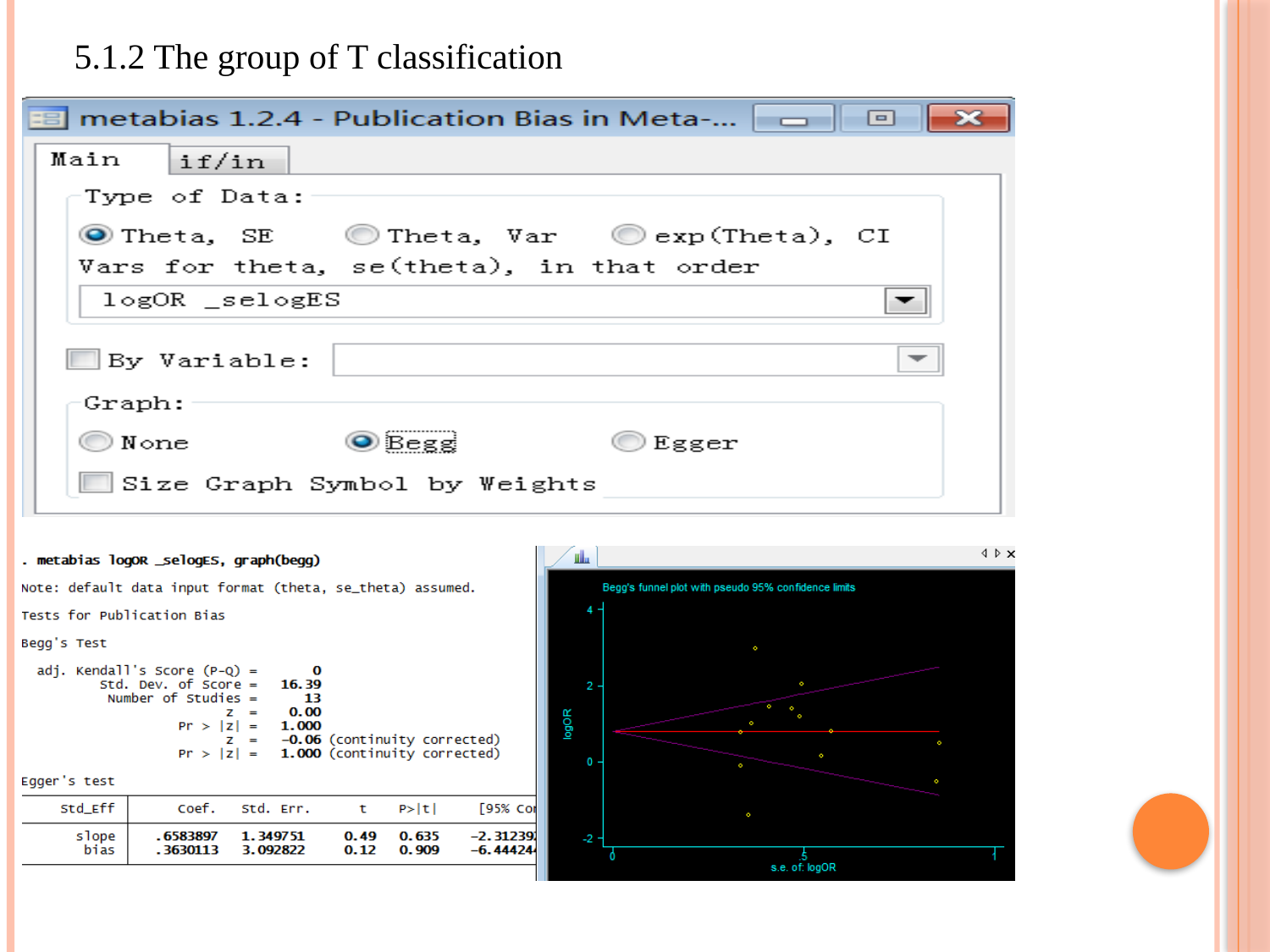

5.1.2 The group of T classification

## Slide 45
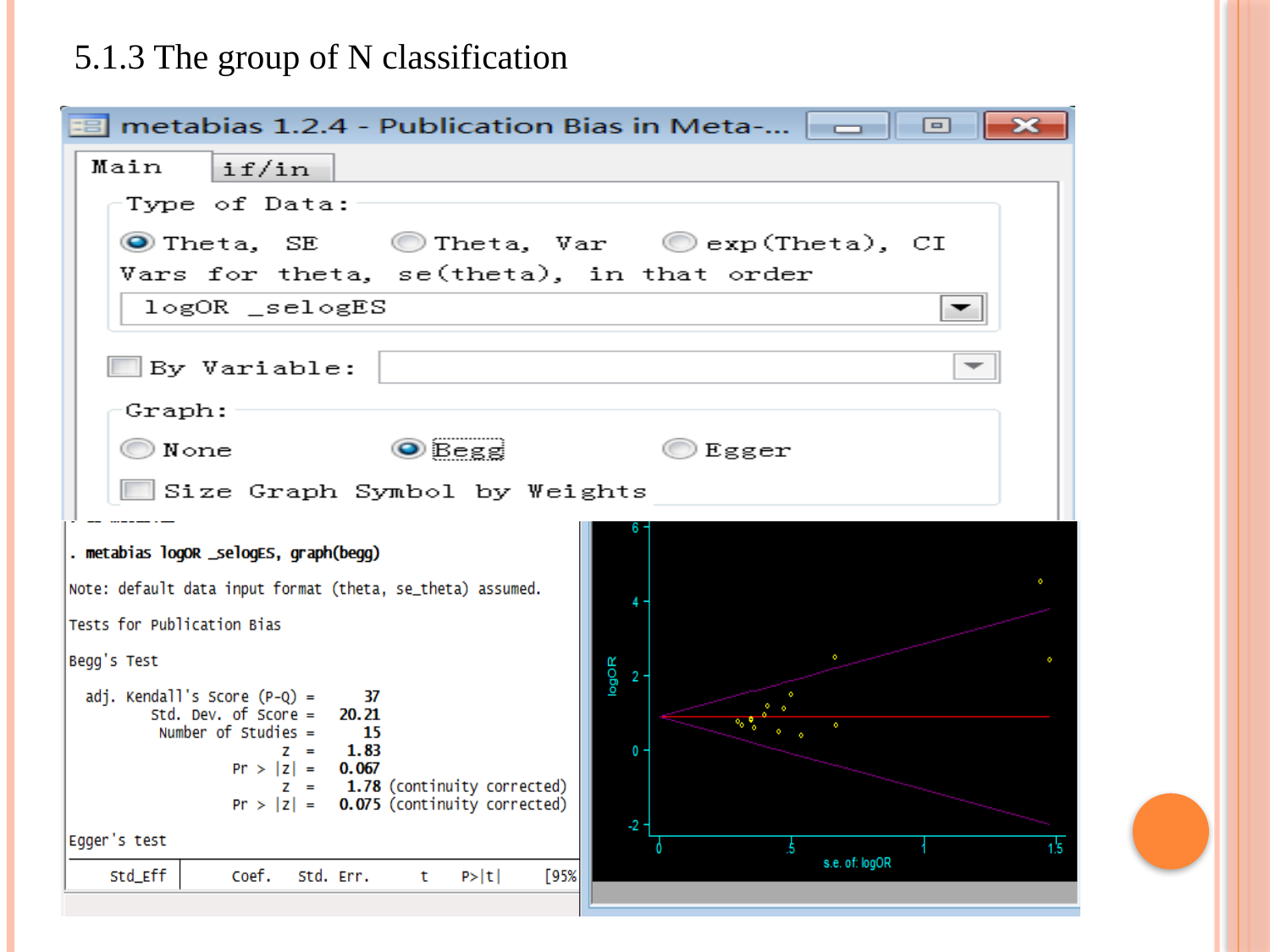

5.1.3 The group of N classification

## Slide 46
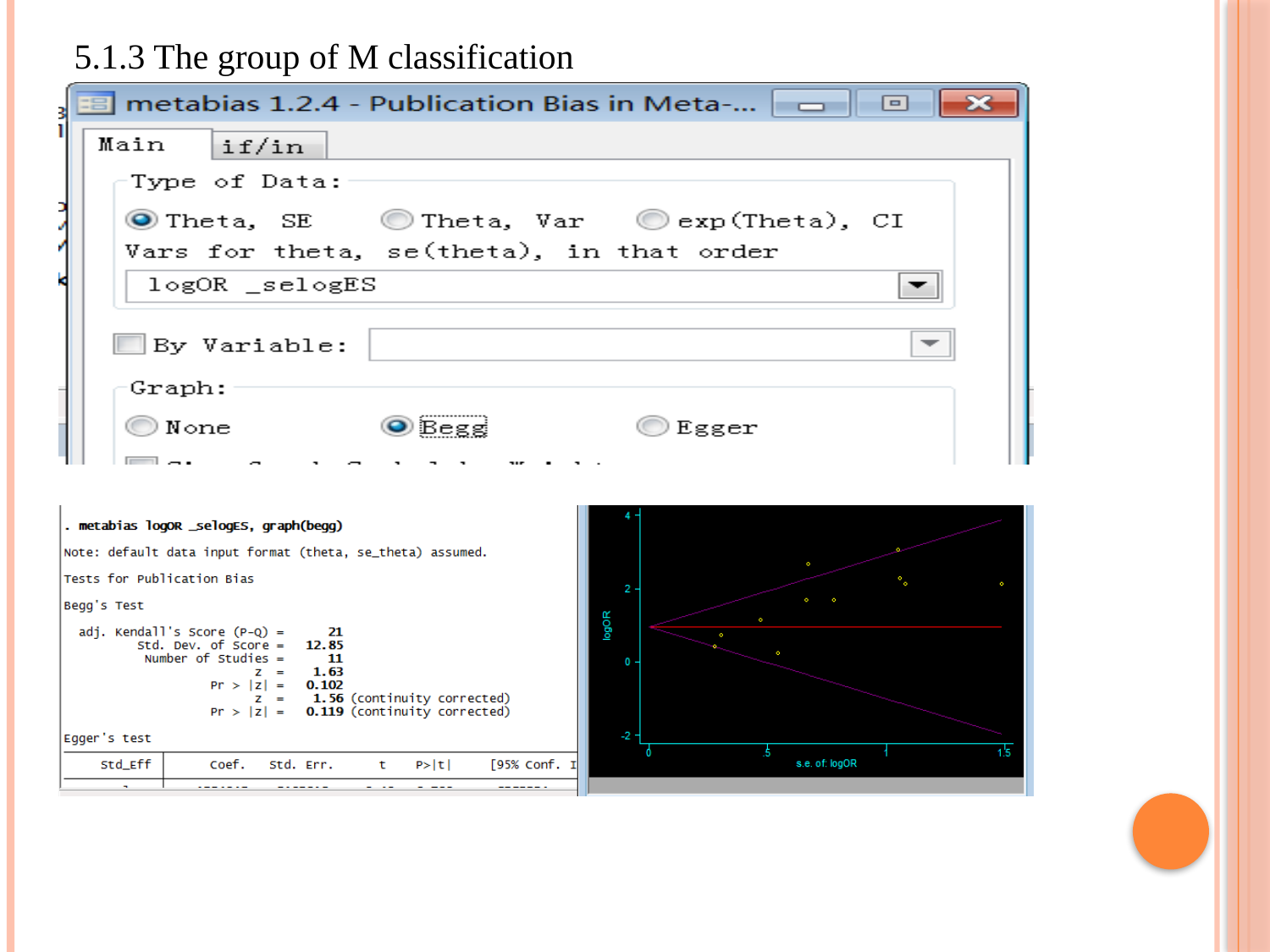

5.1.3 The group of M classification

## Slide 47
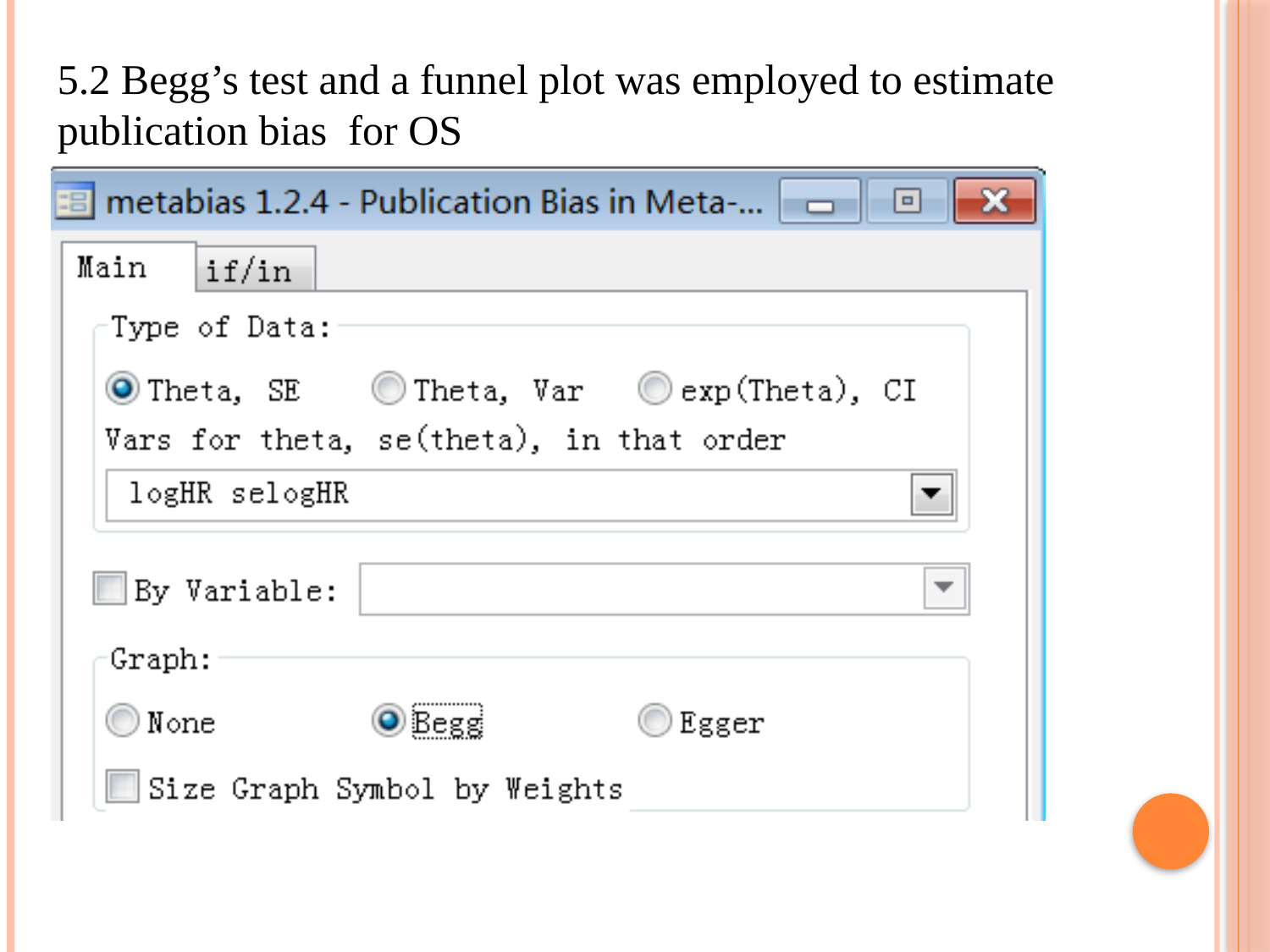

5.2 Begg’s test and a funnel plot was employed to estimate publication bias for OS

## Slide 48
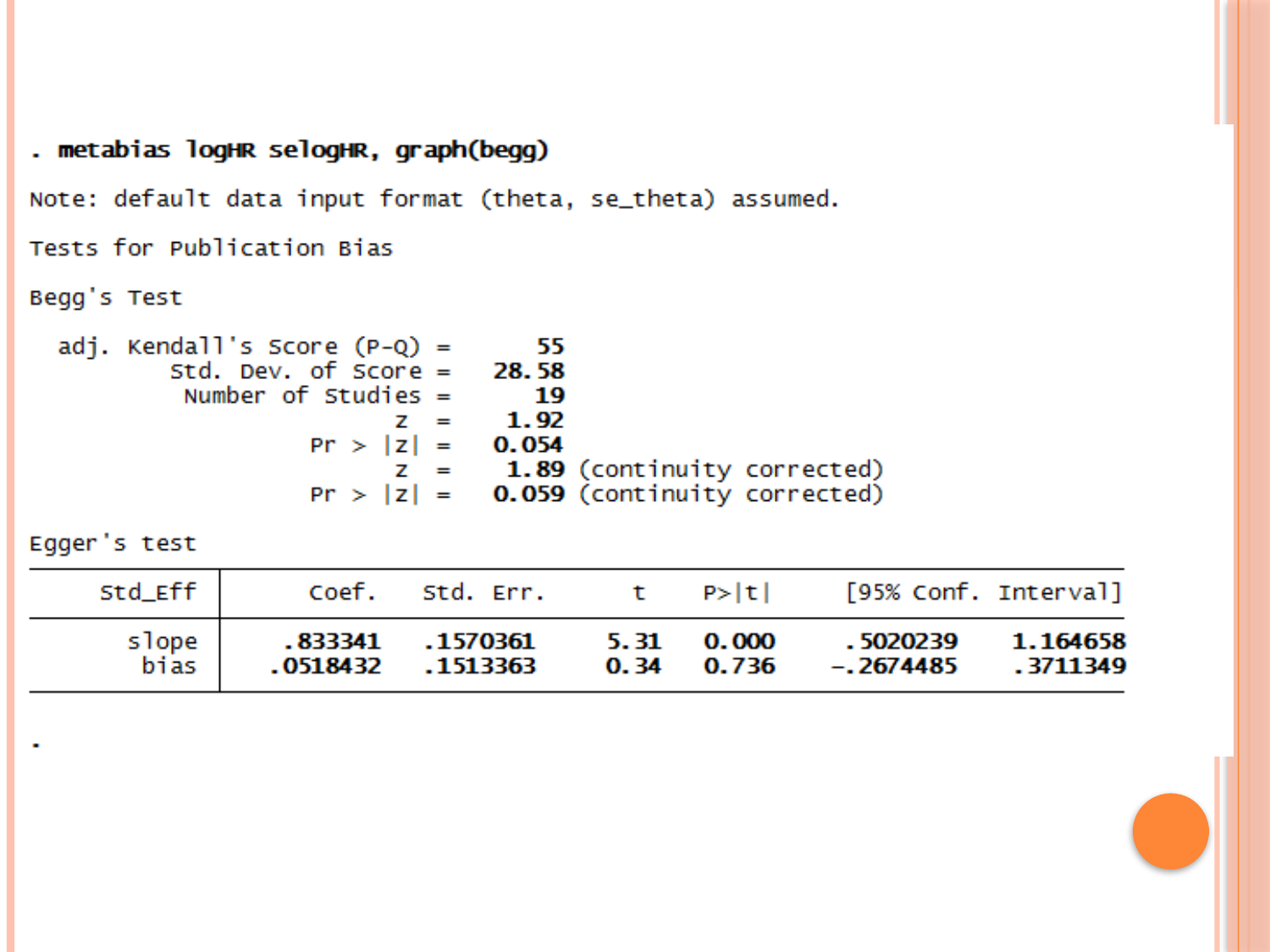

## Slide 49
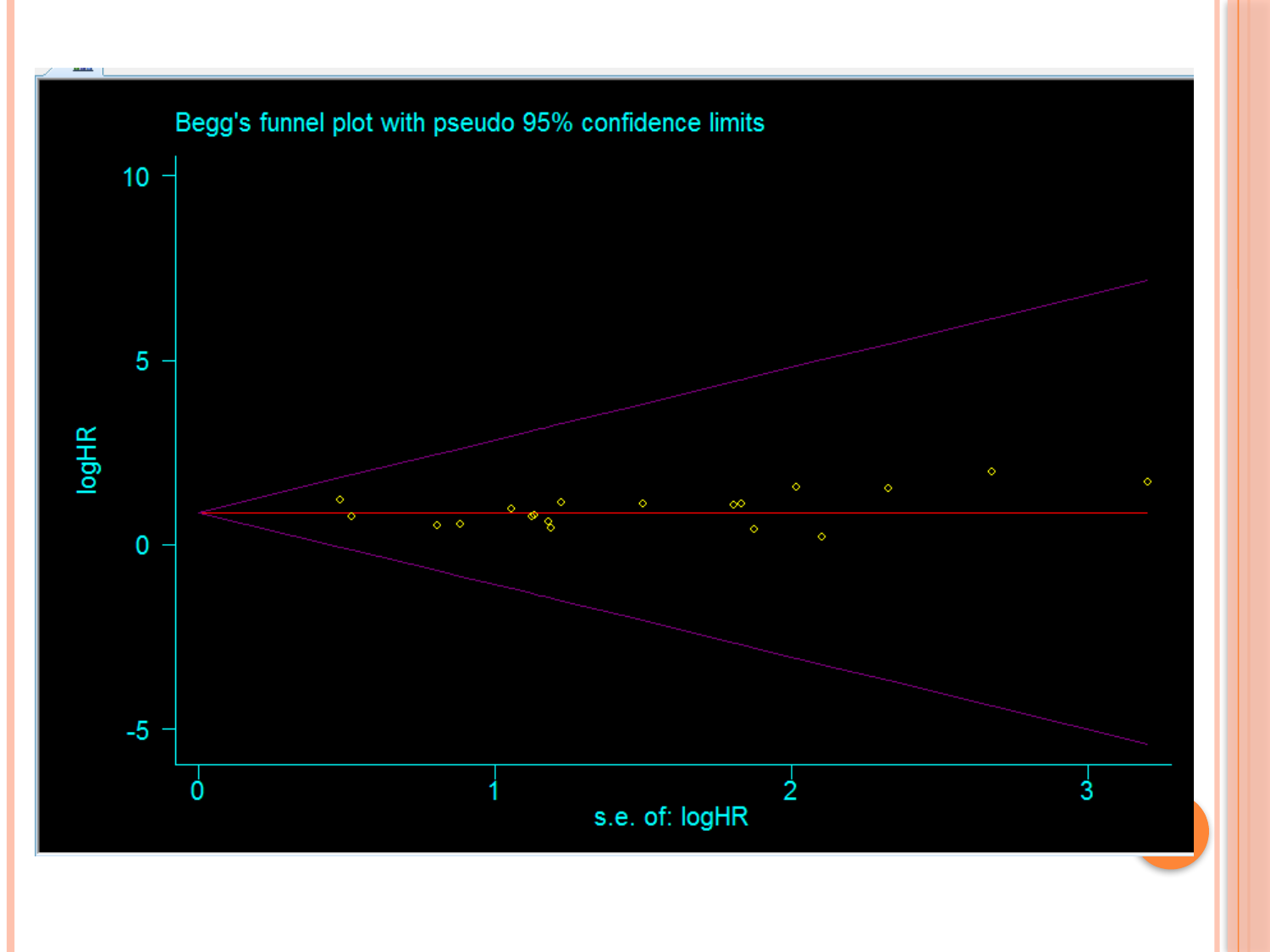

## Slide 50
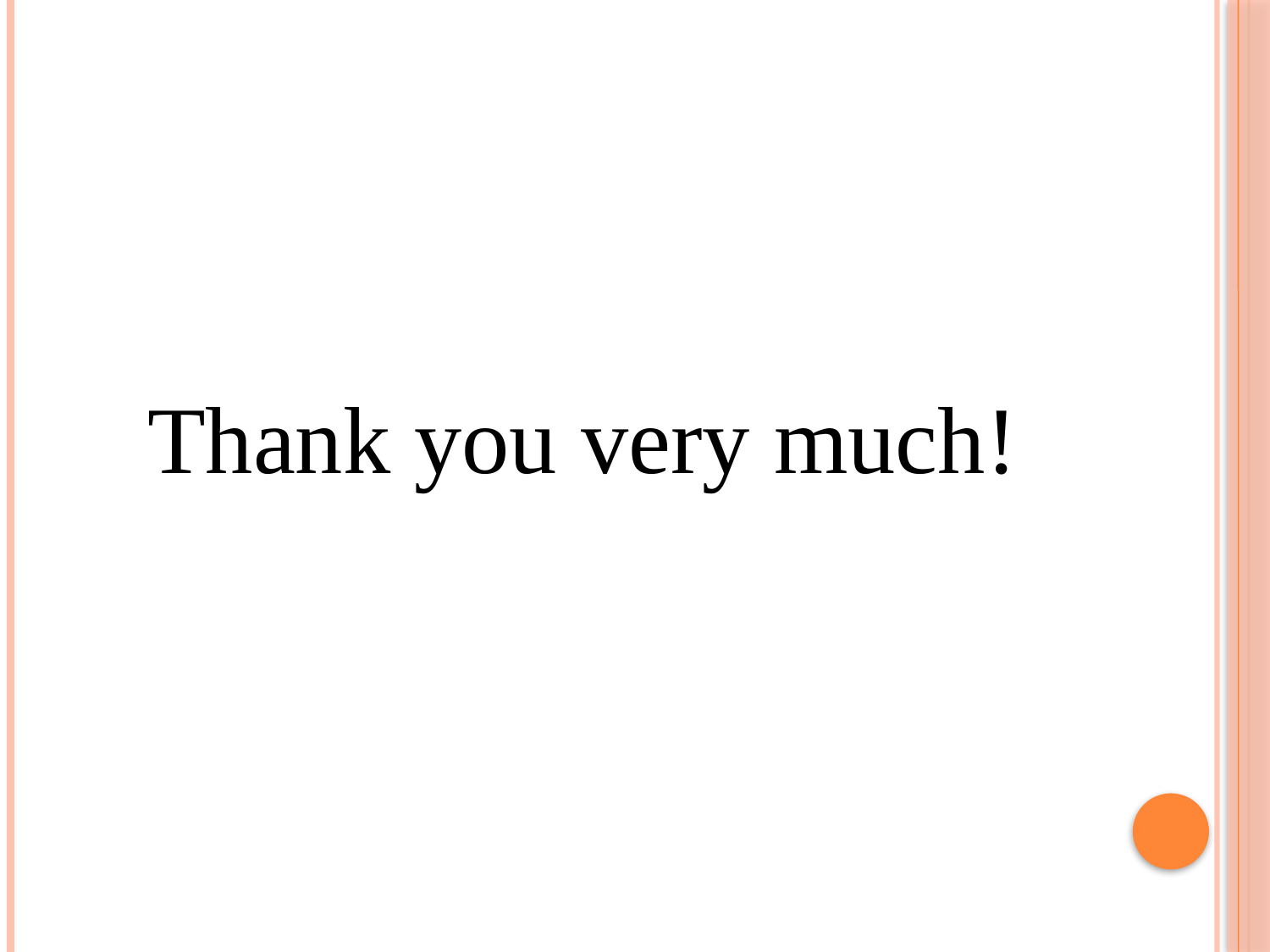

Thank you very much!
