# Supplementary material for: Astrocyte Elevated Gene-1 as a Novel Clinicopathological and Prognostic Biomarker for Gastrointestinal Cancers: A Meta-Analysis with 2999 Patients
Source: PLoS One. 2015 Dec 28;10(12):e0145659. doi: 10.1371/journal.pone.0145659 (PMC4692396; doi:10.1371/journal.pone.0145659)
Supplement: S2 File — (DOCX) [file pone.0145659.s002.docx]

**Studies exclusion**

1. **Studies didn’t provide information for prognosis. [**[**1-29**](#_ENREF_1)**]**
2. **Studies had no effective data to estimated HR and its 95% CI. [**[**30-32**](#_ENREF_30)**]**
3. **Trails using animals and (or) cell lines but not tissues. [**[**33-63**](#_ENREF_33)**]**
4. **Trails detected AEG-1 mRNA expression of AEG-1 by RT-PCR. [**[**64-67**](#_ENREF_64)**]**
5. **Literatures were not published in English or Chinese. [**[**68**](#_ENREF_68)**]**
6. **Reviews. [**[**69-78**](#_ENREF_69)**]**

1. He XX, Chang Y, Meng FY, Wang MY, Xie QH, Tang F, et al. MicroRNA-375 targets AEG-1 in hepatocellular carcinoma and suppresses liver cancer cell growth in vitro and in vivo. Oncogene. 2012;31(28):3357-69. doi: 10.1038/onc.2011.500. PubMed PMID: 22056881.

2. Zhang F, Yang Q, Meng F, Shi H, Li H, Liang Y, et al. Astrocyte elevated gene-1 interacts with beta-catenin and increases migration and invasion of colorectal carcinoma. Molecular carcinogenesis. 2013;52(8):603-10. doi: 10.1002/mc.21894. PubMed PMID: 22431469.

3. Isozaki Y, Hoshino I, Nohata N, Kinoshita T, Akutsu Y, Hanari N, et al. Identification of novel molecular targets regulated by tumor suppressive miR-375 induced by histone acetylation in esophageal squamous cell carcinoma. International journal of oncology. 2012;41(3):985-94. doi: 10.3892/ijo.2012.1537. PubMed PMID: 22752059.

4. Wang CJ, Franbergh-Karlson H, Wang DW, Arbman G, Zhang H, Sun XF. Clinicopathological significance of BTF3 expression in colorectal cancer. Tumour biology : the journal of the International Society for Oncodevelopmental Biology and Medicine. 2013;34(4):2141-6. doi: 10.1007/s13277-013-0745-8. PubMed PMID: 23532689.

5. Huang W, Yang L, Liang S, Liu D, Chen X, Ma Z, et al. AEG-1 is a target of perifosine and is over-expressed in gastric dysplasia and cancers. Digestive diseases and sciences. 2013;58(10):2873-80. doi: 10.1007/s10620-013-2735-5. PubMed PMID: 23912246.

6. Zheng J, Li C, Wu X, Yang Y, Hao M, Sheng S, et al. Astrocyte elevated gene-1 is a novel biomarker of epithelial-mesenchymal transition and progression of hepatocellular carcinoma in two China regions. Tumour biology : the journal of the International Society for Oncodevelopmental Biology and Medicine. 2014;35(3):2265-9. doi: 10.1007/s13277-013-1300-3. PubMed PMID: 24136747.

7. Yang Y, Kang P, Gao J, Xu C, Wang S, Jin H, et al. AU-binding factor 1 expression was correlated with metadherin expression and progression of hepatocellular carcinoma. Tumour biology : the journal of the International Society for Oncodevelopmental Biology and Medicine. 2014;35(3):2747-51. doi: 10.1007/s13277-013-1362-2. PubMed PMID: 24213928.

8. Tang Y, Liu X, Su B, Zhang Z, Zeng X, Lei Y, et al. microRNA-22 acts as a metastasis suppressor by targeting metadherin in gastric cancer. Molecular medicine reports. 2015;11(1):454-60. doi: 10.3892/mmr.2014.2682. PubMed PMID: 25323629.

9. Gan TQ, Tang RX, He RQ, Dang YW, Xie Y, Chen G. Upregulated MiR-1269 in hepatocellular carcinoma and its clinical significance. International journal of clinical and experimental medicine. 2015;8(1):714-21. PubMed PMID: 25785048; PubMed Central PMCID: PMC4358503.

10. Isozaki Y, Hoshino I, Akutsu Y, Hanari N, Mori M, Nishimori T, et al. Usefulness of microRNA375 as a prognostic and therapeutic tool in esophageal squamous cell carcinoma. International journal of oncology. 2015;46(3):1059-66. doi: 10.3892/ijo.2014.2789. PubMed PMID: 25501018.

11. Huang J. Expressive levels of EphA2 and MTDH and its clinicopathological significances in the primary hepatocellular carcinoma and chronic liver diseases. China Journal of Modern Medicine. 2012;(01):49-52+6.

12. Huang Y, Ke R, Xu X, Wang Y, Gan Y, Su Z, et al. Expression of MTDH in Pancreatic Carcinoma and Its Correlations with E -cadherin and Microvascular Density. Journal of Chinese Oncology. 2015;(06):475-81.

13. Luo H, Dong Z, Zou J, Zeng X, Wu D. Expression of AEG-1 gene in colorectal carcinoma and adenoma and its clinical significance. J Trop Med. 2011;(04):411-3+27.

14. Liu K, Wang W, Wei P. The expression of AEG-1, ICAM-1, MMP-2and MMP-9 in gastric cancer and its significance. Journal of Hubei Medical University. 2012;(04):441-3.

15. Zhao X, Jia X, Zhang B. Clinical significance of expression of AEG-1 and MMP-9 in colonic carcinoma patients. China Tropical Medicine. 2014;(03):278-80.

16. Zhao X, Jia X, Zhang B. Expression and clinical significance of AEG-1 and ki-67 in colonic carcinoma. Modern Medical Journal. 2014;(08):892-5.

17. Zhao X, Jia X, Zhang B. Expression and clinical significance of AEG-1 in colonic carcinoma. JOURNAL OF CHENGDE MEDICAL COLLEGE. 2014;(01):19-21.

18. Xie X. Expression and Significance of AEG-1 in Large Intestinal Cancer. J Clin Res. 2014;(7):1343-5,6. doi: 10.3969/j.issn.1671-7171.2014.07.033.

19. Ou Q, Zhao Z, Wang G, Li W. Expression of Metaherin and Its Clinical Significance for Hepatitis B Related Hepatocellular Carcinoma. Chin J Bases Clin General Surg. 2015;(03):301-6.

20. Liu X, Wu C, Xu H, Wang L. The expression of AEG-1 and MVD correlated with tumor metastasis and prognosis in ESCC. JOURNAL OF FUJIAN MEDICAL COLLEGE. 2012;(01):24-7.

21. Yang F, Deng Y, Liu G, Chang J, Li G, Hu S, et al. The expression and significance of MTDH and DCD4 in gastric cancer. Acta Universitatis Medicinalis Anhui 2014;(07):954-7.

22. Liu K, Li Y, Wang W. Relationship between the expression of AEG-1 and clinicopathologicai characteristics in gastric carcinoma. HuBei Medical Journal. 2012;(05):653-5.

23. Deng W, Huang W, Yang C, Huang J, Dai J, Wang J. Expression of AEG-1 and CyclinD1 in gastric adenocarcinoma and their clinicopathologic significance. J Clin Exp Pathol. 2014;(10):1104-6+12.

24. Yin H, Zhao H, Huang X, Jin G. The relationship between the expression of MTDH in gastric adenocacinoma tissues and clinicopathological characteristics. JOURNAL OF QINGHAI MEDICAL COLLEGE. 2014;(04):238-42.

25. Zhao X, Jia X, Zhang B, Geng S. The expression and its significance of MTDH and p53 in colon cancer. J Clin Intern Med. 2013;30(10):703-4. doi: 10.3969/j.issn.1001-9057.2013.10.020.

26. Yu Y, Li C, Zhang J, Sun Y, Sun S. Expression and clinical significance of astrocyte elevated gene-1 and vascular endothelial growth factor in cholangiocarcinoma. Chin J Exp Surg. 2013;30(3):624-6. doi: 10.3760/cma.j.issn.1001-9030.2013.03.072.

27. Li C, Zhang W, Liu X, Liu B, Zhang H, Yang G, et al. Tissue microarray detection for astrocyte elevated gene-1 and its correlation with pathological factors in primary hepatocellular carcinoma. Chinese Journal of Tissue Engineering Research. 2015;(20):3147-51.

28. Jiang L, Yu H, Xiao F. The expression and its significance of MTDH in gastric carcinoma. J Diag Pathol. 2015;(05):304-6.

29. Ma A, Li F, Jiang H, Qin J. The expression and significance of MTDH in gastric carcinoma. Ningxia Med J. 2014;(12).

30. Gnosa S, Shen YM, Wang CJ, Zhang H, Stratmann J, Arbman G, et al. Expression of AEG-1 mRNA and protein in colorectal cancer patients and colon cancer cell lines. Journal of translational medicine. 2012;10:109. Epub 2012/05/31. doi: 10.1186/1479-5876-10-109. PubMed PMID: 22643064; PubMed Central PMCID: PMCPmc3464714.

31. Zhang N, Li X, Li G, Wang G. The expression and significance of AEG-1 and LEF-1 in colon carcinoma tissues. The Journal of Practical Medicine. 2015;(07):1160-2.

32. Chen L. Significance of Expression And SNPs of MTDH in Gastric cancer [Master]: FuJian Medical University; 2011.

33. Yoo BK, Emdad L, Su Z, Villanueva A, Chiang DY, Mukhopadhyay ND, et al. Astrocyte elevated gene-1 regulates hepatocellular carcinoma development and progression. Journal of Clinical Investigation. 2009;119(3):465-77. doi: 10.1172/jci36460. PubMed PMID: WOS:000263941000010.

34. Li J, Chen Y, Zhao J, Kong F, Zhang Y. miR-203 reverses chemoresistance in p53-mutated colon cancer cells through downregulation of Akt2 expression. Cancer letters. 2011;304(1):52-9. Epub 2011/03/01. doi: 10.1016/j.canlet.2011.02.003. PubMed PMID: 21354697.

35. Yoo BK, Santhekadur PK, Gredler R, Chen D, Emdad L, Bhutia S, et al. Increased RNA-induced silencing complex (RISC) activity contributes to hepatocellular carcinoma. Hepatology (Baltimore, Md). 2011;53(5):1538-48. Epub 2011/04/27. doi: 10.1002/hep.24216. PubMed PMID: 21520169; PubMed Central PMCID: PMCPmc3081619.

36. Li J, Zhang Y, Zhao J, Kong F, Chen Y. Overexpression of miR-22 reverses paclitaxel-induced chemoresistance through activation of PTEN signaling in p53-mutated colon cancer cells. Molecular and cellular biochemistry. 2011;357(1-2):31-8. Epub 2011/05/20. doi: 10.1007/s11010-011-0872-8. PubMed PMID: 21594648.

37. Chen D, Yoo BK, Santhekadur PK, Gredler R, Bhutia SK, Das SK, et al. Insulin-like growth factor-binding protein-7 functions as a potential tumor suppressor in hepatocellular carcinoma. Clinical cancer research : an official journal of the American Association for Cancer Research. 2011;17(21):6693-701. Epub 2011/09/13. doi: 10.1158/1078-0432.ccr-10-2774. PubMed PMID: 21908579; PubMed Central PMCID: PMCPmc3207018.

38. Srivastava J, Siddiq A, Emdad L, Santhekadur PK, Chen D, Gredler R, et al. Astrocyte elevated gene-1 promotes hepatocarcinogenesis: novel insights from a mouse model. Hepatology (Baltimore, Md). 2012;56(5):1782-91. Epub 2012/06/13. doi: 10.1002/hep.25868. PubMed PMID: 22689379; PubMed Central PMCID: PMCPmc3449036.

39. Wang K, Lim HY, Shi S, Lee J, Deng S, Xie T, et al. Genomic landscape of copy number aberrations enables the identification of oncogenic drivers in hepatocellular carcinoma. Hepatology (Baltimore, Md). 2013;58(2):706-17. Epub 2013/03/19. doi: 10.1002/hep.26402. PubMed PMID: 23505090.

40. Zhang CF, Xia YH, Zheng QF, Li ZJ, Guo XH, Zhou HC, et al. [Effect of silencing AEG-1 with small interfering RNA on the proliferation and cell cycle of gastric carcinoma SGC-7901 cells]. Zhonghua zhong liu za zhi [Chinese journal of oncology]. 2013;35(1):22-7. Epub 2013/05/08. doi: 10.3760/cma.j.issn.0253-3766.2013.01.005. PubMed PMID: 23648295.

41. Ma J, Xie SL, Geng YJ, Jin S, Wang GY, Lv GY. In vitro regulation of hepatocellular carcinoma cell viability, apoptosis, invasion, and AEG-1 expression by LY294002. Clinics and research in hepatology and gastroenterology. 2014;38(1):73-80. Epub 2013/08/06. doi: 10.1016/j.clinre.2013.06.012. PubMed PMID: 23910058.

42. Zheng J, Li C, Wu X, Liu M, Sun X, Yang Y, et al. Huaier polysaccharides suppresses hepatocarcinoma MHCC97-H cell metastasis via inactivation of EMT and AEG-1 pathway. International journal of biological macromolecules. 2014;64:106-10. Epub 2013/12/11. doi: 10.1016/j.ijbiomac.2013.11.034. PubMed PMID: 24321491.

43. Deng H, Zhou ZZ, Tu W, Xia YJ, Huang HJ, Tian D. Knockdown of astrocyte elevated gene-1 inhibits growth through suppression of IL-6 secretion in HepG2 human hepatoma cells. Oncology Letters. 2014;7(1):101-6. PubMed PMID: WOS:000330783400019.

44. Zou YM, Xiong H, Xiong HH, Lu T, Zhu F, Luo ZY, et al. A polysaccharide from mushroom Huaier retards human hepatocellular carcinoma growth, angiogenesis, and metastasis in nude mice. Tumor Biology. 2015;36(4):2929-36. doi: 10.1007/s13277-014-2923-8. PubMed PMID: WOS:000352885900082.

45. Shen X, Si Y, Yang Z, Wang Q, Yuan J, Zhang X. MicroRNA-542-3p suppresses cell growth of gastric cancer cells via targeting oncogene astrocyte-elevated gene-1. Medical oncology (Northwood, London, England). 2015;32(1):361. Epub 2014/11/30. doi: 10.1007/s12032-014-0361-5. PubMed PMID: 25432696.

46. Zhao J, Wang W, Huang Y, Wu J, Chen M, Cui P, et al. HBx elevates oncoprotein AEG-1 expression to promote cell migration by downregulating miR-375 and miR-136 in malignant hepatocytes. DNA and cell biology. 2014;33(10):715-22. Epub 2014/07/23. doi: 10.1089/dna.2014.2376. PubMed PMID: 25050974.

47. Robertson CL, Srivastava J, Siddiq A, Gredler R, Emdad L, Rajasekaran D, et al. Genetic deletion of AEG-1 prevents hepatocarcinogenesis. Cancer research. 2014;74(21):6184-93. Epub 2014/09/07. doi: 10.1158/0008-5472.can-14-1357. PubMed PMID: 25193383; PubMed Central PMCID: PMCPmc4216744.

48. Srivastava J, Siddiq A, Gredler R, Shen XN, Rajasekaran D, Robertson CL, et al. Astrocyte elevated gene-1 and c-Myc cooperate to promote hepatocarcinogenesis in mice. Hepatology (Baltimore, Md). 2015;61(3):915-29. Epub 2014/07/30. doi: 10.1002/hep.27339. PubMed PMID: 25065684; PubMed Central PMCID: PMCPmc4309751.

49. Zhu K, Pan Q, Jia LQ, Dai Z, Ke AW, Zeng HY, et al. MiR-302c inhibits tumor growth of hepatocellular carcinoma by suppressing the endothelial-mesenchymal transition of endothelial cells. Scientific reports. 2014;4:5524. Epub 2014/07/17. doi: 10.1038/srep05524. PubMed PMID: 25027009; PubMed Central PMCID: PMCPmc4100019.

50. Zhou ZZ, Deng H, Yan W, Luo M, Tu W, Xia YJ, et al. AEG-1 Promotes Anoikis Resistance and Orientation Chemotaxis in Hepatocellular Carcinoma Cells. Plos One. 2014;9(6). doi: 10.1371/journal.pone.0100372. PubMed PMID: WOS:000338508200098.

51. Song HT, Tian ZN, Qin Y, Yao GD, Fu SB, Geng JS. Astrocyte elevated gene-1 activates MMP9 to increase invasiveness of colorectal cancer. Tumor Biology. 2014;35(7):6679-85. doi: 10.1007/s13277-014-1883-3. PubMed PMID: WOS:000339736300065.

52. Song HT, Qin Y, Yao GD, Tian ZN, Fu SB, Geng JS. Astrocyte elevated gene-1 mediates glycolysis and tumorigenesis in colorectal carcinoma cells via AMPK signaling. Mediators of inflammation. 2014;2014:287381. Epub 2014/05/16. doi: 10.1155/2014/287381. PubMed PMID: 24829520; PubMed Central PMCID: PMCPmc4009221.

53. Li C, Wu X, Zhang H, Yang G, Hao M, Sheng S, et al. A Huaier polysaccharide restrains hepatocellular carcinoma growth and metastasis by suppression angiogenesis. International journal of biological macromolecules. 2015;75:115-20. Epub 2015/01/20. doi: 10.1016/j.ijbiomac.2015.01.016. PubMed PMID: 25597429.

54. Li C, Wu X, Zhang H, Yang G, Hao M, Sheng S, et al. A Huaier polysaccharide reduced metastasis of human hepatocellular carcinoma SMMC-7721 cells via modulating AUF-1 signaling pathway. Tumour biology : the journal of the International Society for Oncodevelopmental Biology and Medicine. 2015. Epub 2015/03/20. doi: 10.1007/s13277-015-3314-5. PubMed PMID: 25787750.

55. Srivastava J, Robertson CL, Gredler R, Siddiq A, Rajasekaran D, Akiel MA, et al. Astrocyte Elevated Gene-1 (AEG-1) Contributes to Non-thyroidal Illness Syndrome (NTIS) Associated with Hepatocellular Carcinoma (HCC). The Journal of biological chemistry. 2015;290(25):15549-58. Epub 2015/05/07. doi: 10.1074/jbc.M115.649707. PubMed PMID: 25944909; PubMed Central PMCID: PMCPmc4505468.

56. Zhang C, Li HZ, Qian BJ, Liu CM, Guo F, Lin MC. MTDH/AEG-1-based DNA vaccine suppresses metastasis and enhances chemosensitivity to paclitaxel in pelvic lymph node metastasis. Biomedicine & pharmacotherapy = Biomedecine & pharmacotherapie. 2015;70:217-26. Epub 2015/03/18. doi: 10.1016/j.biopha.2015.01.028. PubMed PMID: 25776504.

57. Rajasekaran D, Srivastava J, Ebeid K, Gredler R, Akiel M, Jariwala N, et al. Combination of Nanoparticle-Delivered siRNA for Astrocyte Elevated Gene-1 (AEG-1) and All-trans Retinoic Acid (ATRA): An Effective Therapeutic Strategy for Hepatocellular Carcinoma (HCC). Bioconjugate chemistry. 2015. Epub 2015/06/17. doi: 10.1021/acs.bioconjchem.5b00254. PubMed PMID: 26079152.

58. Xia L, Yu G, Zeng X, Li S, Bing Y, Li T, et al. Metadherin promotes gastric carcinoma metastasis through induction of epithelial-mesenchymal transition. Practical Oncology Journal. 2015;(1):39-43. doi: 10.11904/j.issn.1002-3070.2015.01.008.

59. Li L, Yu T, Yu X, Li D, Wang J. Effect of lobaplatin on proliferation, apoptosis and expression of Metadherin in human gastric cancer SGC-7901 cells Chin J Exp Surg. 2012;29(12):2479-81. doi: 10.3760/cma.j.issn.1001-9030.2012.12.048.

60. Huang S, Wu B, Li D, Liu B, Deng G, Zhang K. AEG-1 affects expression of HIF-1 α and miR-34a in colorectal cancer cells. World Chinese Journal of Digestology. 2014;(18):2532-8.

61. Li X, Wang H, Shen B, Song X. The effects of down-regulation of AEG-1 on prolification and apoptosis of HCC. China Rural Health 2014;(z2):68-9.

62. Wang H, Duan C, Qin R, Li W, Hou L, Duan S, et al. Effect of down-regulation of astrocyte elevated gene-1 expression by small interfering RNA inhibits cell proliferation and induces apoptosis in human hepatocellular carcinoma HepG2 cells. Chin J Exp Surg. 2014;31(5):982-4. doi: 10.3760/cma.j.issn.1001-9030.2014.05.021.

63. Huang S. The Study of AEG-1 Biological Functions and Its Impact on microRNAs Expression in Colorectal Cancer [Master]: NanFang Medical University; 2014.

64. Baygi ME, Nikpour P. Deregulation of MTDH gene expression in gastric cancer. Asian Pacific journal of cancer prevention : APJCP. 2012;13(6):2833-6. Epub 2012/09/04. PubMed PMID: 22938468.

65. Motalleb G, Gholipour N, Samaei NM. Association of the human astrocyte elevated gene-1 promoter variants with susceptibility to hepatocellular carcinoma. Medical oncology (Northwood, London, England). 2014;31(4):916. Epub 2014/03/25. doi: 10.1007/s12032-014-0916-5. PubMed PMID: 24659263.

66. Ma W, Liu J, Shi J, Qi J. Expression and significance of L-selectin and MTDH in gastric carcinoma. JOURNAL OF CHENGDE MEDICAL COLLEGE. 2014;(05):371-3.

67. Yao Q, Wang L, Huang M, Rao B, Huang J, Wu X, et al. InvestigatingthecorrelationbetweenMTDH/AEG-1 gene 5'-UTR polymorphism and genetic susceptibility to sporadic colorectal cancer. Chinese Journal of Pathophysiology 2010;(08):1468-73.

68. Steinert R, Gastinger I, Ridwelski K, Ptok H, Wolff S, Meyer F, et al. [Surgical treatment of carcinomas of the oesophagogastric junction - results achieved in multicentre studies]. Zentralblatt fur Chirurgie. 2013;138(4):403-9. Epub 2013/08/21. doi: 10.1055/s-0033-1350712. PubMed PMID: 23950077.

69. Sarkar D, Fisher PB. AEG-1/MTDH/LYRIC: clinical significance. Advances in cancer research. 2013;120:39-74. Epub 2013/07/31. doi: 10.1016/b978-0-12-401676-7.00002-4. PubMed PMID: 23889987; PubMed Central PMCID: PMCPmc3924591.

70. Meng XB, Thiel KW, Leslie KK. Drug Resistance Mediated by AEG-1/MTDH/LYRIC. In: Sarkar D, Fisher PB, editors. Aeg-1/Mtdh/Lyric Implicated in Multiple Human Cancers. Advances in Cancer Research. 1202013. p. 135-57.

71. Sarkar D. AEG-1/MTDH/LYRIC in liver cancer. Advances in cancer research. 2013;120:193-221. Epub 2013/07/31. doi: 10.1016/b978-0-12-401676-7.00007-3. PubMed PMID: 23889992; PubMed Central PMCID: PMCPmc3924581.

72. Zhu HD, Liao JZ, He XX, Li PY. The emerging role of astrocyte-elevated gene-1 in hepatocellular carcinoma (Review). Oncology reports. 2015;34(2):539-46. Epub 2015/06/03. doi: 10.3892/or.2015.4024. PubMed PMID: 26035424.

73. Shi X, Wang X. The role of MTDH/AEG-1 in the progression of cancer. International journal of clinical and experimental medicine. 2015;8(4):4795-807. Epub 2015/07/02. PubMed PMID: 26131054; PubMed Central PMCID: PMCPmc4484038.

74. Chen L. Role of metadherin in the progress of tumors. International Journal of Pathology and Clinical Medicine. 2010;30(3):243-6. doi: 10.3969/j.issn.1673-2588.2010.03.013.

75. Liu C, Qi J, Wang H. The function of CD151, MTDH in gastric carcinoma. Joural of ChenDe Medical College. 2015;(02):146-8.

76. Yang C, Lu X. Correlation of MTDH, miR-26a with tumors. Journal of XinJiang Medical University. 2015;(01):114-21.

77. Li C, Long J, Sheng S, Sun Y, Zhang H, Li J, et al. The biology function of Astrocyte elevated gene -1 (AEG-1) in hepatocellular carcinoma. Journal of Practical oncology. 2014;(1):61-5. doi: 10.3969/j.issn.1002-3070.2014.01.013.

78. Zhao L, Xin Y, Xiao Y. Latest research progress of AEG-1 and malignant tumor of digestive system. Mordern Oncology. 2012;(11):2419-22.
